# Supplementary material for: Profile of the bile acid FXR-FGF15 pathway in the glucolipid metabolism disorder of diabetic mice suffering from chronic stress
Source: PeerJ. 2023 Nov 15;11:e16407. doi: 10.7717/peerj.16407 (PMC10656902; doi:10.7717/peerj.16407)
Supplement: Table S1 [file peerj-11-16407-s001.docx]

**Table S1. The schedule of stressors.**

| **Sunday** | **Monday** | **Tuesday** | **Wednesday** | **Thursday** | **Friday** | **Saturday** |
| --- | --- | --- | --- | --- | --- | --- |
|  |  |  |  |  | Cage vibration + Restraint stress | Damp sawdust + Cage tilting |
| Noise +  No sawdust | Swimming at 4°C  + Cycle disturbances | Cage tilting + Noise | Restraint stress +  Cage vibration | Damp sawdust + Noise | No sawdust +  Noise | Swimming at 4°C +  Cycle disturbances |
| Restraint stress +  Cage vibration | Swimming at 4°C +  Cycle disturbances | Cage tilting +  Noise | Restraint stress +  Cage vibration | Cycle disturbance +  Noise | Swimming at 4°C +  Cycle disturbances | Restraint stress +  Cage vibration |
| Cage vibration +  Cycle disturbances | Damp sawdust + Noise | No sawdust +  Cage vibration | Cage tilting +  Cycle disturbances | Swimming at 4°C +  Noise | Restraint stress + Cage vibration | Noise +  Cage tilting |
| Swimming at 4°C +  Cycle disturbances | Restraint stress +  Cage vibration | Cage tilting +  Noise | Damp sawdust + Restraint stress | No sawdust +  Noise |  |  |
