# Supplementary material for: Profile of the bile acid FXR-FGF15 pathway in the glucolipid metabolism disorder of diabetic mice suffering from chronic stress
Source: PeerJ. 2023 Nov 15;11:e16407. doi: 10.7717/peerj.16407 (PMC10656902; doi:10.7717/peerj.16407)
Supplement: Table S2 [file peerj-11-16407-s002.docx]

**Table S2. List of qPCR primer sequences.**

| **Genes** | **Forward primer (5’→3’)** | **Reverse primer (5’→3’)** |
| --- | --- | --- |
| FXR | ATGGCAACCAGTCATGTACAGA | ATTGAAAATCTCCGCCGAACGA |
| SHP | TAGATCTCTTCTTCCGCCCTA | AGACTCCATTCCACGGGTCA |
| FGF15 | GACTGCGAGGAGGACCAAAA | CAGCCCGTATATCTTGCCGT |
| ACTB | CATCCGTAAAGACCTCTATGCCAAC | ATGGAGCCACCGATCCACA |

*FXR*, farnesoid X receptor; *SHP*, small heterodimer partner; *FGF15*, fibroblast growth factor 15; *ACTB*, β-actin.
