# Supplementary figures and images for: Profile of the bile acid FXR-FGF15 pathway in the glucolipid metabolism disorder of diabetic mice suffering from chronic stress
Source: PeerJ. 2023 Nov 15;11:e16407. doi: 10.7717/peerj.16407 (PMC10656902; doi:10.7717/peerj.16407)

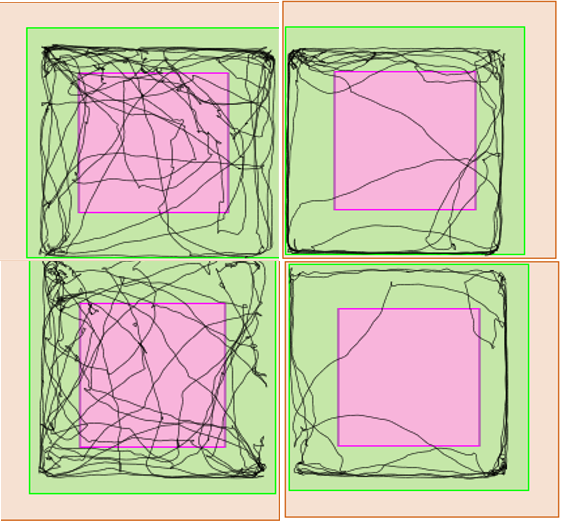

Supplement: Figure S1 [file peerj-11-16407-s004.zip › Figure 1/Behavial tests/Open field test/Tracking map.tif]

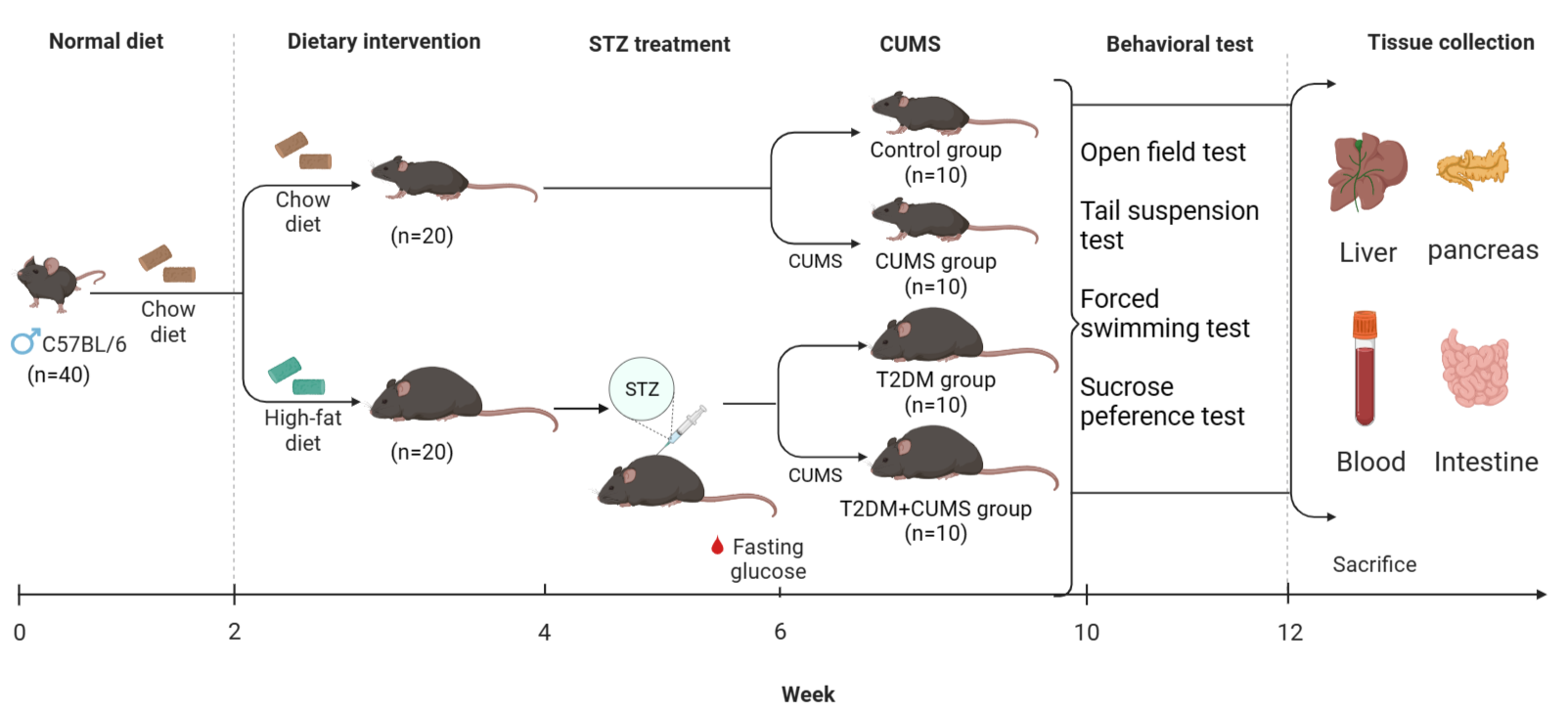

Supplement: Figure S1 [file peerj-11-16407-s004.zip › Figure 1/Model establishment.tif]

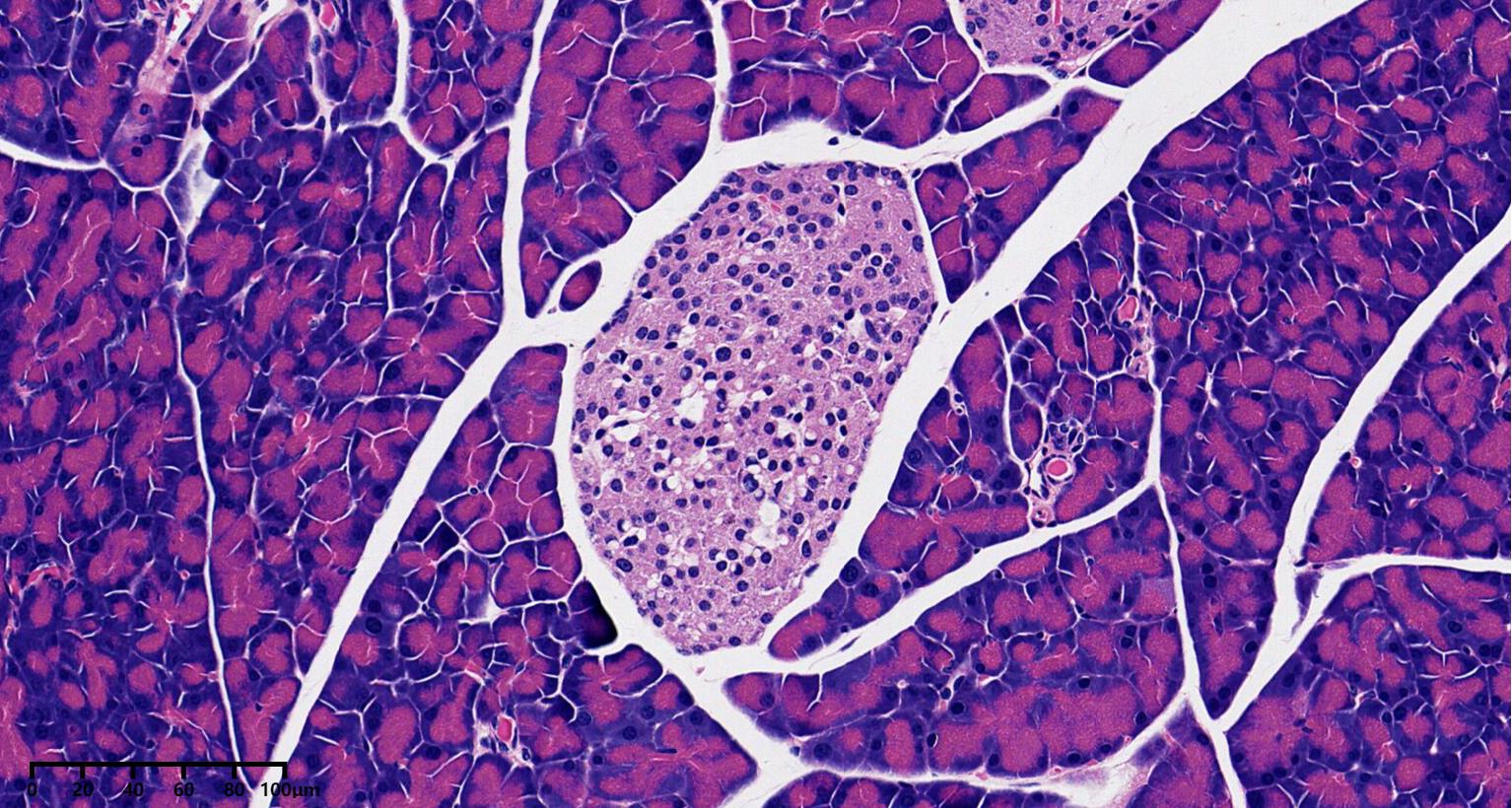

Supplement: Figure S3 [file peerj-11-16407-s006.zip › Figure 3/pancreas HE/CUMS pancreas HE_20.00X_20221227092422.jpg]

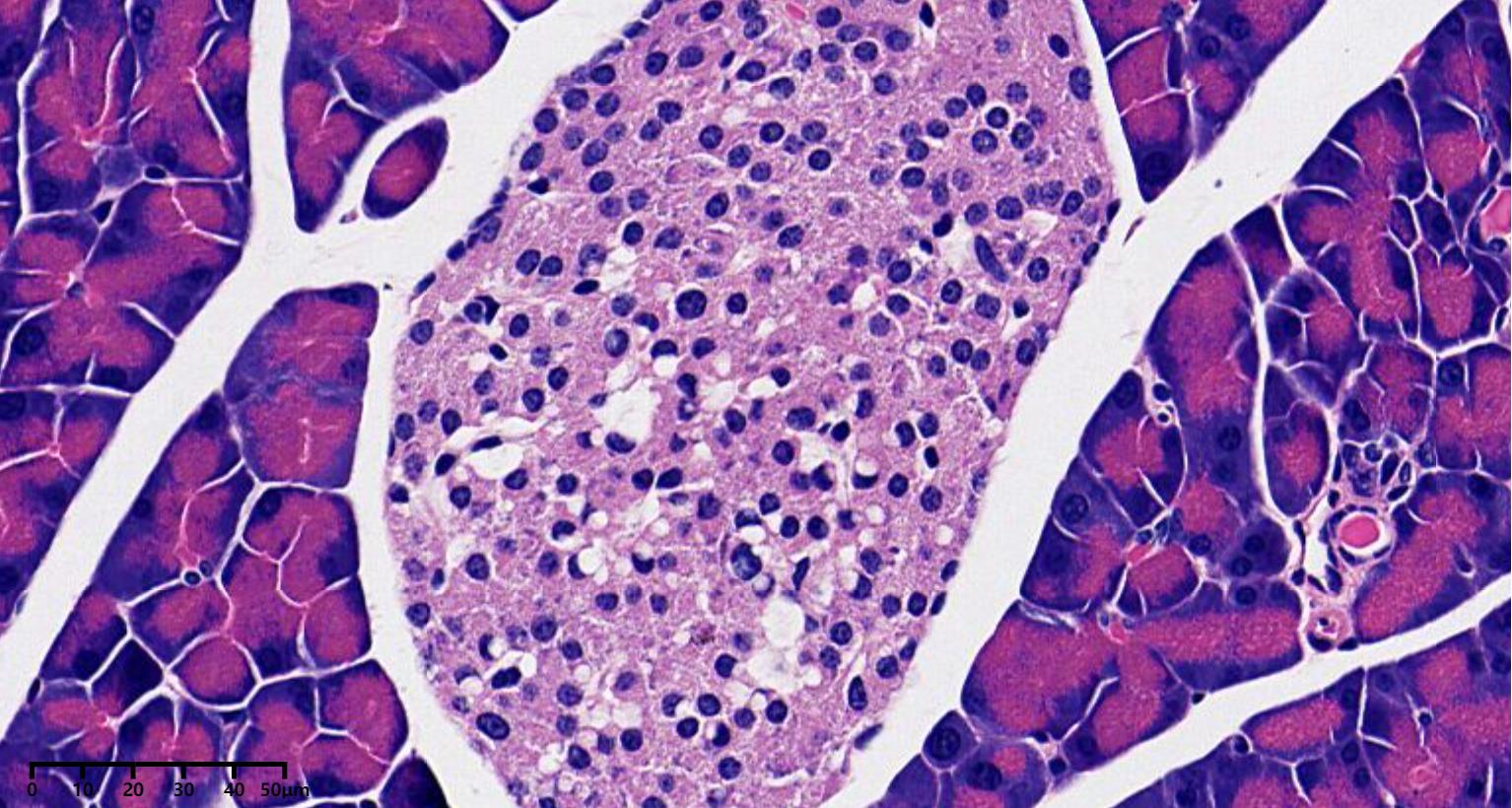

Supplement: Figure S3 [file peerj-11-16407-s006.zip › Figure 3/pancreas HE/CUMS pancreas HE_40.00X_20221227092434.jpg]

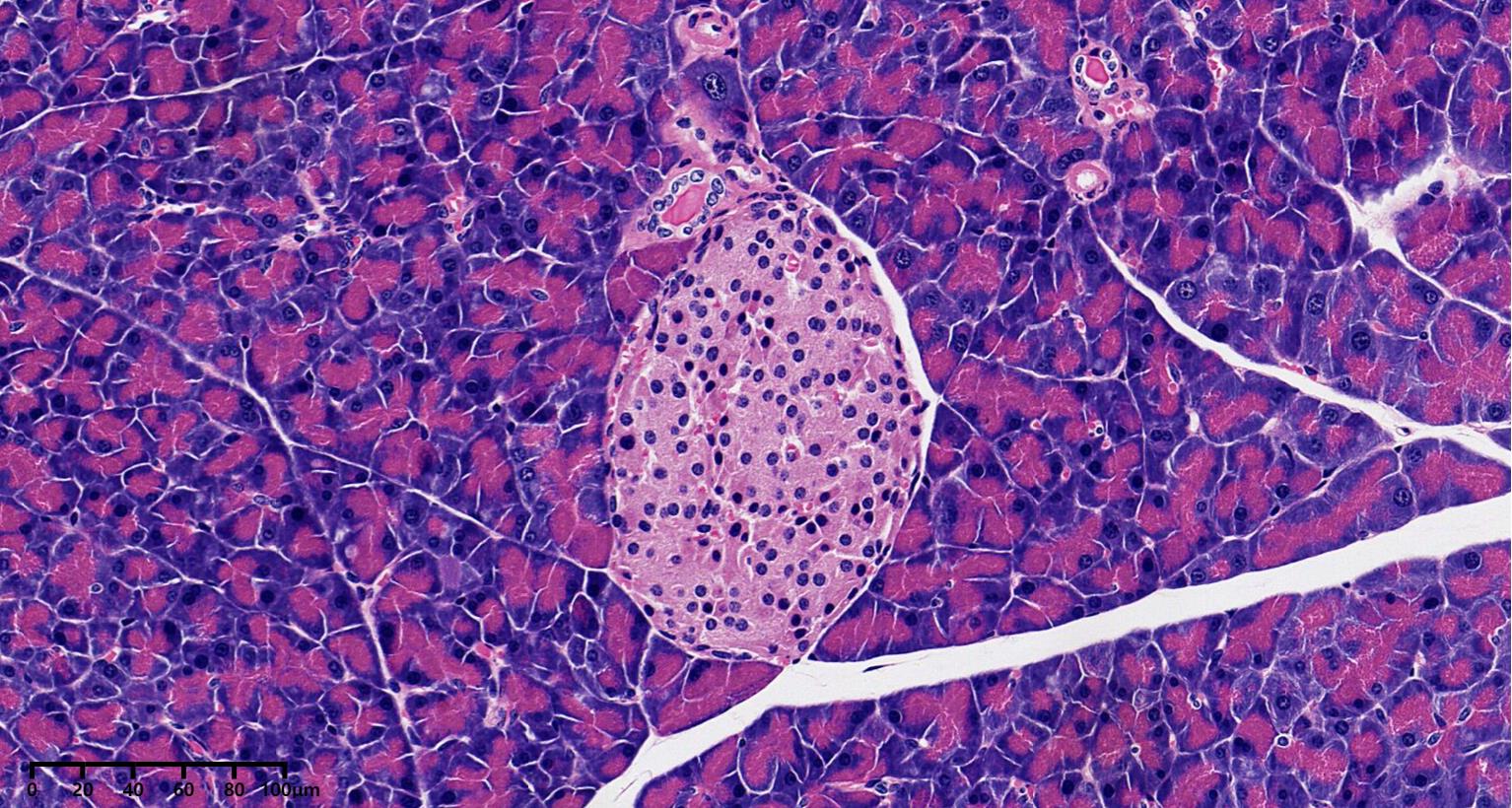

Supplement: Figure S3 [file peerj-11-16407-s006.zip › Figure 3/pancreas HE/Control pancreas HE_20.00X_20221227092332.jpg]

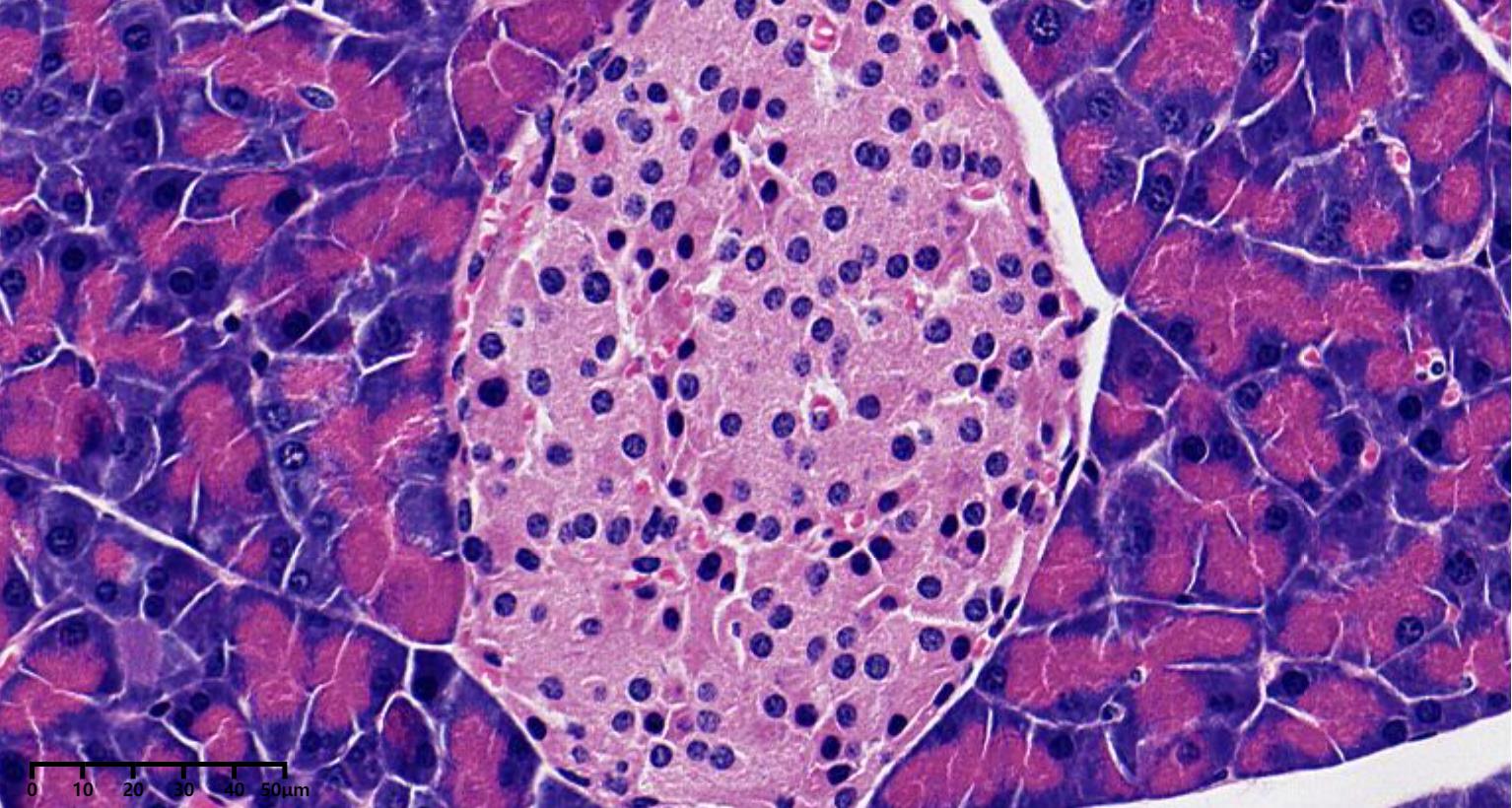

Supplement: Figure S3 [file peerj-11-16407-s006.zip › Figure 3/pancreas HE/Control pancreas HE_40.00X_20221227092359.jpg]

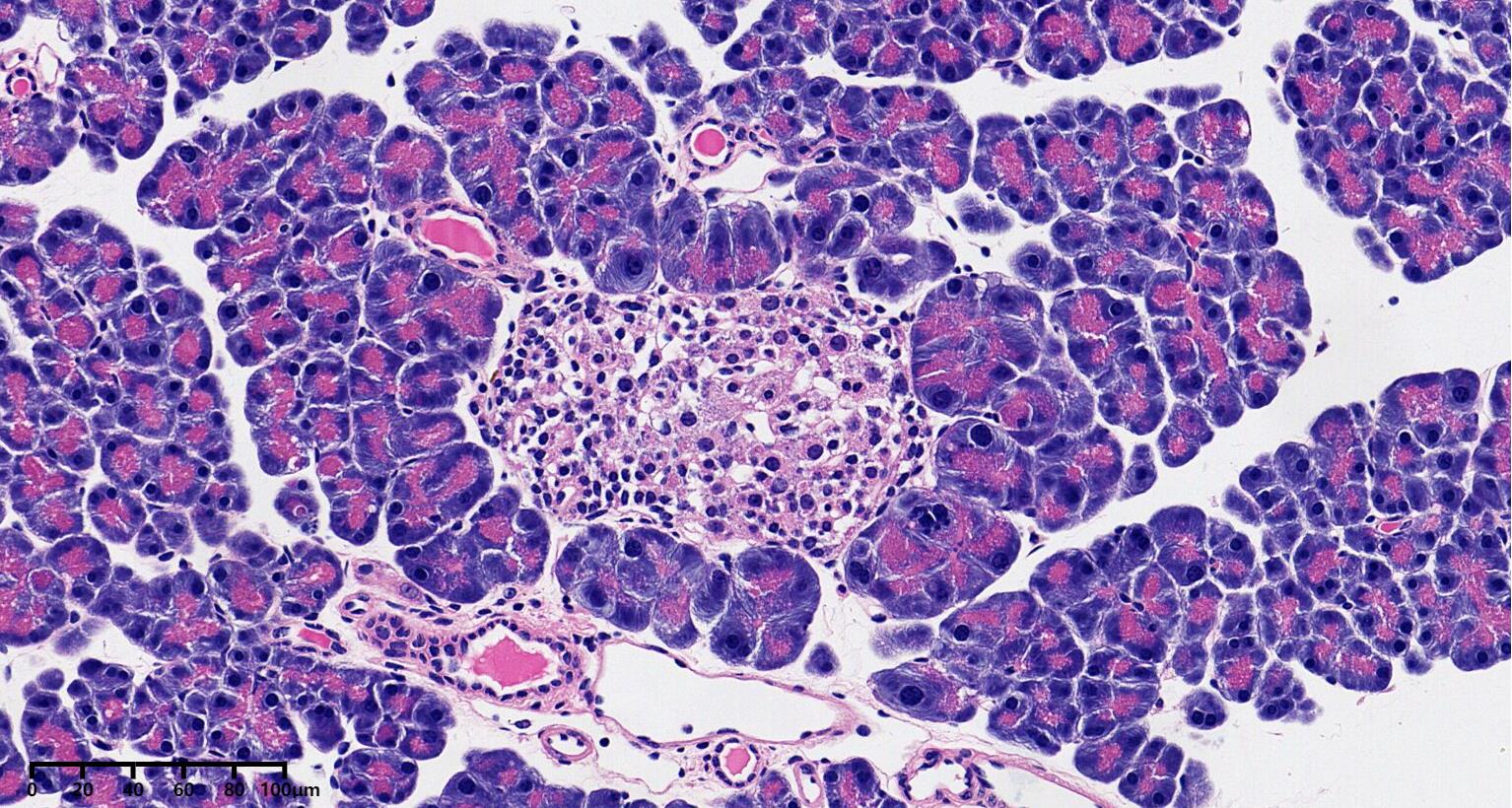

Supplement: Figure S3 [file peerj-11-16407-s006.zip › Figure 3/pancreas HE/T2DM pancreas HE_20.00X_20221227093514.jpg]

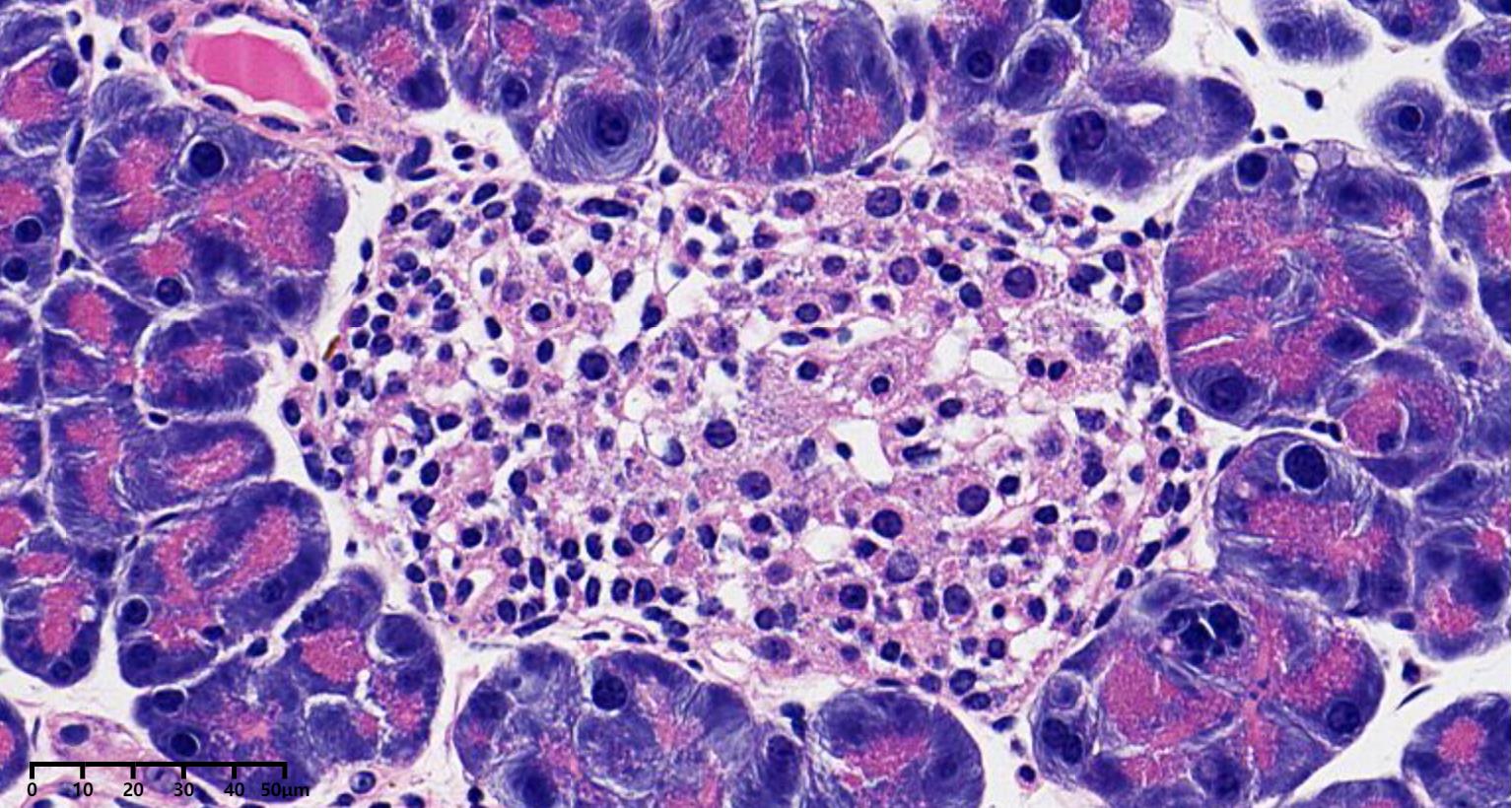

Supplement: Figure S3 [file peerj-11-16407-s006.zip › Figure 3/pancreas HE/T2DM pancreas HE_40.00X_20221227093525.jpg]

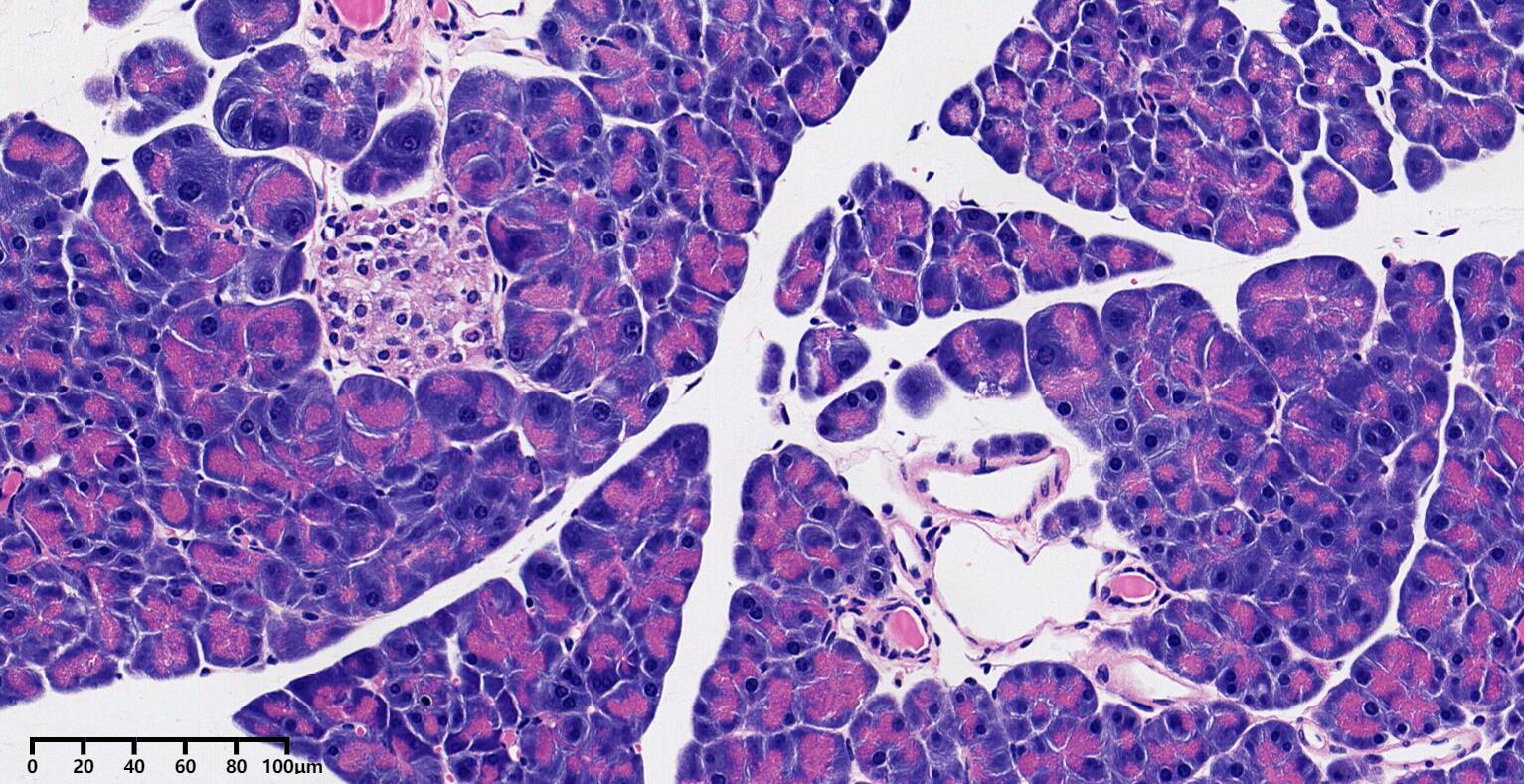

Supplement: Figure S3 [file peerj-11-16407-s006.zip › Figure 3/pancreas HE/T2DM+CUMS pancreas HE_20.00X_20221227094127.jpg]

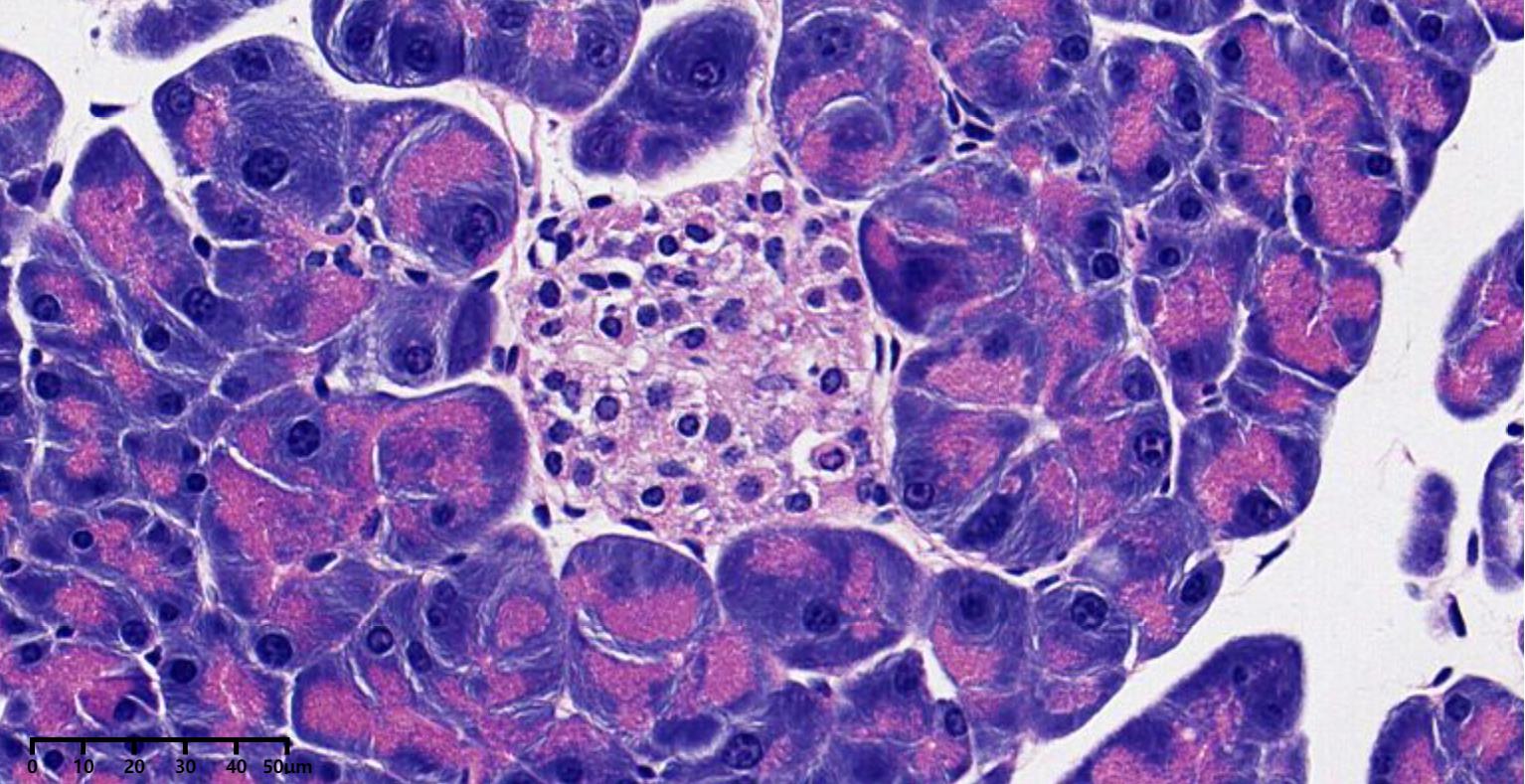

Supplement: Figure S3 [file peerj-11-16407-s006.zip › Figure 3/pancreas HE/T2DM+CUMS pancreas HE_40.00X_20221227094148.jpg]

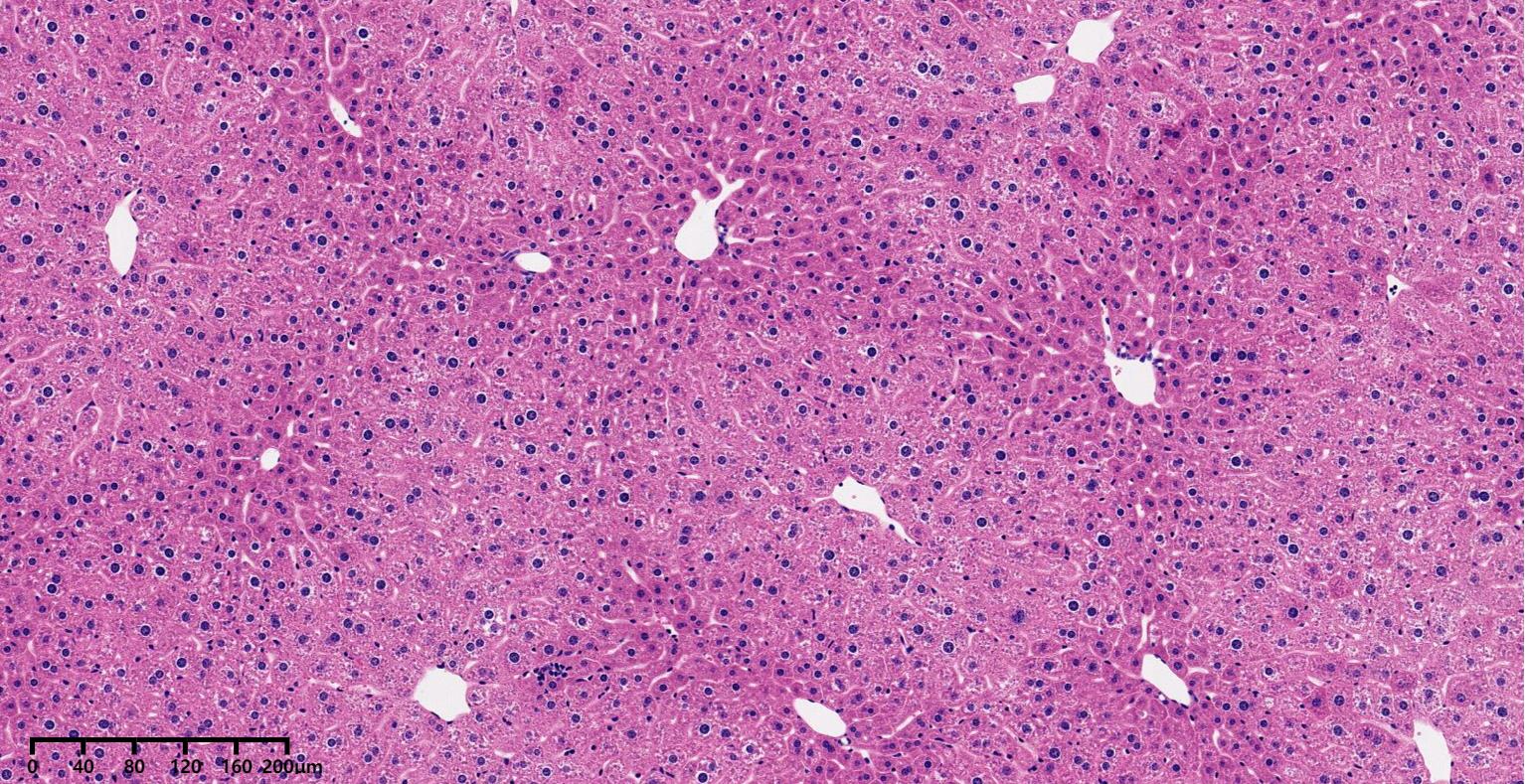

Supplement: Figure S4 [file peerj-11-16407-s007.zip › Figure 4/Liver HE/CUMS liver_10.00X_20230227115028.jpg]

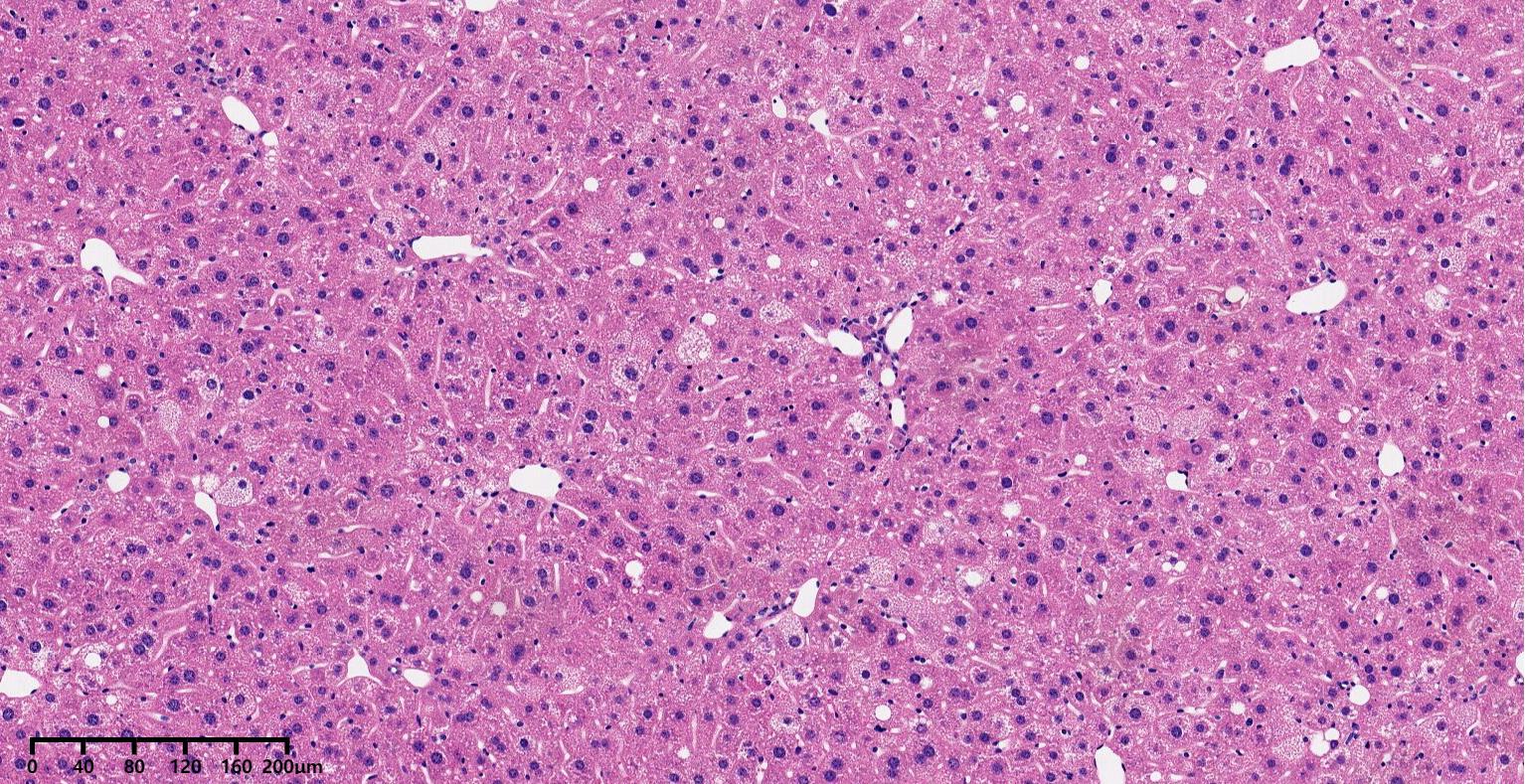

Supplement: Figure S4 [file peerj-11-16407-s007.zip › Figure 4/Liver HE/CUMS liver_10.00X_20230227115101.jpg]

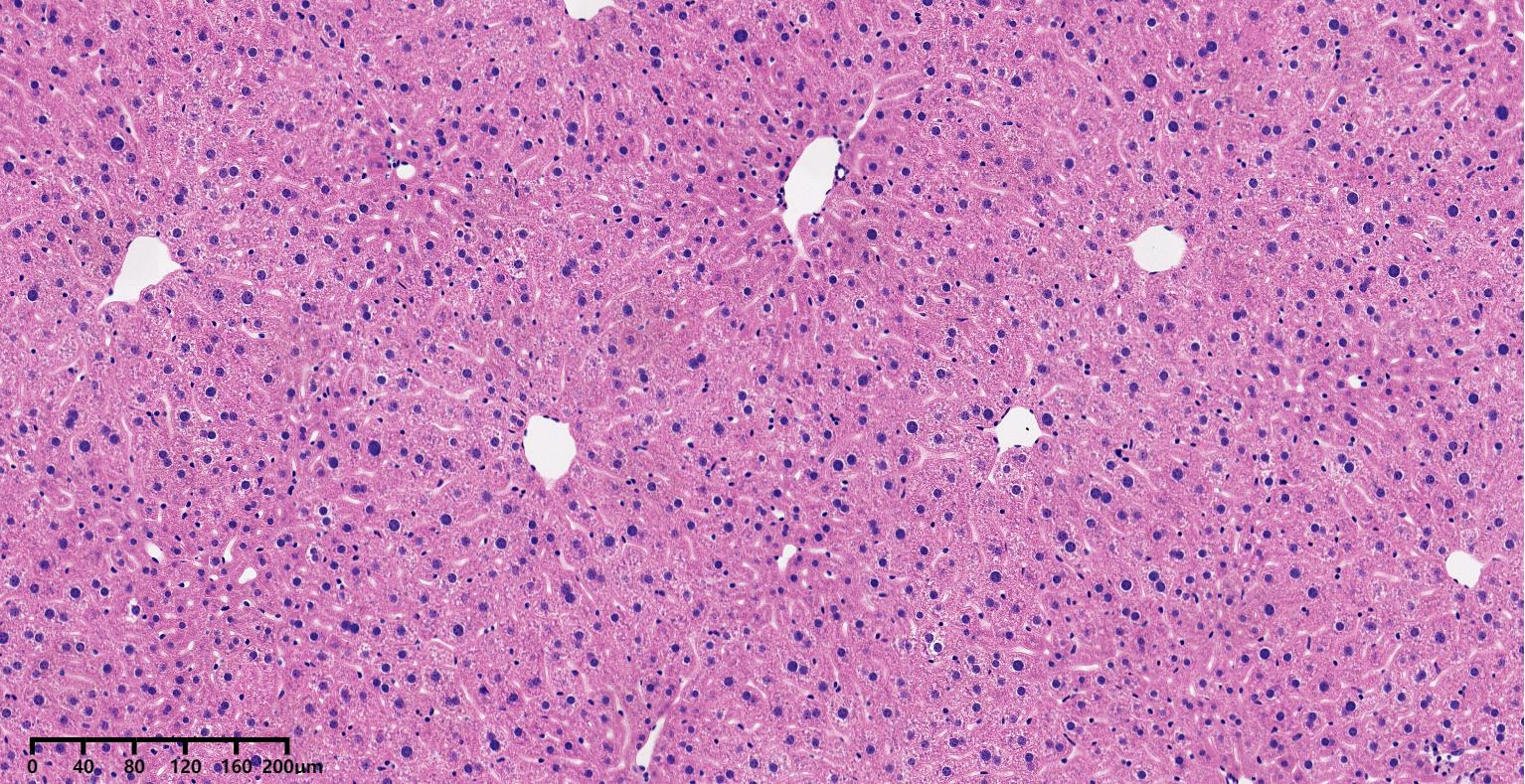

Supplement: Figure S4 [file peerj-11-16407-s007.zip › Figure 4/Liver HE/CUMS liver_10.00X_20230227115111.jpg]

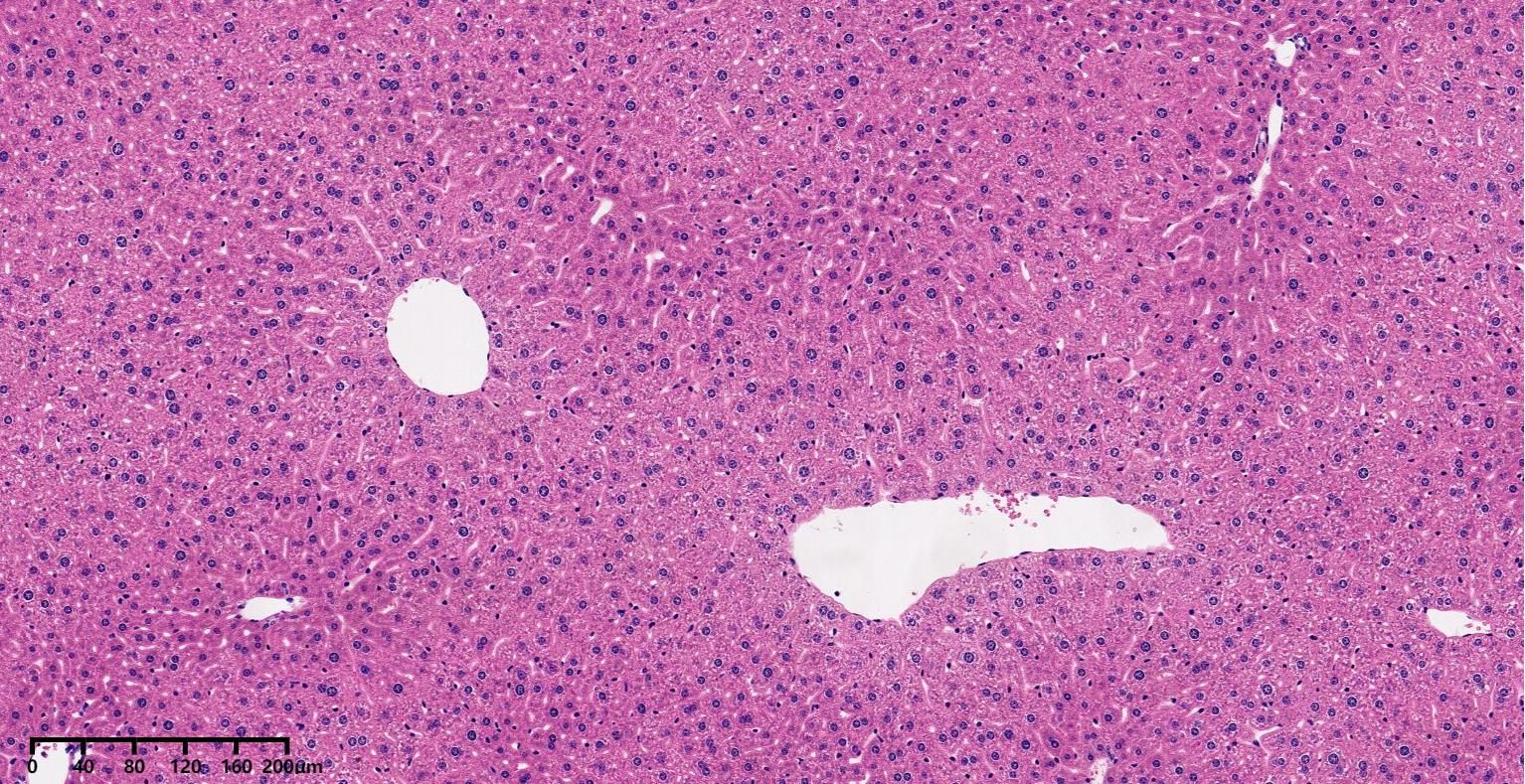

Supplement: Figure S4 [file peerj-11-16407-s007.zip › Figure 4/Liver HE/CUMS liver_10.00X_20230227115159.jpg]

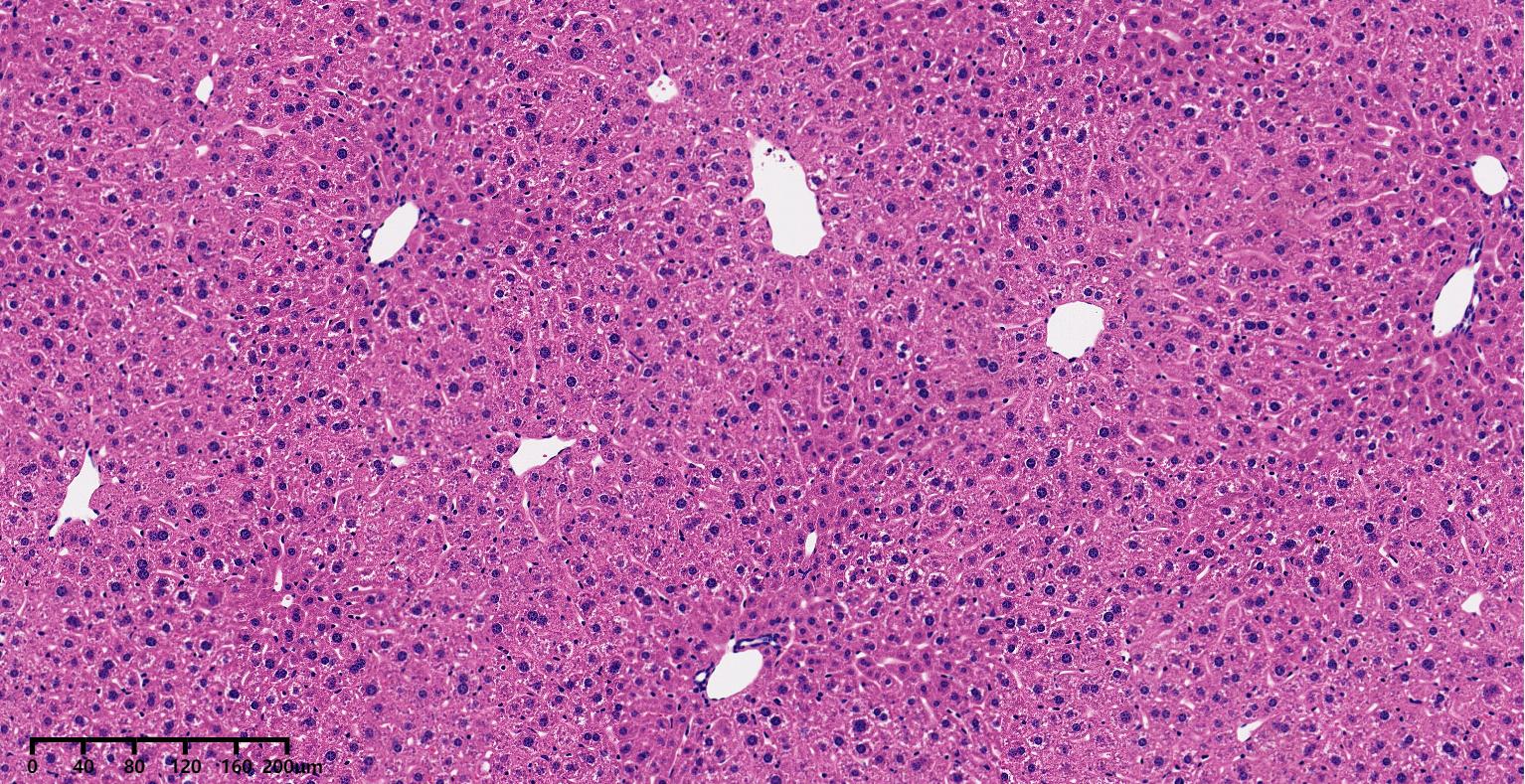

Supplement: Figure S4 [file peerj-11-16407-s007.zip › Figure 4/Liver HE/Control liver_10.00X_20230227115411.jpg]

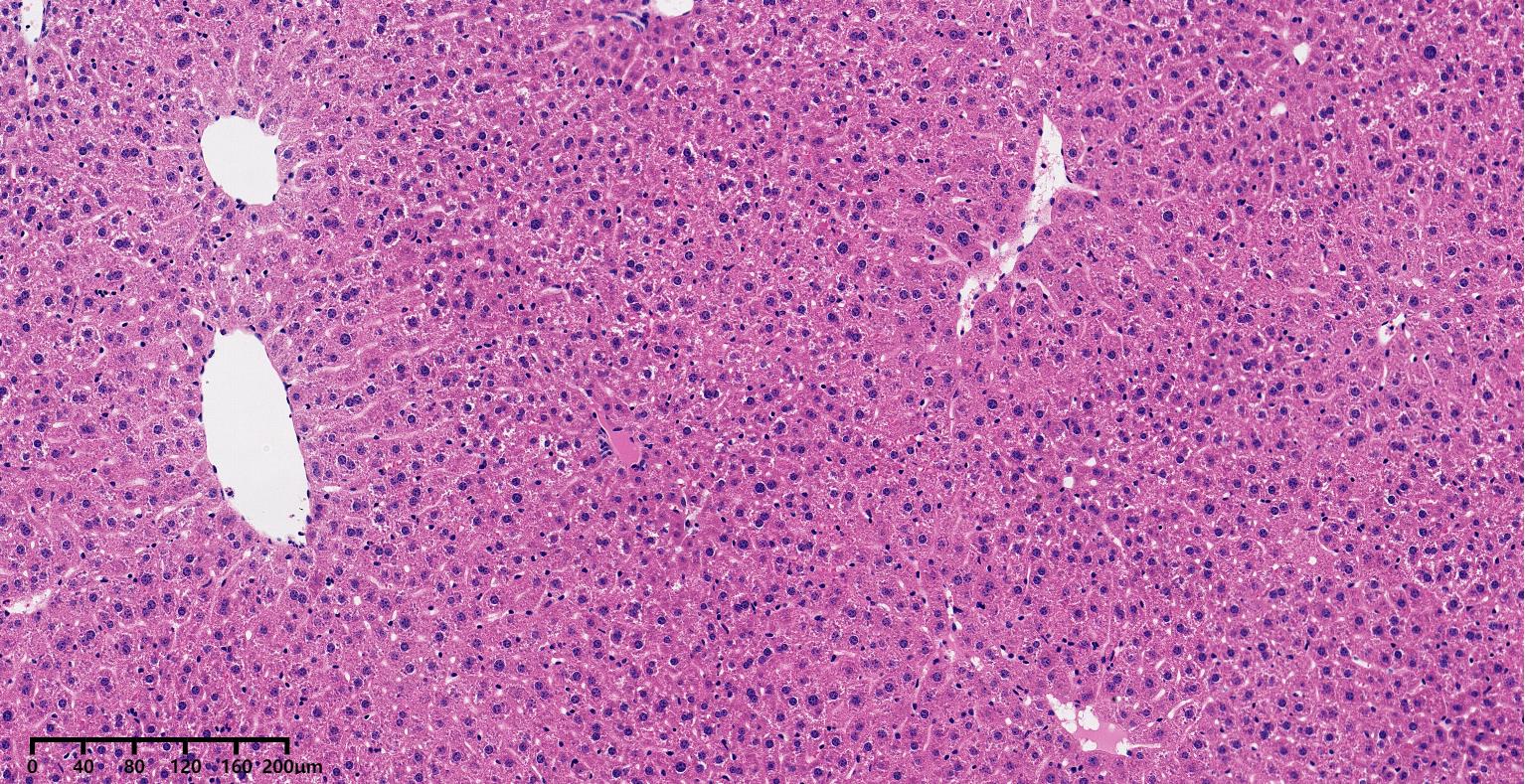

Supplement: Figure S4 [file peerj-11-16407-s007.zip › Figure 4/Liver HE/Control liver_10.00X_20230227115447.jpg]

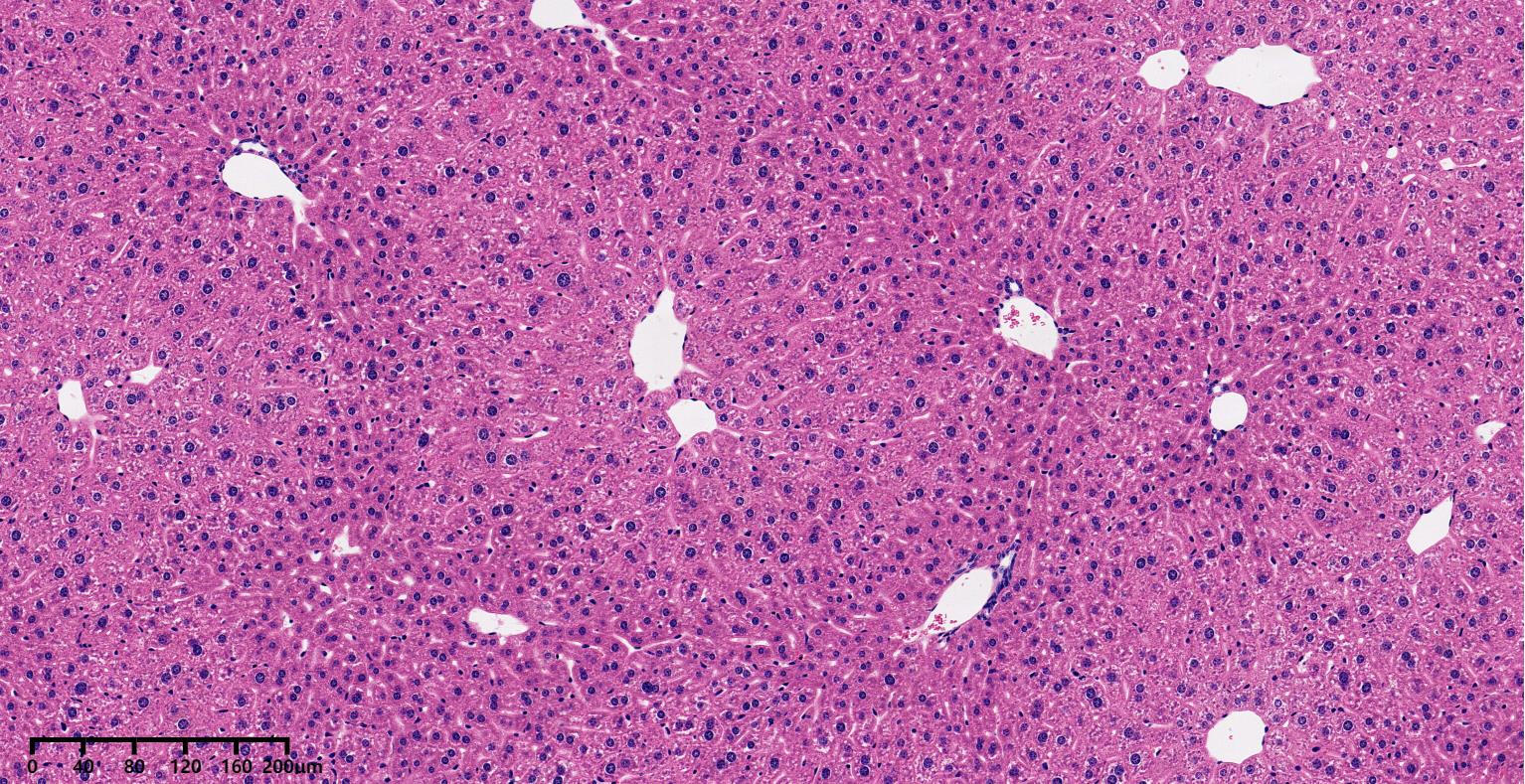

Supplement: Figure S4 [file peerj-11-16407-s007.zip › Figure 4/Liver HE/Control liver_10.00X_20230227115516.jpg]

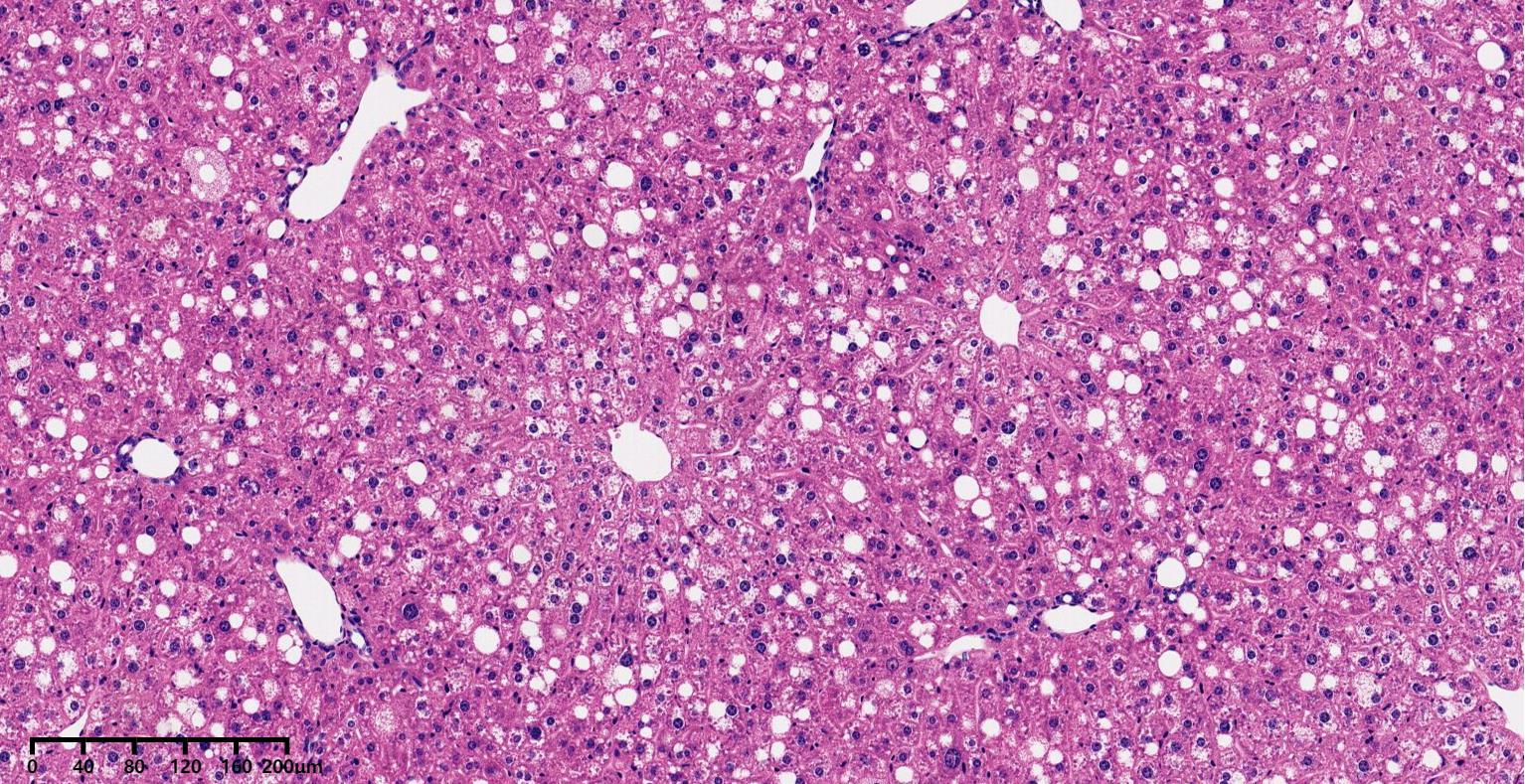

Supplement: Figure S4 [file peerj-11-16407-s007.zip › Figure 4/Liver HE/DCD45 liver_10.00X_20230227115734.jpg]

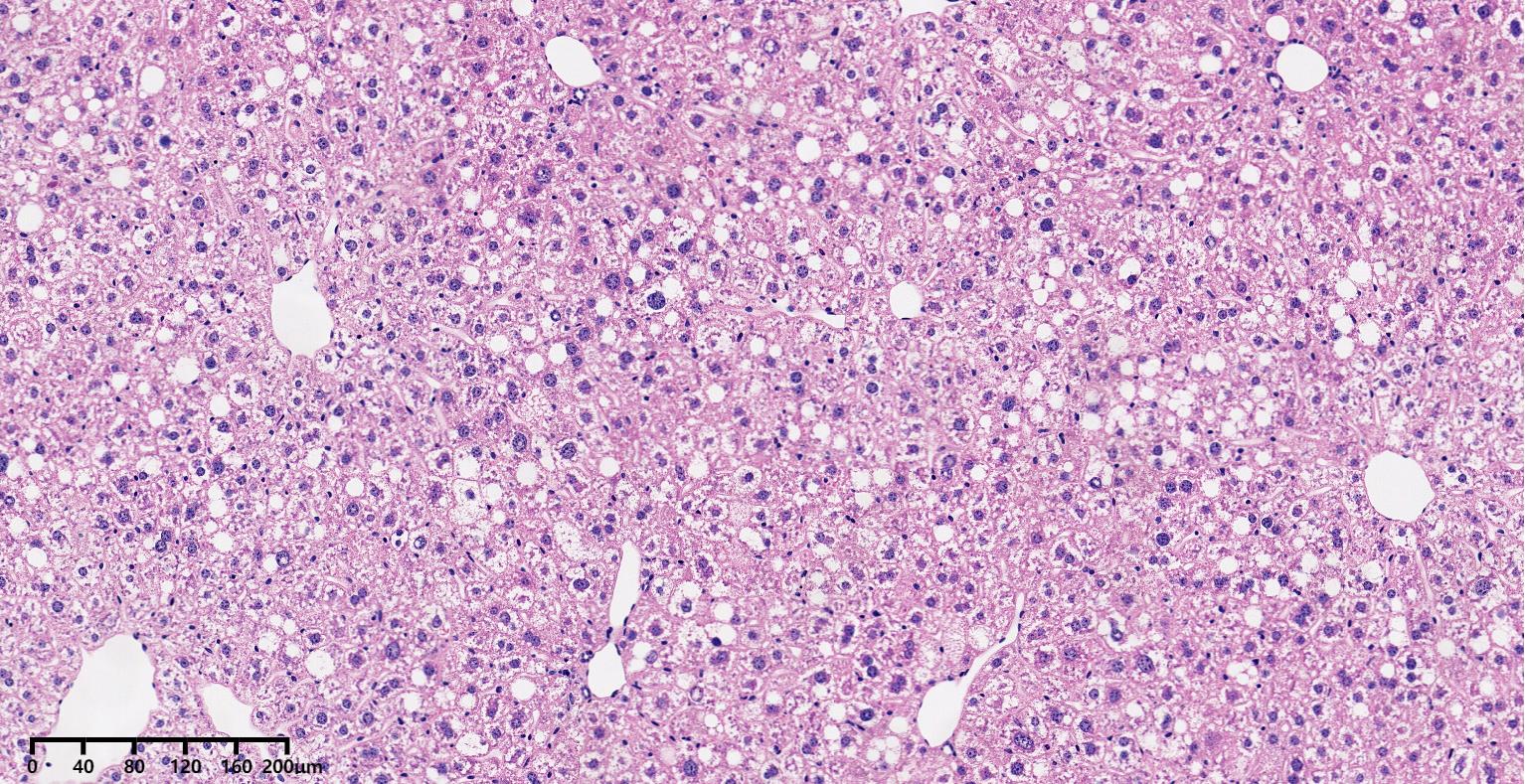

Supplement: Figure S4 [file peerj-11-16407-s007.zip › Figure 4/Liver HE/T2DM liver_10.00X_20230227115546.jpg]

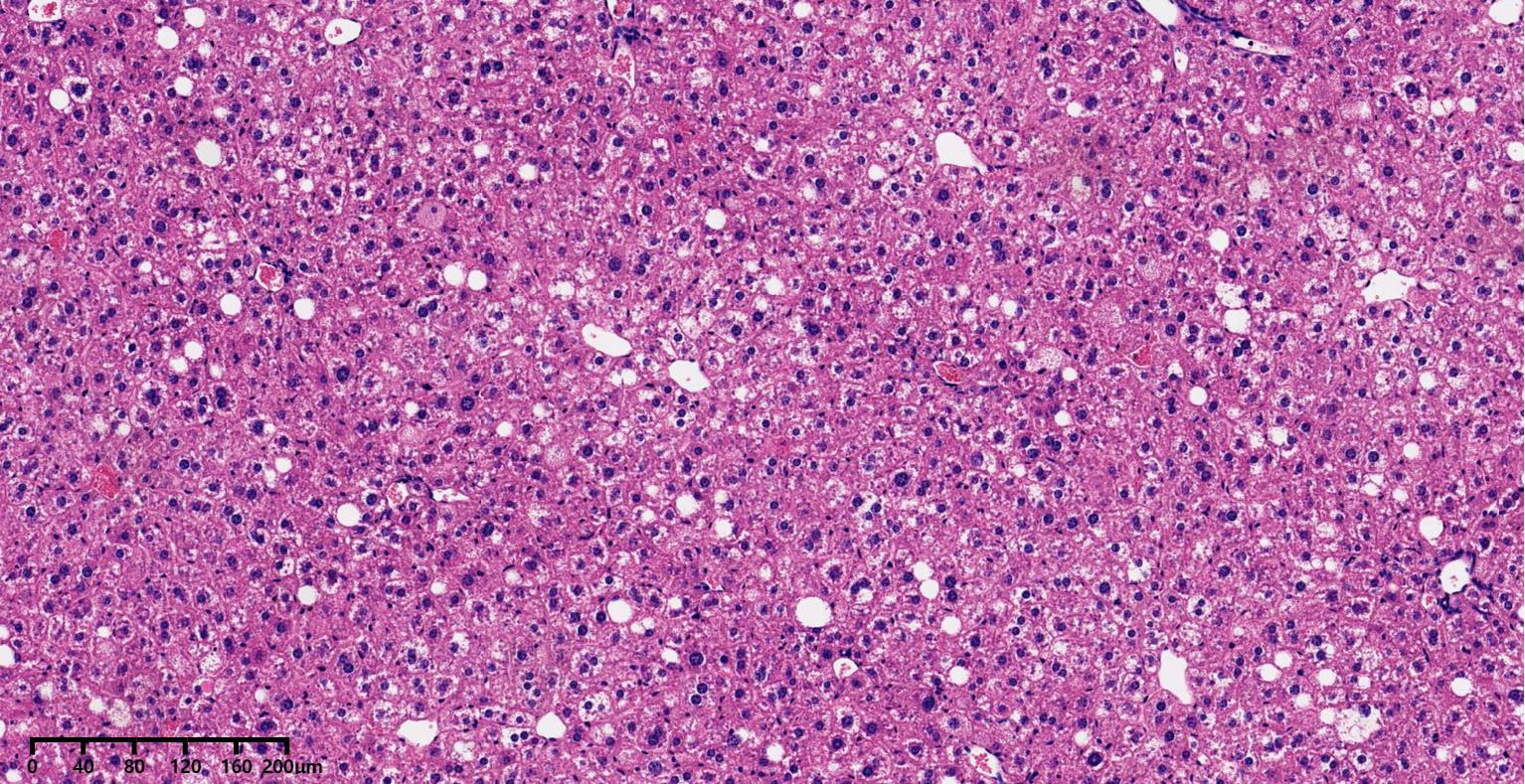

Supplement: Figure S4 [file peerj-11-16407-s007.zip › Figure 4/Liver HE/T2DM liver_10.00X_20230227115613.jpg]

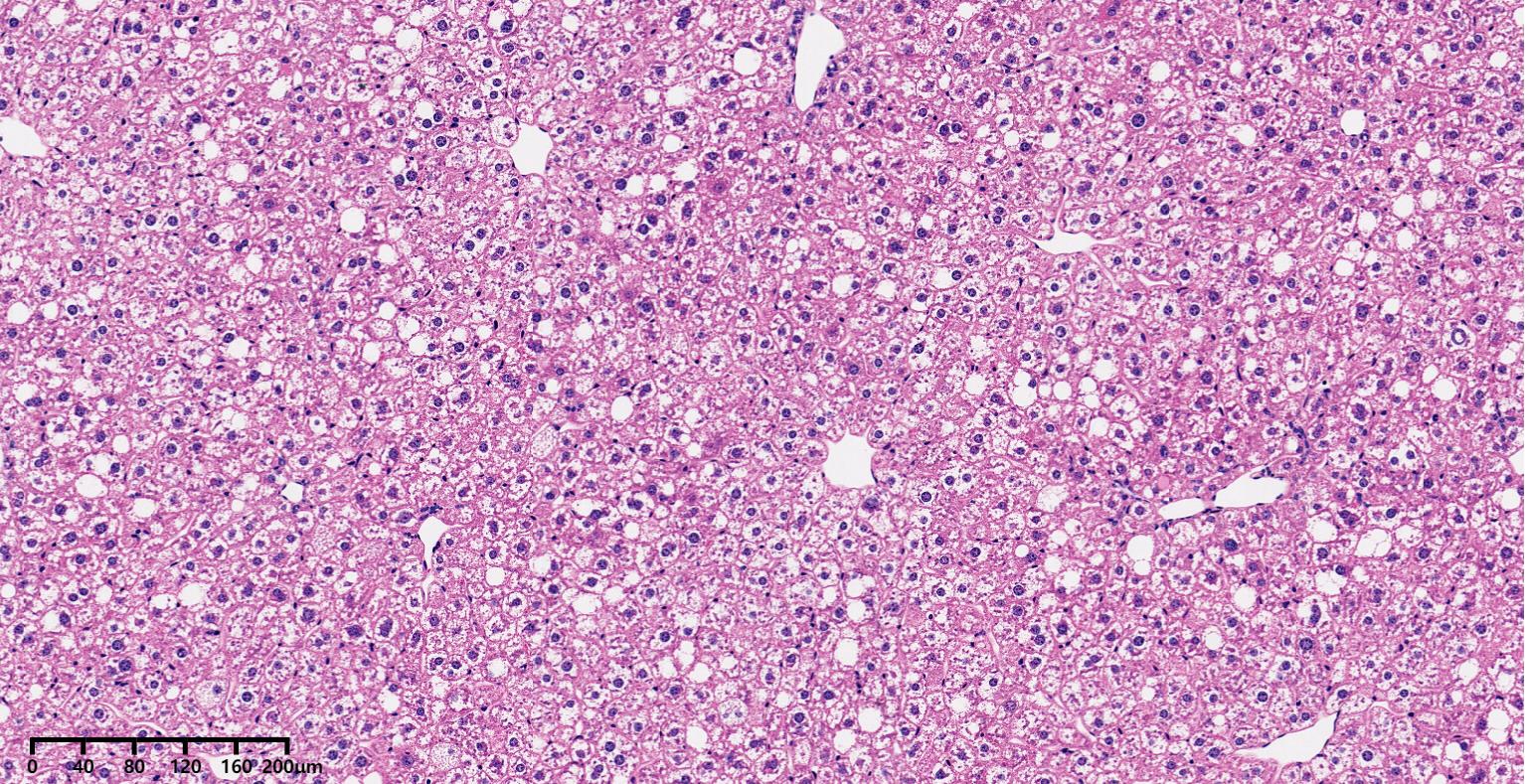

Supplement: Figure S4 [file peerj-11-16407-s007.zip › Figure 4/Liver HE/T2DM liver_10.00X_20230227115632.jpg]

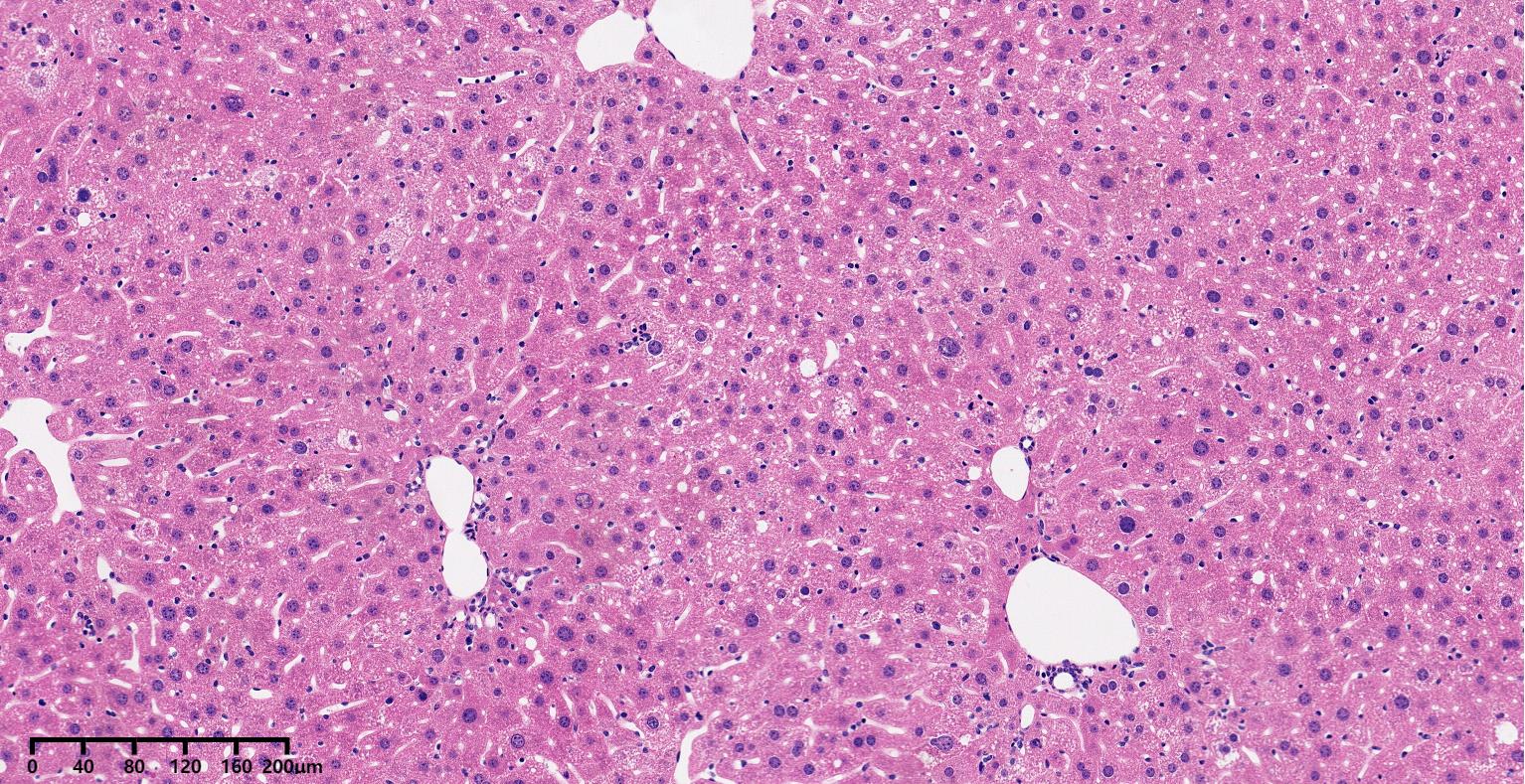

Supplement: Figure S4 [file peerj-11-16407-s007.zip › Figure 4/Liver HE/T2DM+CUMS liver_10.00X_20230227115711.jpg]

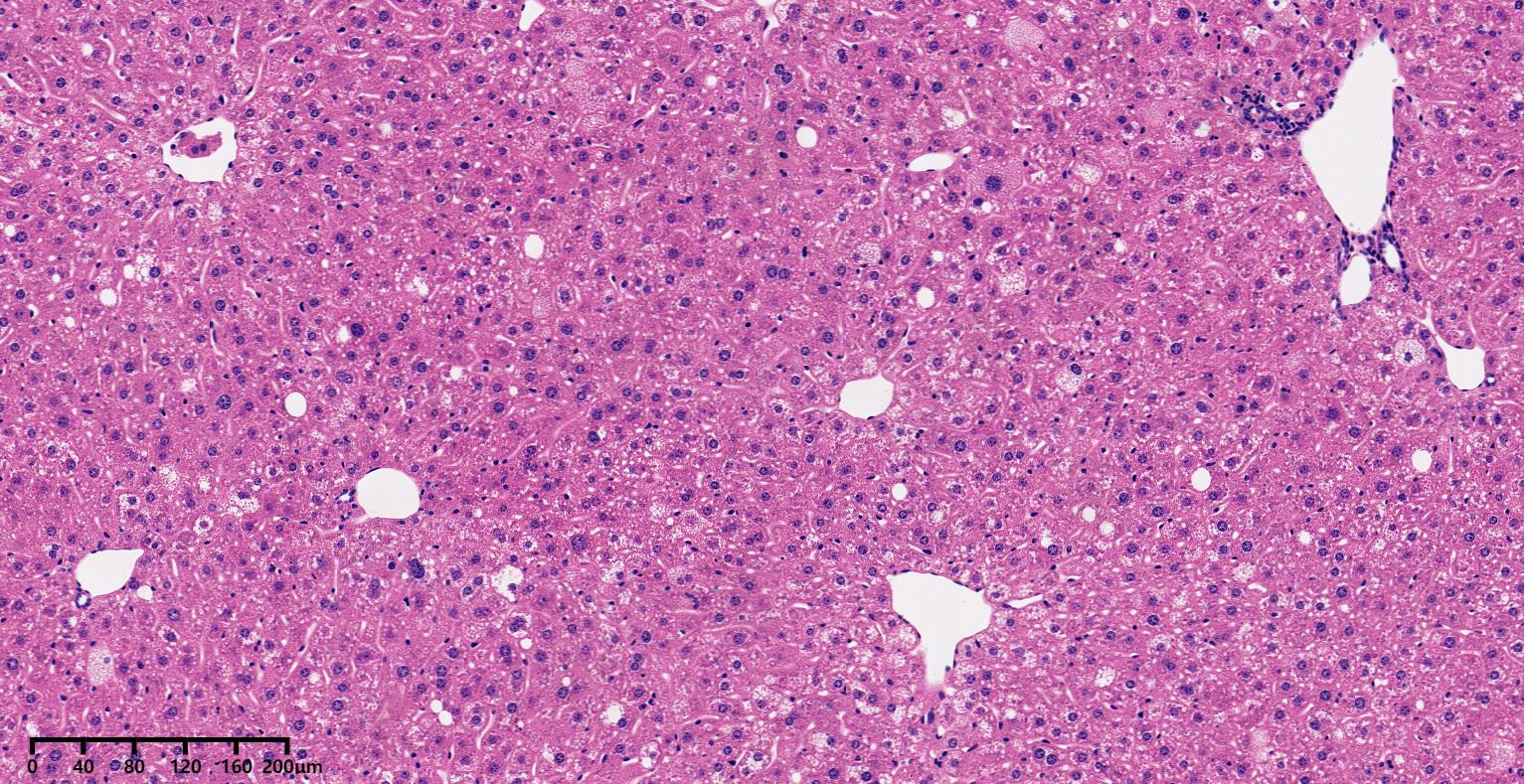

Supplement: Figure S4 [file peerj-11-16407-s007.zip › Figure 4/Liver HE/T2DM+CUMS liver_10.00X_20230227115800.jpg]

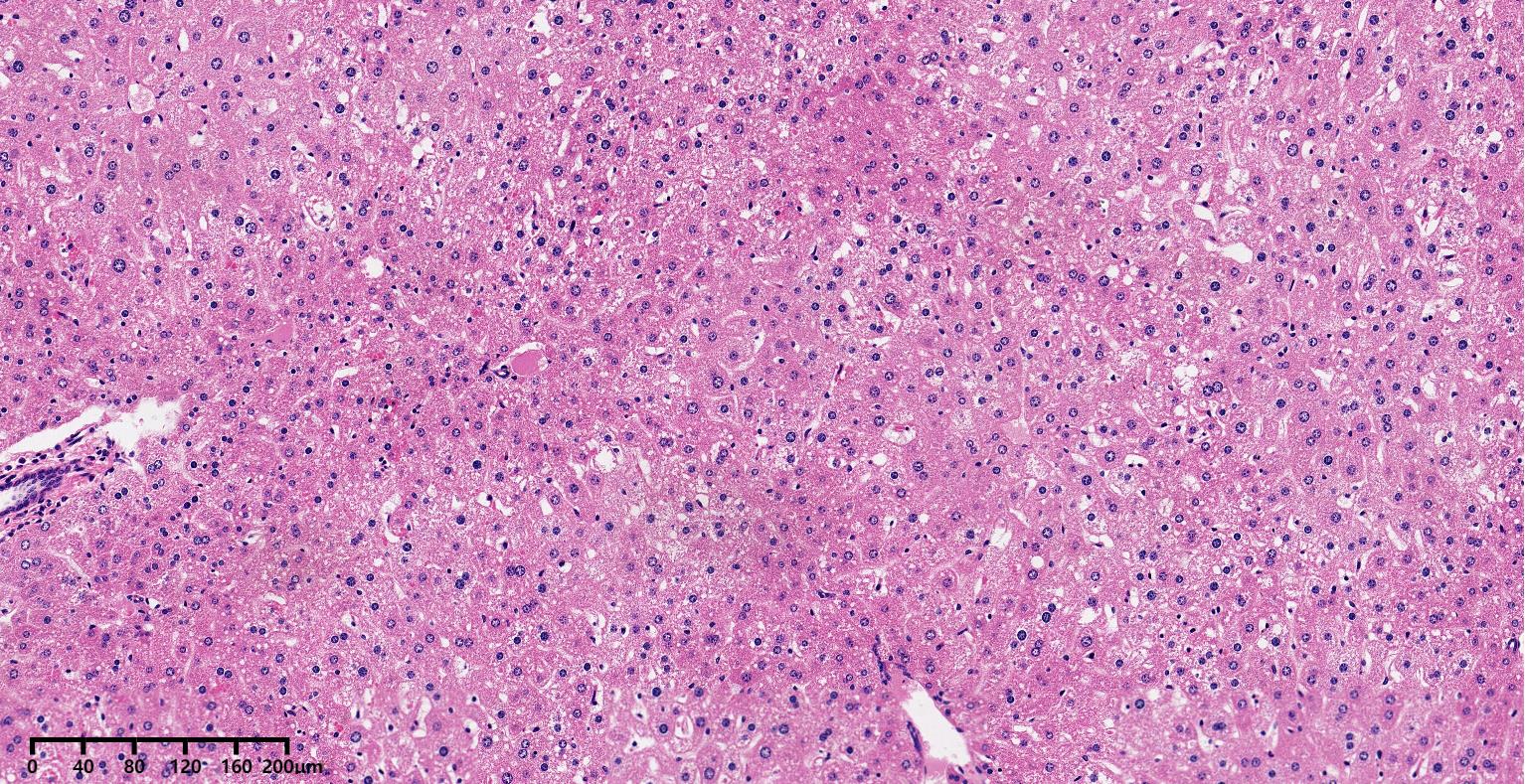

Supplement: Figure S4 [file peerj-11-16407-s007.zip › Figure 4/Liver HE/T2DM+CUMS liver_10.00X_20230227115828.jpg]

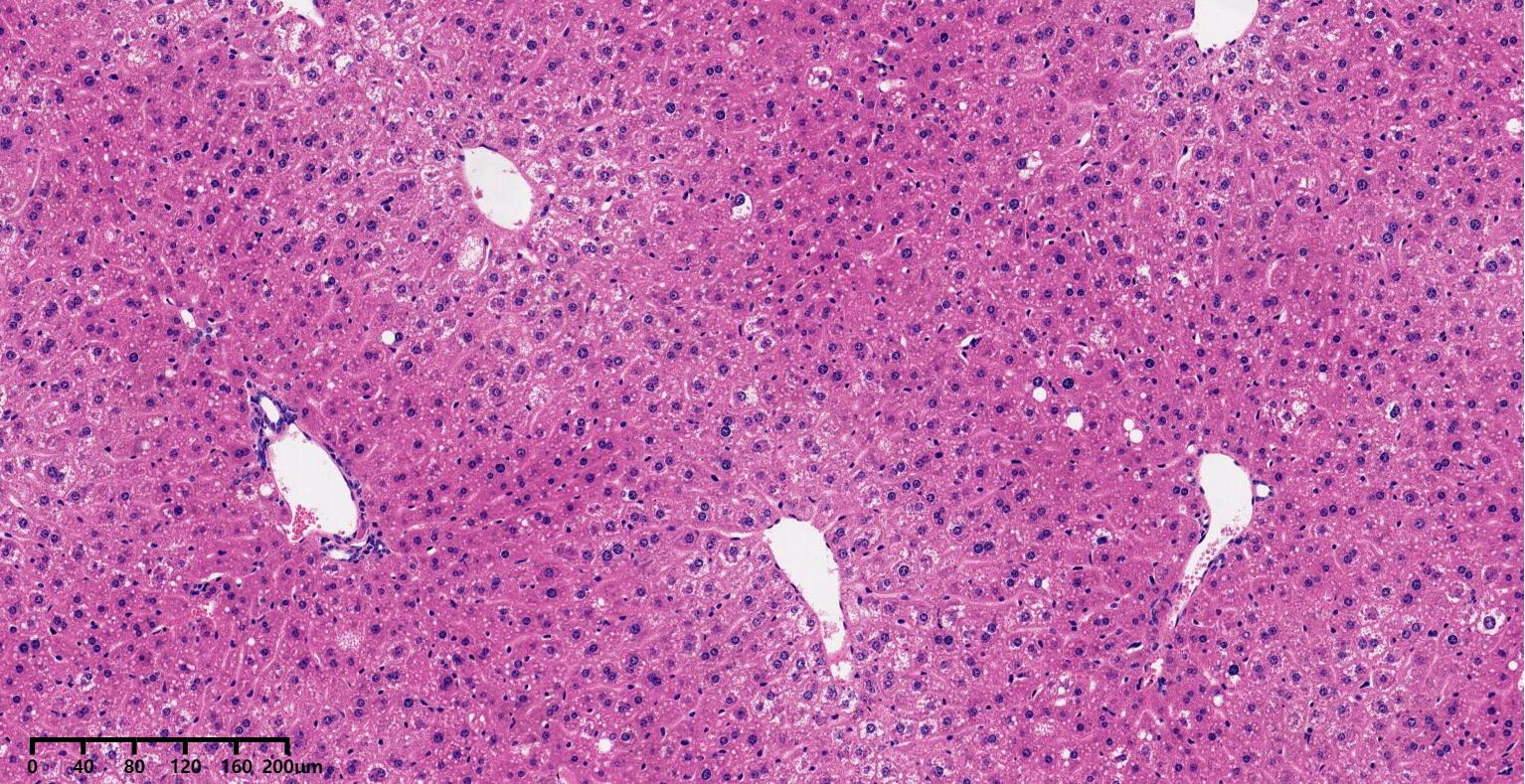

Supplement: Figure S4 [file peerj-11-16407-s007.zip › Figure 4/Liver HE/T2DM+CUMS liver_10.00X_20230227115855.jpg]

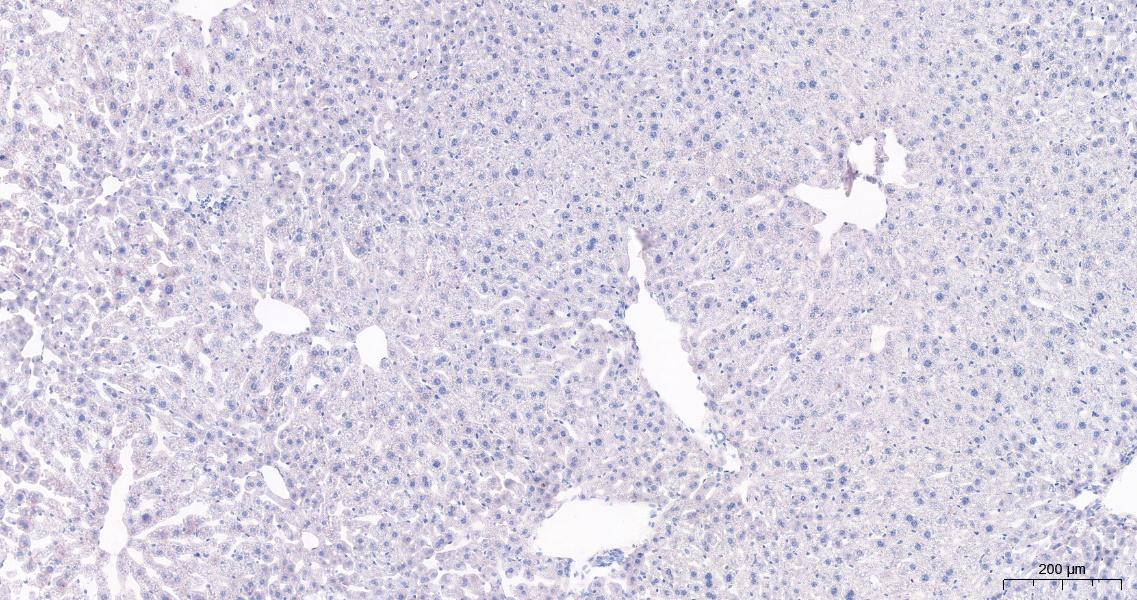

Supplement: Figure S4 [file peerj-11-16407-s007.zip › Figure 4/Liver oil red O/CUMS 1liver_10.0x.jpg]

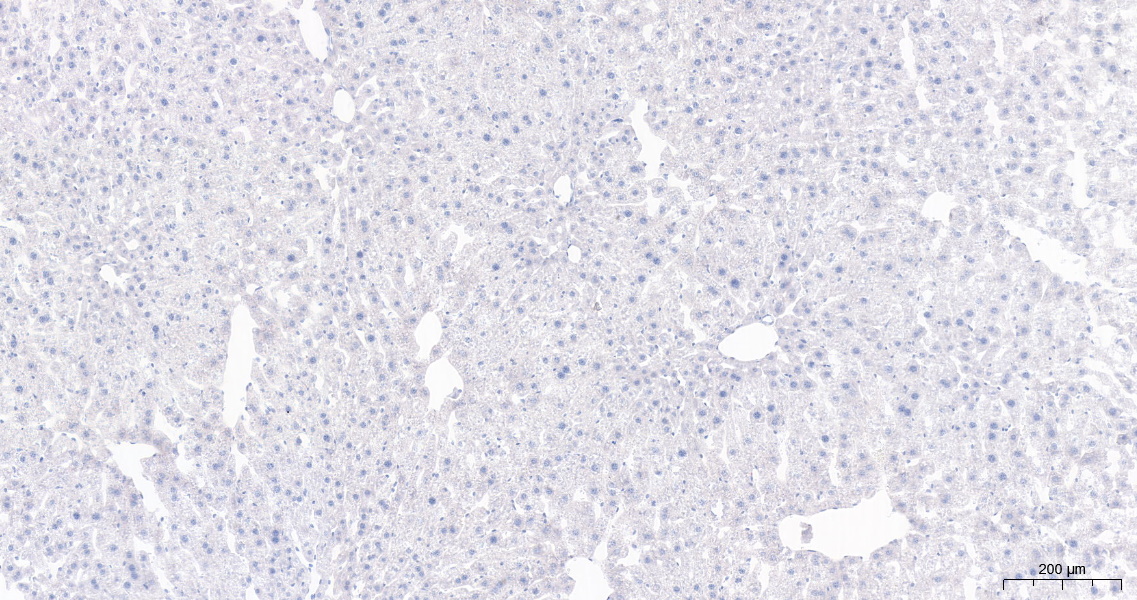

Supplement: Figure S4 [file peerj-11-16407-s007.zip › Figure 4/Liver oil red O/CUMS 2liver_10.0x.jpg]

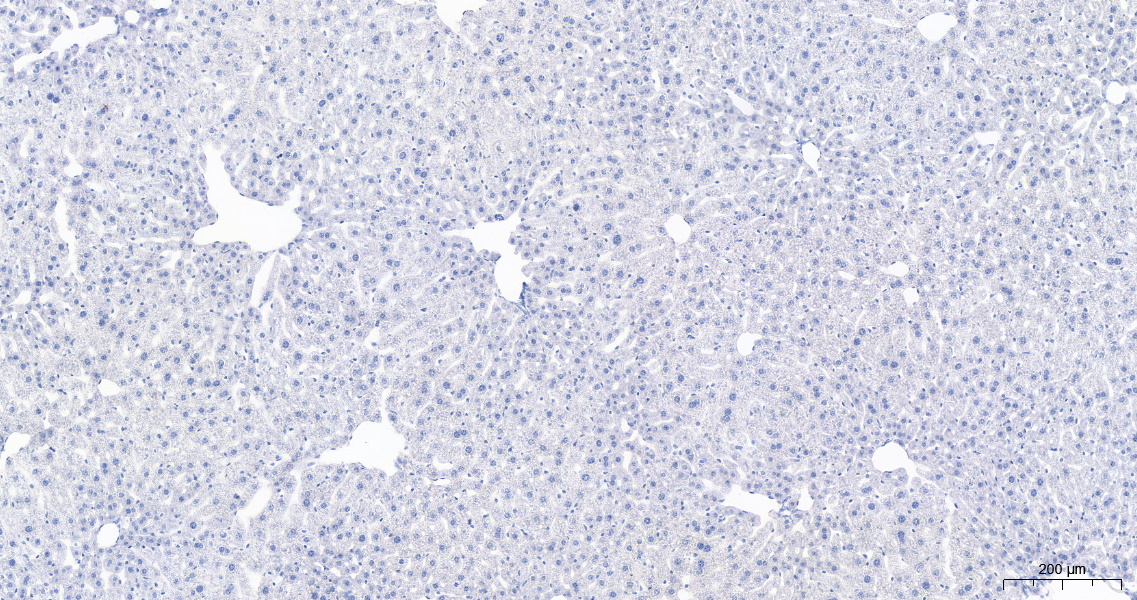

Supplement: Figure S4 [file peerj-11-16407-s007.zip › Figure 4/Liver oil red O/CUMS 3liver_10.0x.jpg]

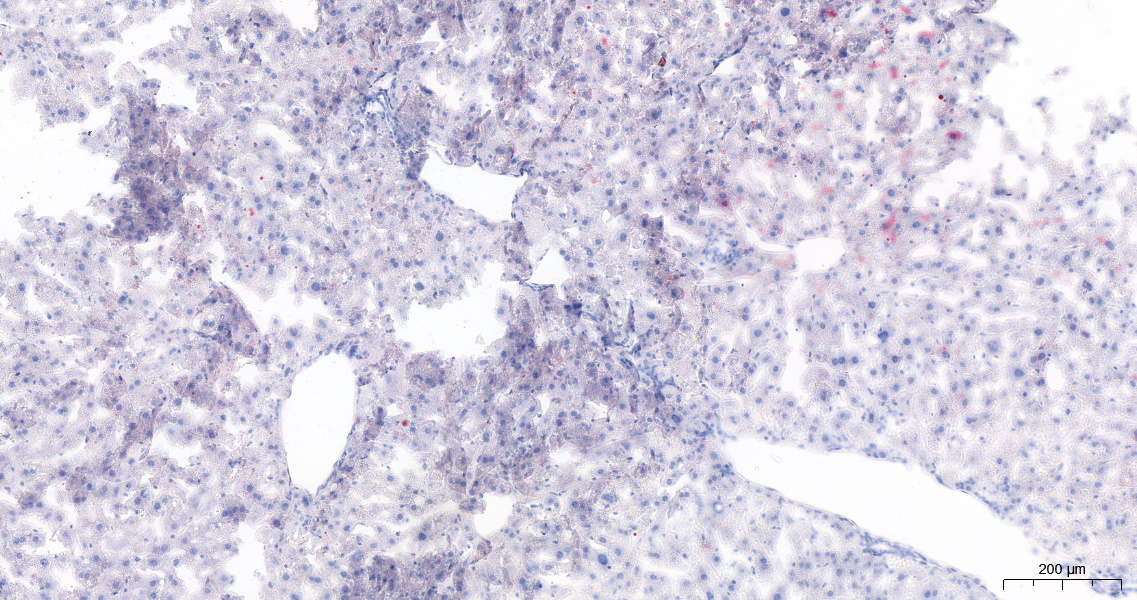

Supplement: Figure S4 [file peerj-11-16407-s007.zip › Figure 4/Liver oil red O/CUMS 4liver_10.0x.jpg]

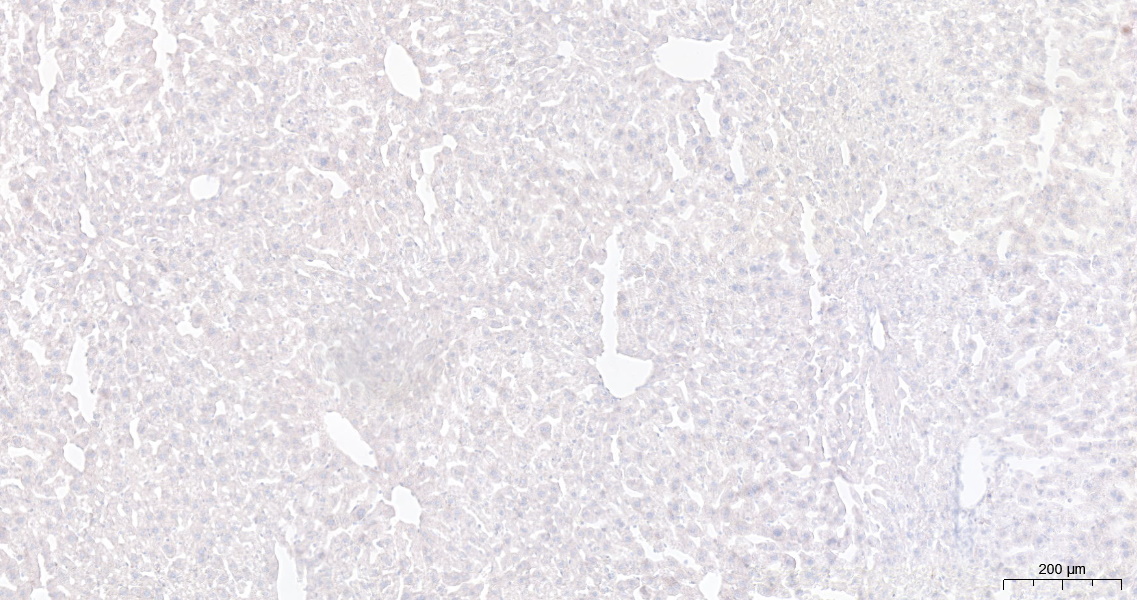

Supplement: Figure S4 [file peerj-11-16407-s007.zip › Figure 4/Liver oil red O/Control 1liver_10.0x.jpg]

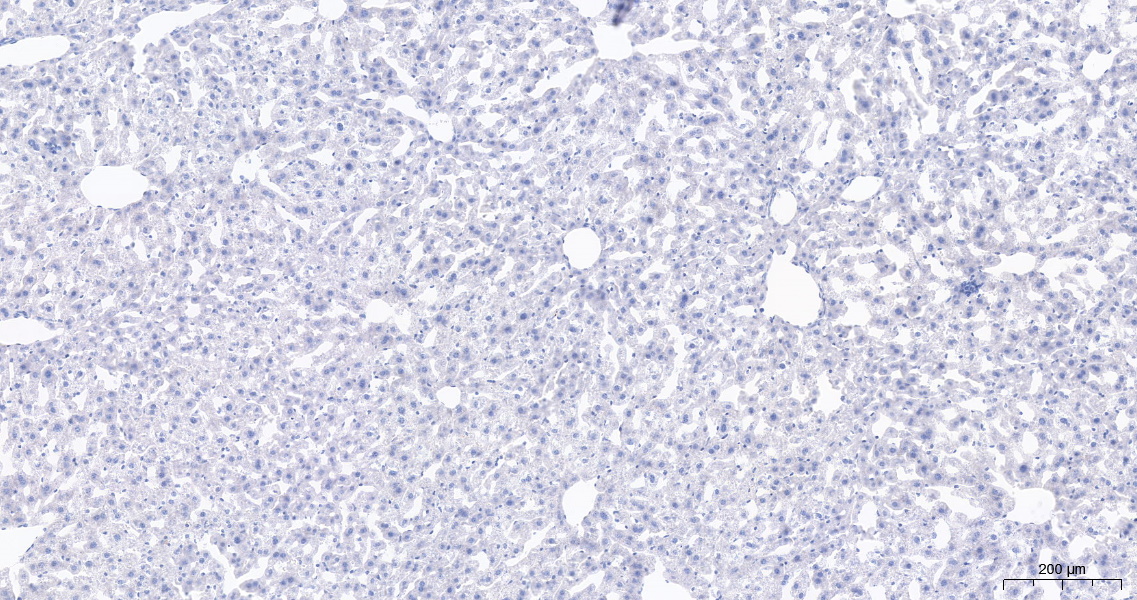

Supplement: Figure S4 [file peerj-11-16407-s007.zip › Figure 4/Liver oil red O/Control 2liver_10.0x.jpg]

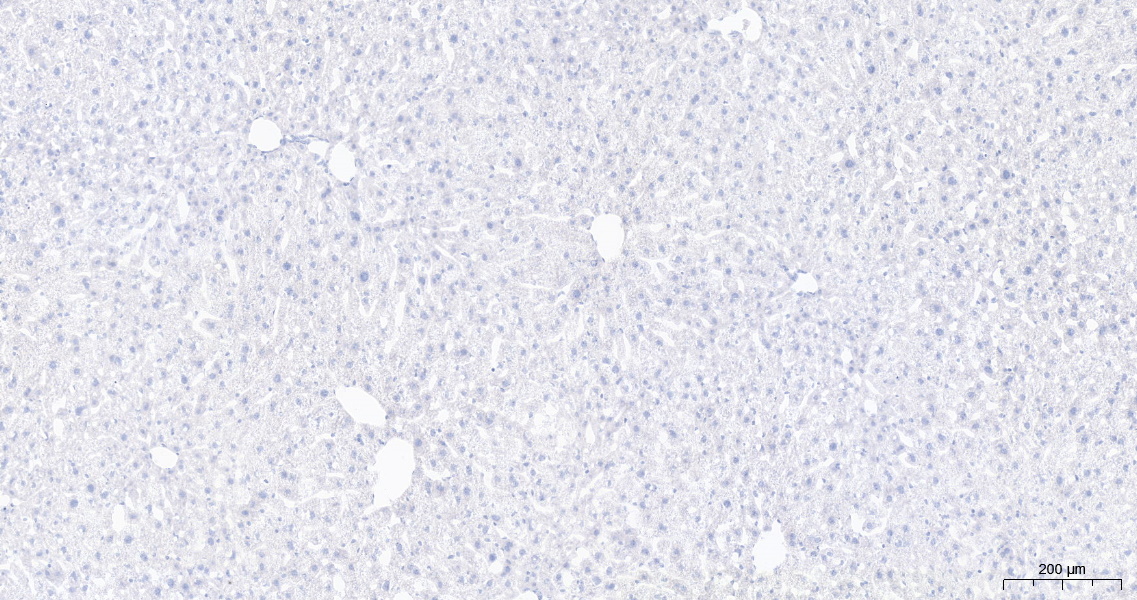

Supplement: Figure S4 [file peerj-11-16407-s007.zip › Figure 4/Liver oil red O/Control 3liver_10.0x.jpg]

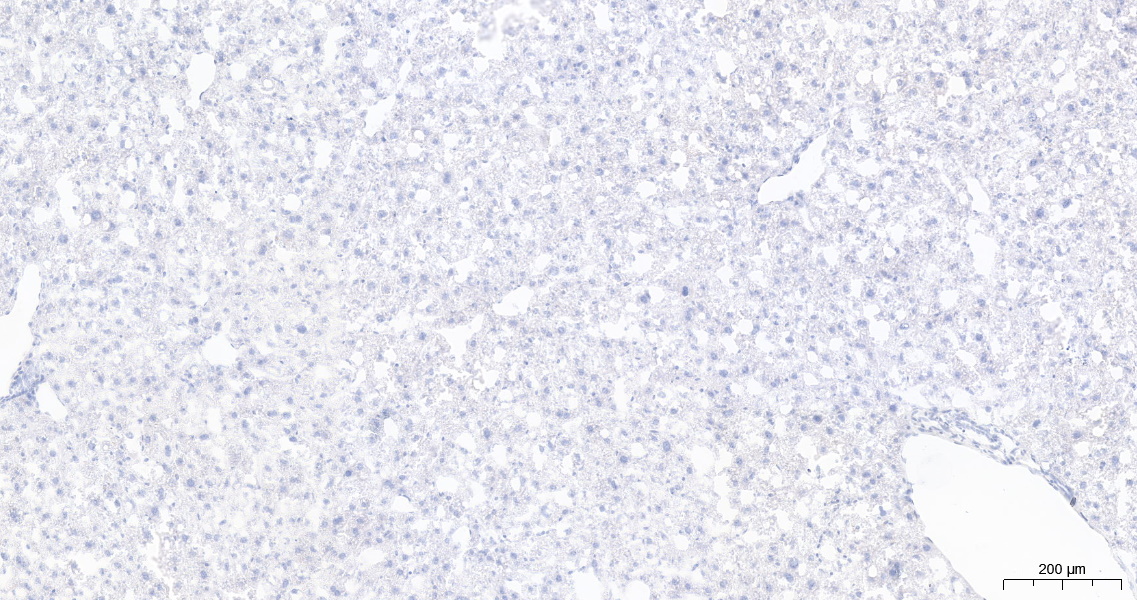

Supplement: Figure S4 [file peerj-11-16407-s007.zip › Figure 4/Liver oil red O/T2DM 1liver_10.0x.jpg]

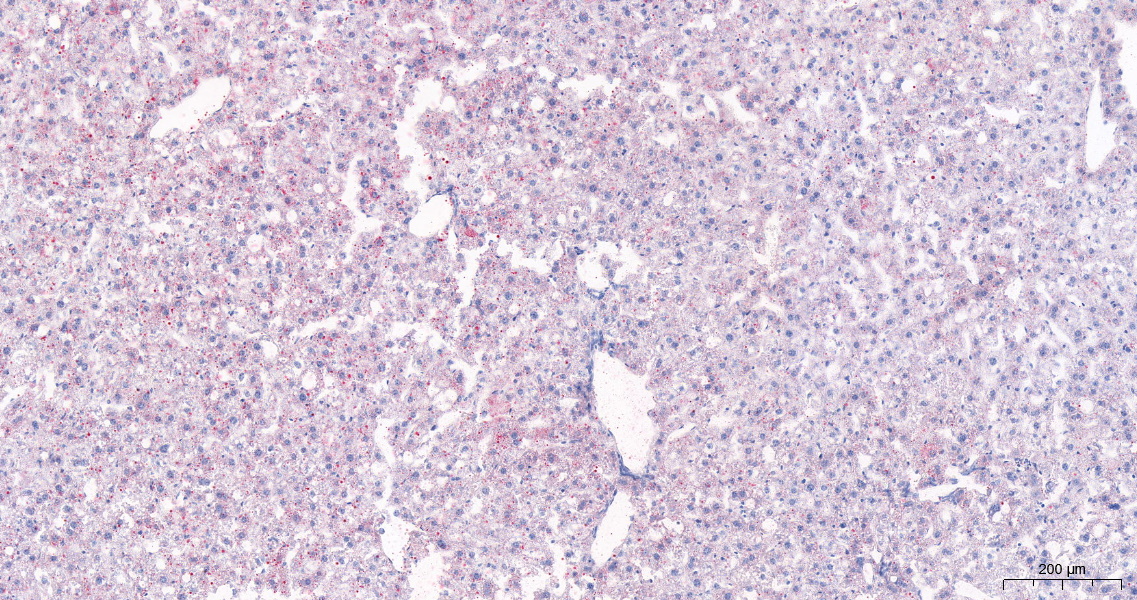

Supplement: Figure S4 [file peerj-11-16407-s007.zip › Figure 4/Liver oil red O/T2DM 2liver_10.0x.jpg]

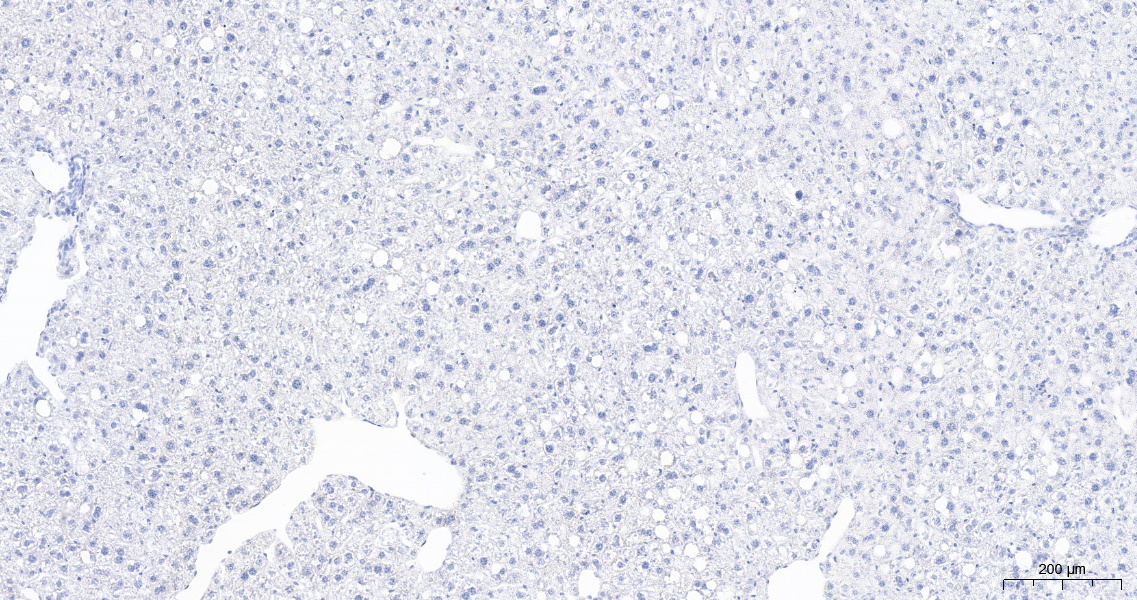

Supplement: Figure S4 [file peerj-11-16407-s007.zip › Figure 4/Liver oil red O/T2DM 3liver_10.0x.jpg]

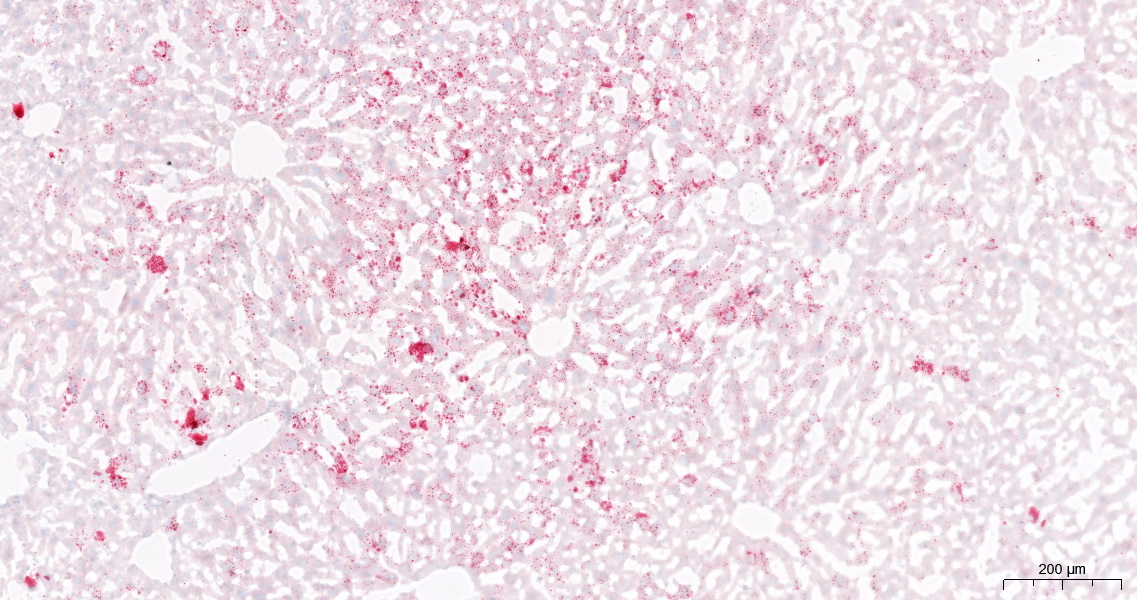

Supplement: Figure S4 [file peerj-11-16407-s007.zip › Figure 4/Liver oil red O/T2DM+CUMS 1liver_10.0x.jpg]

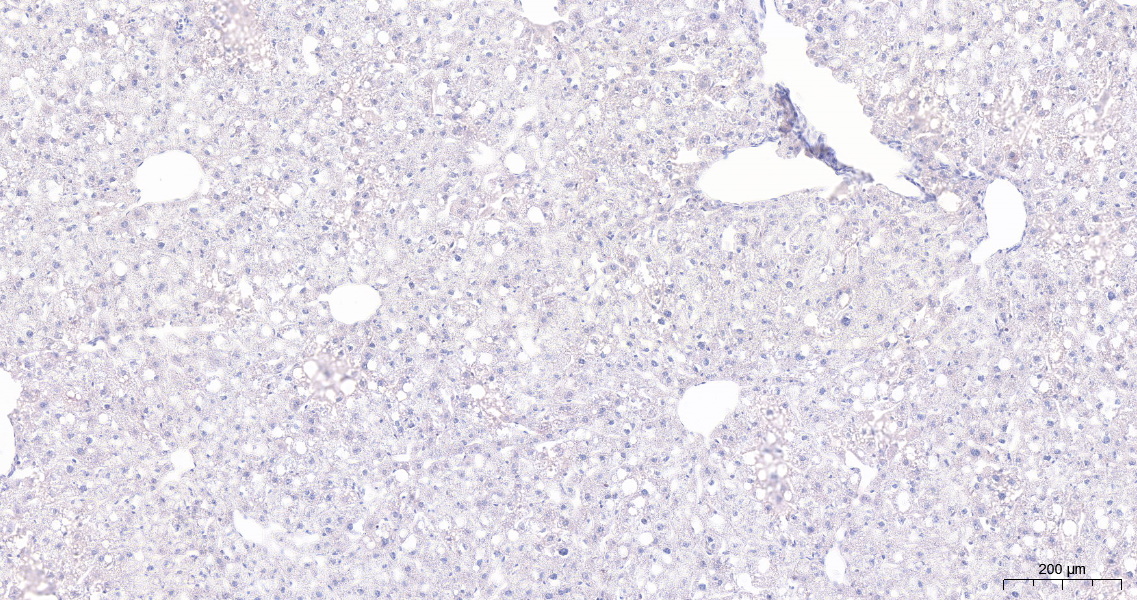

Supplement: Figure S4 [file peerj-11-16407-s007.zip › Figure 4/Liver oil red O/T2DM+CUMS 2liver_10.0x.jpg]

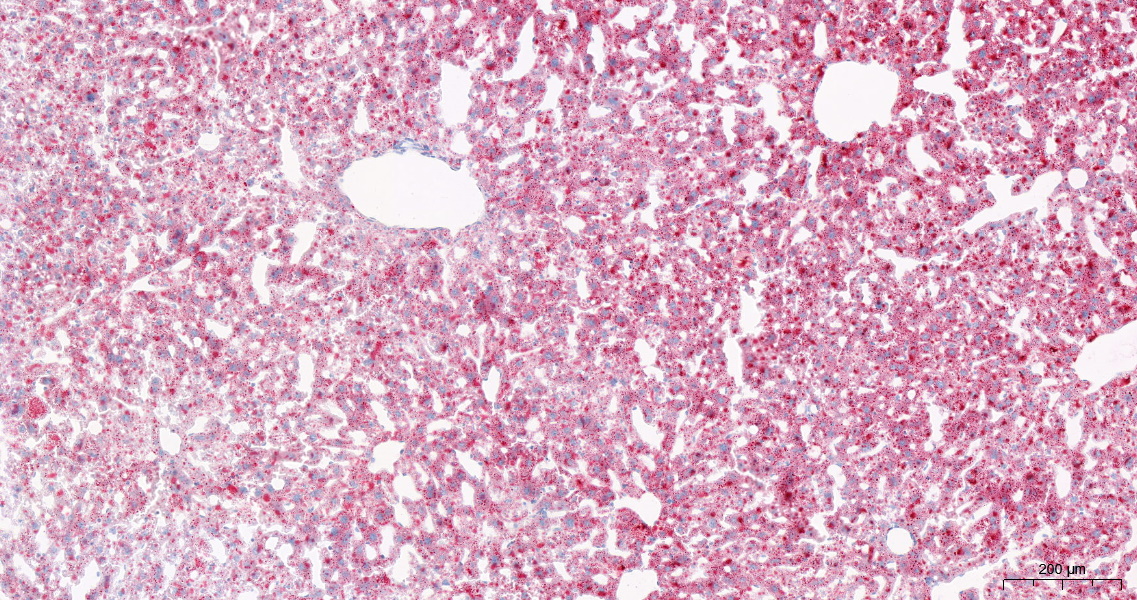

Supplement: Figure S4 [file peerj-11-16407-s007.zip › Figure 4/Liver oil red O/T2DM+CUMS 3liver_10.0x.jpg]

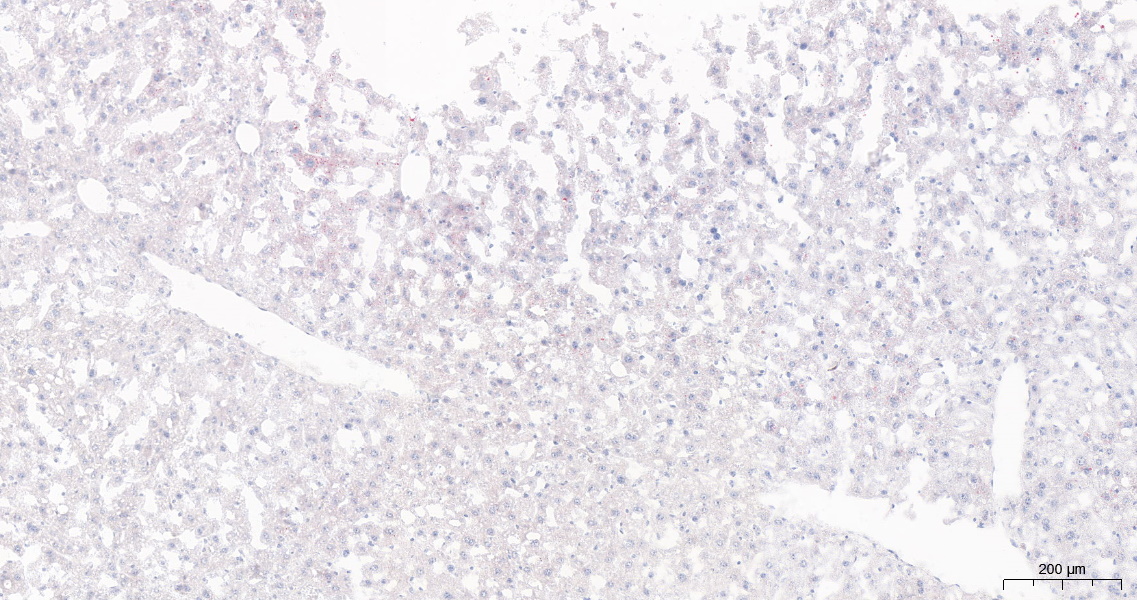

Supplement: Figure S4 [file peerj-11-16407-s007.zip › Figure 4/Liver oil red O/T2DM+CUMS 5liver_10.0x.jpg]

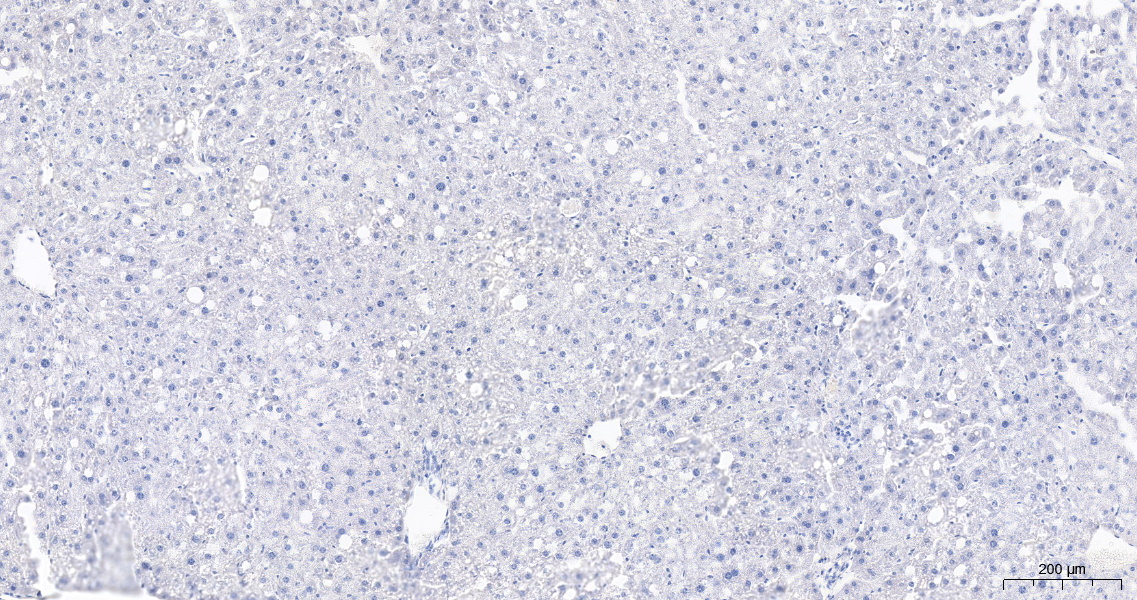

Supplement: Figure S4 [file peerj-11-16407-s007.zip › Figure 4/Liver oil red O/T2DM+CUMS 7liver_10.0x.jpg]

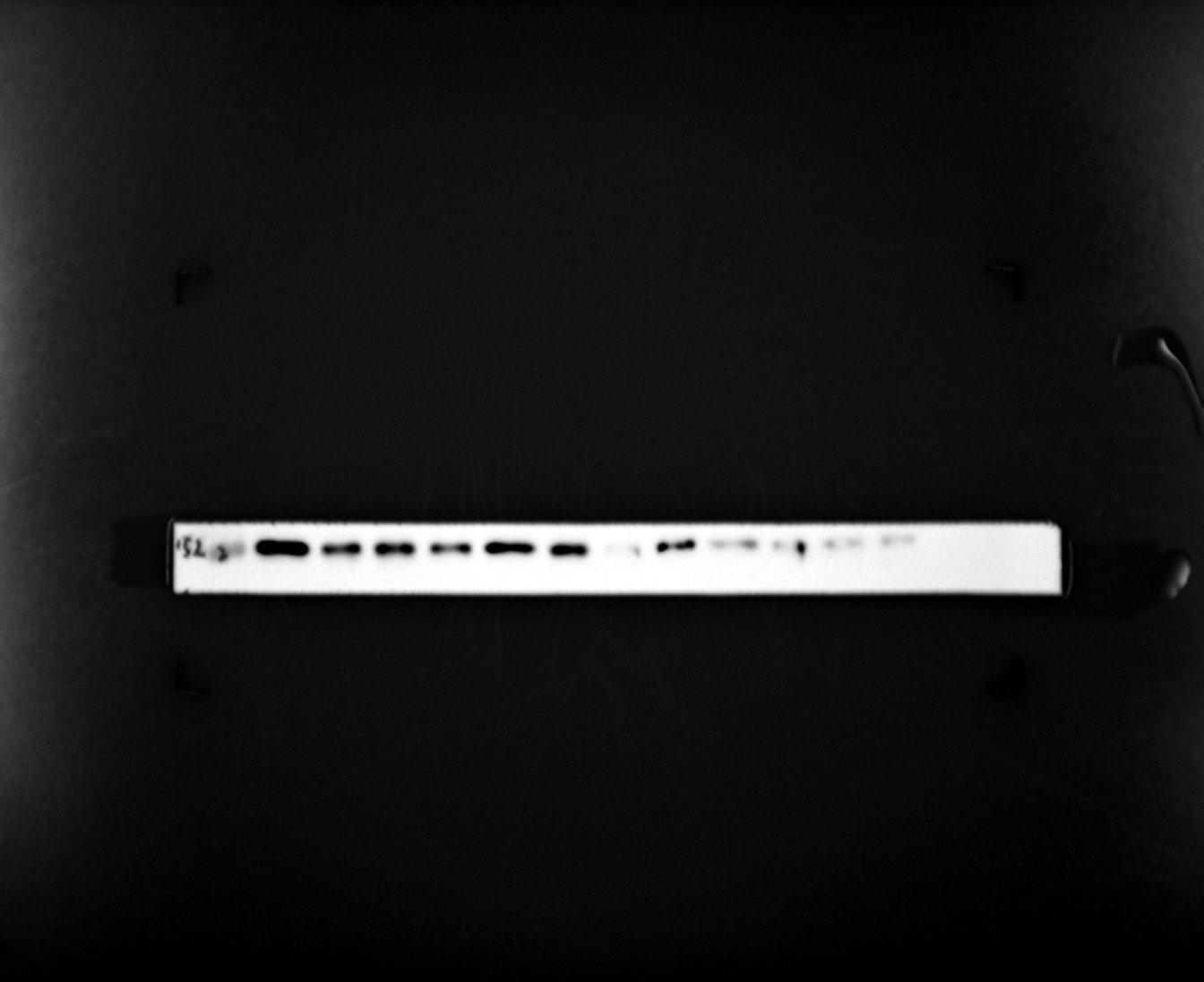

Supplement: Figure S5 [file peerj-11-16407-s008.zip › Figure 5/FXR&FGF15 protein/1/12.5%-ILEUM-FGF15-2-1.Tif]

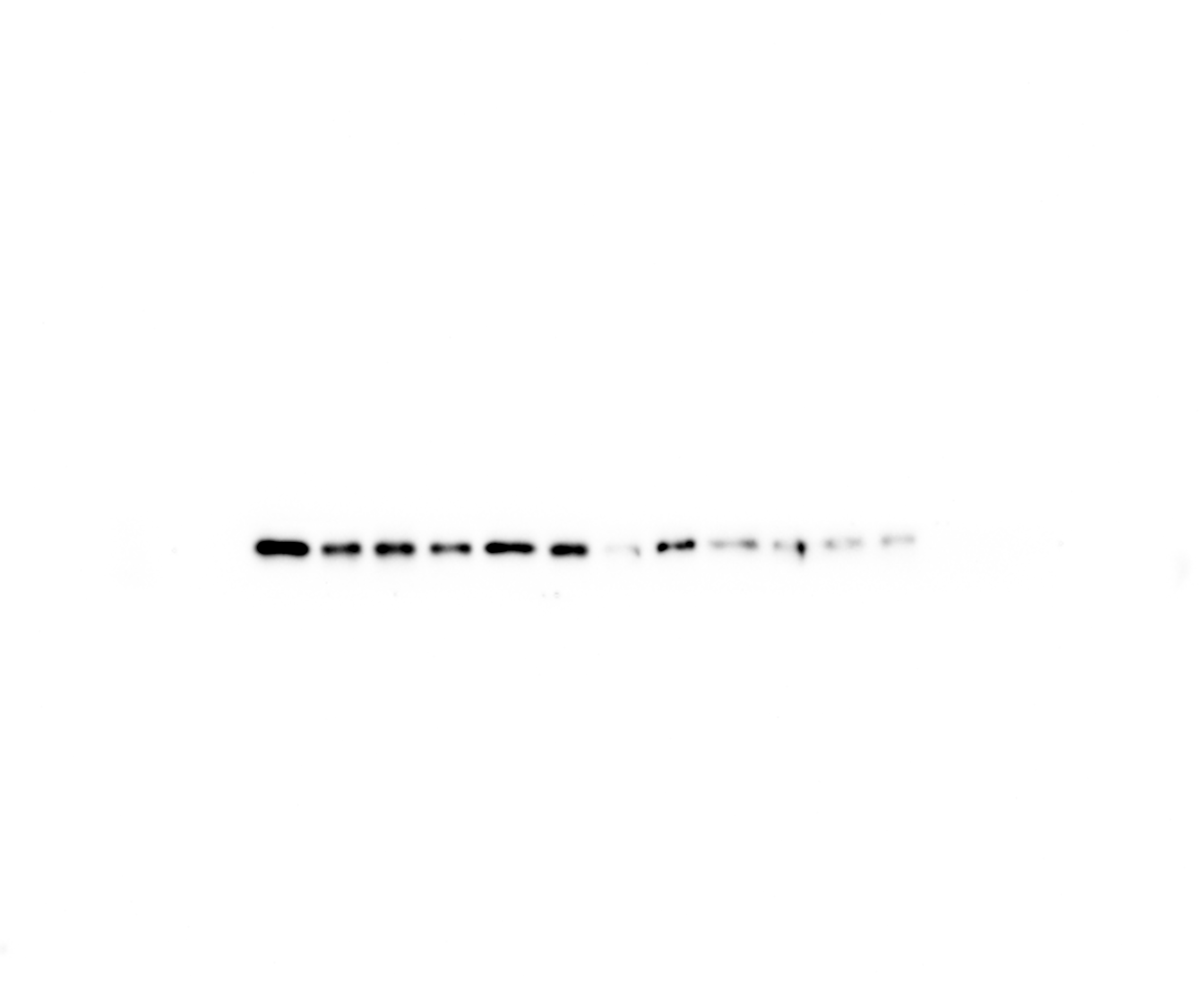

Supplement: Figure S5 [file peerj-11-16407-s008.zip › Figure 5/FXR&FGF15 protein/1/12.5%-ILEUM-FGF15-2.Tif]

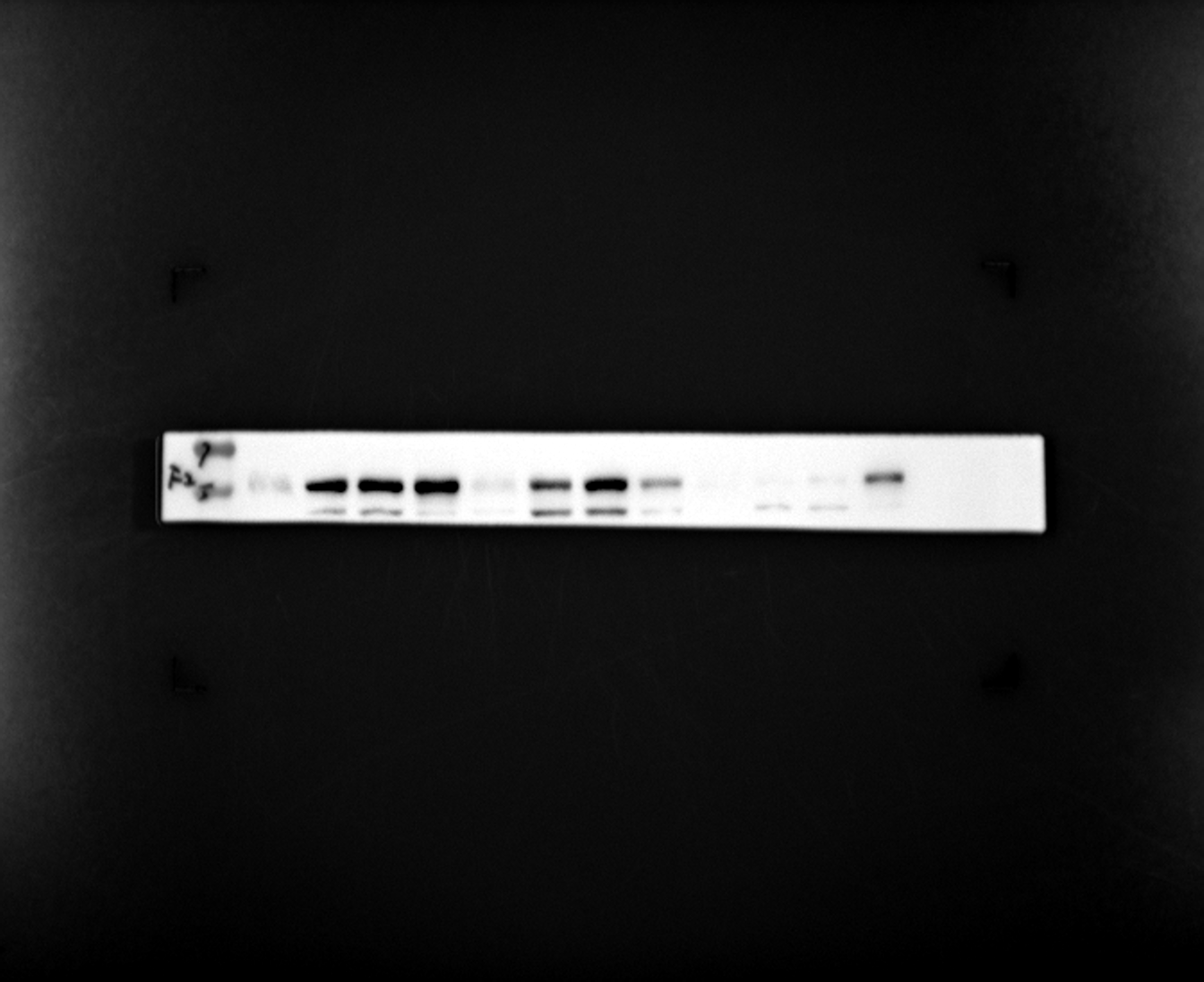

Supplement: Figure S5 [file peerj-11-16407-s008.zip › Figure 5/FXR&FGF15 protein/1/12.5%-ILEUM-FXR-2-1.Tif]

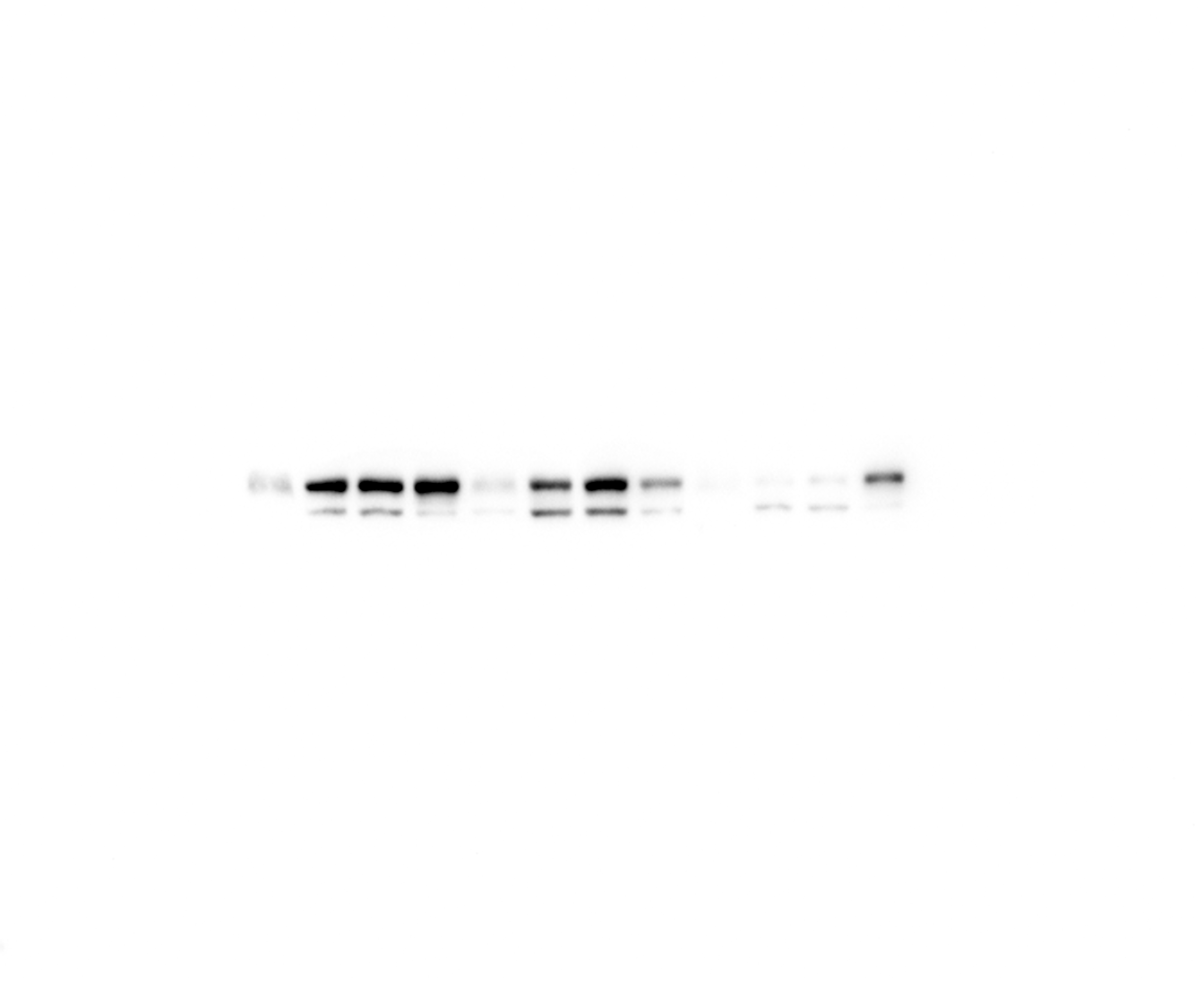

Supplement: Figure S5 [file peerj-11-16407-s008.zip › Figure 5/FXR&FGF15 protein/1/12.5%-ILEUM-FXR-2.Tif]

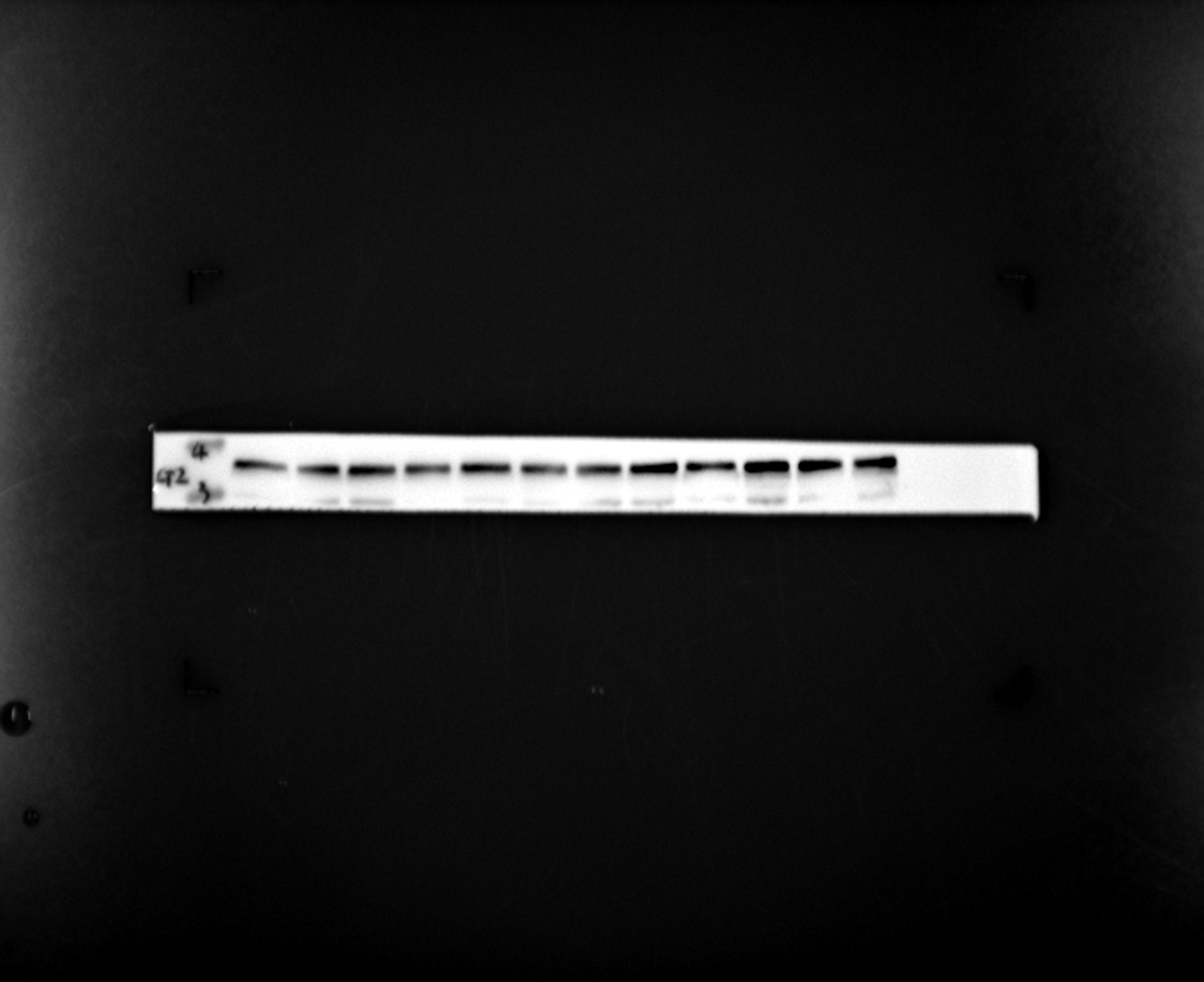

Supplement: Figure S5 [file peerj-11-16407-s008.zip › Figure 5/FXR&FGF15 protein/1/12.5%-ILEUM-GAPDH-2-1.Tif]

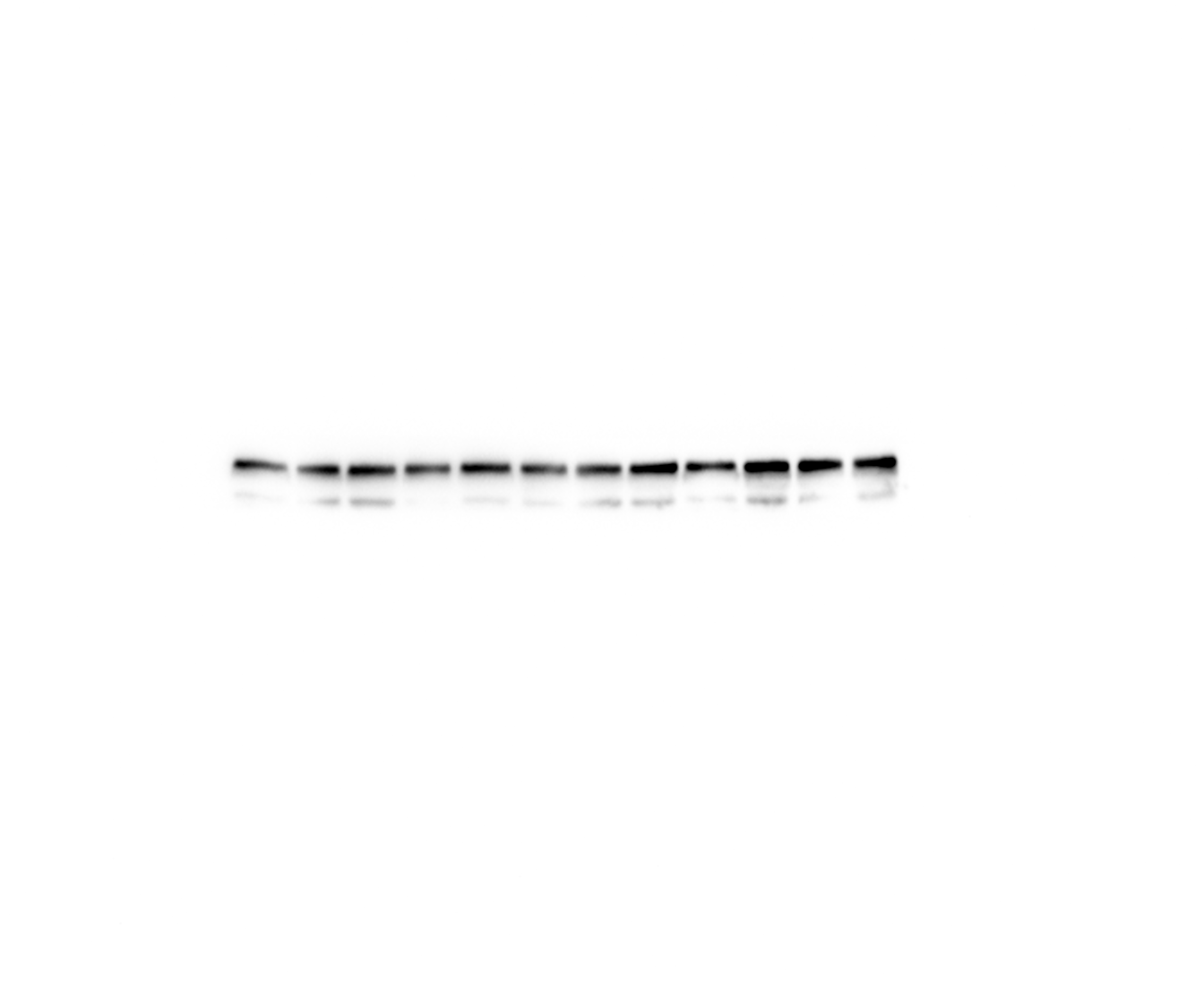

Supplement: Figure S5 [file peerj-11-16407-s008.zip › Figure 5/FXR&FGF15 protein/1/12.5%-ILEUM-GAPDH-2.Tif]

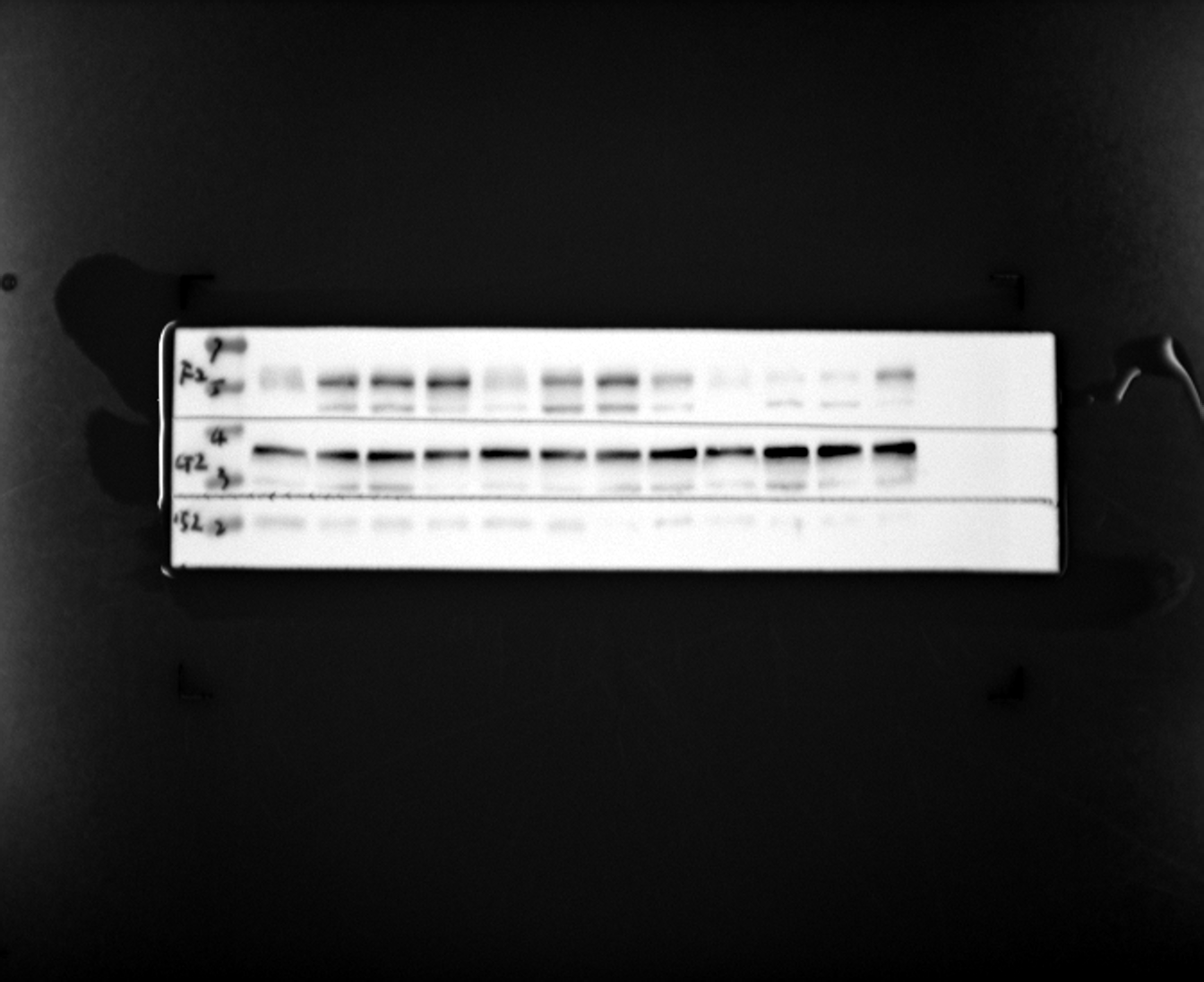

Supplement: Figure S5 [file peerj-11-16407-s008.zip › Figure 5/FXR&FGF15 protein/1/12.5%-ILEUM-GAPDH-FXR-FGF15-2-1.Tif]

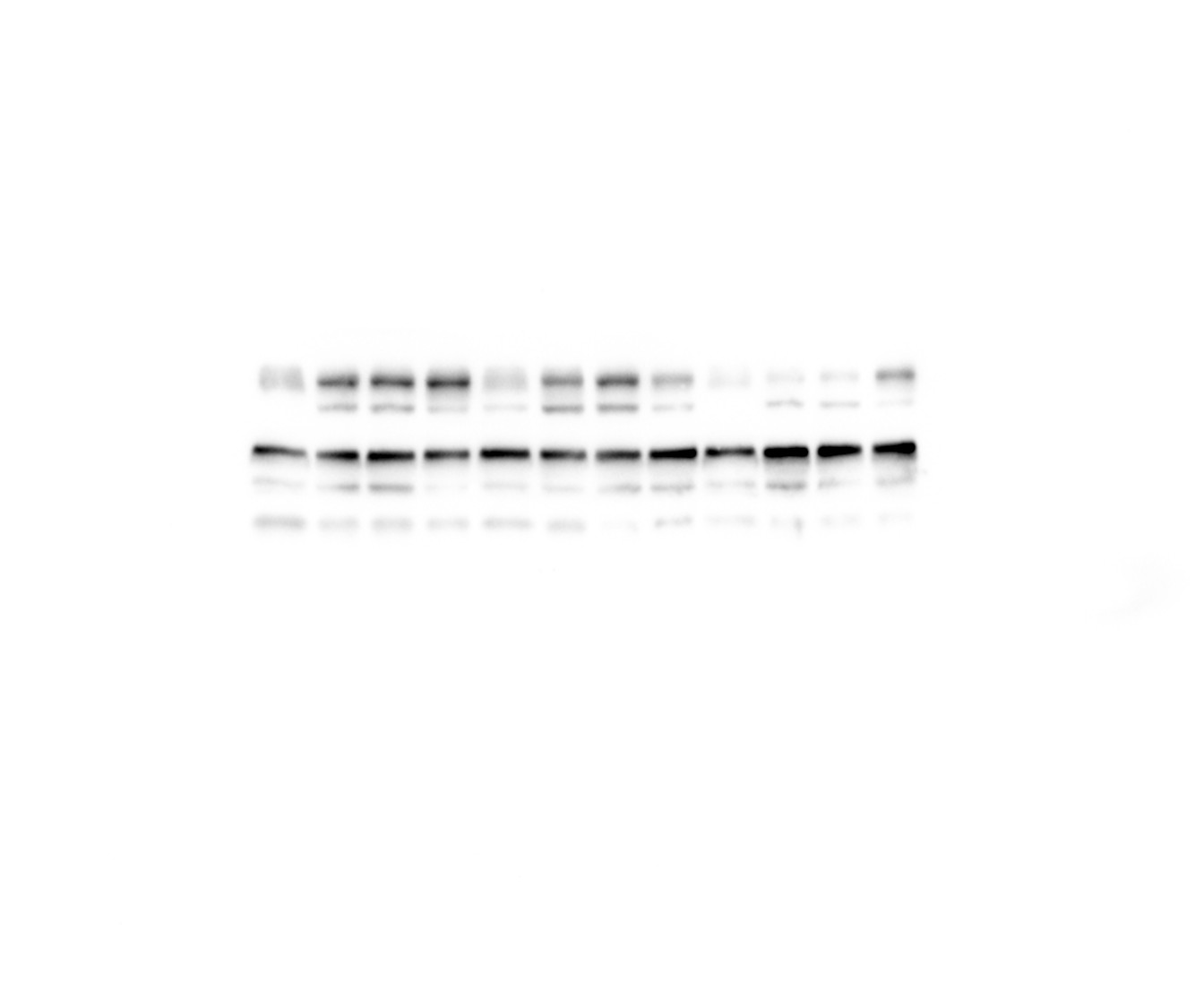

Supplement: Figure S5 [file peerj-11-16407-s008.zip › Figure 5/FXR&FGF15 protein/1/12.5%-ILEUM-GAPDH-FXR-FGF15-2.Tif]

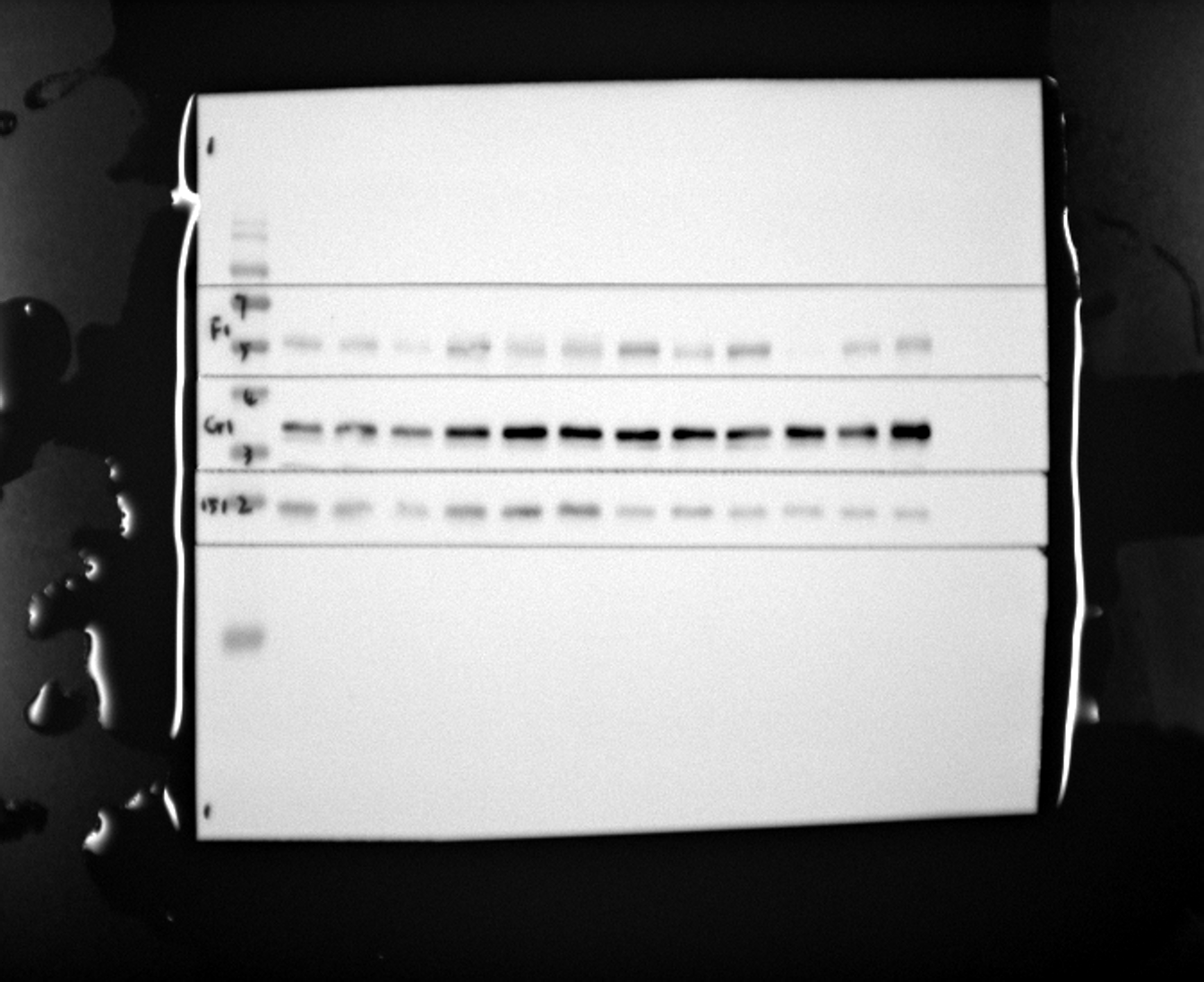

Supplement: Figure S5 [file peerj-11-16407-s008.zip › Figure 5/FXR&FGF15 protein/2/12%-ILEUM-ALL-1-1.Tif]

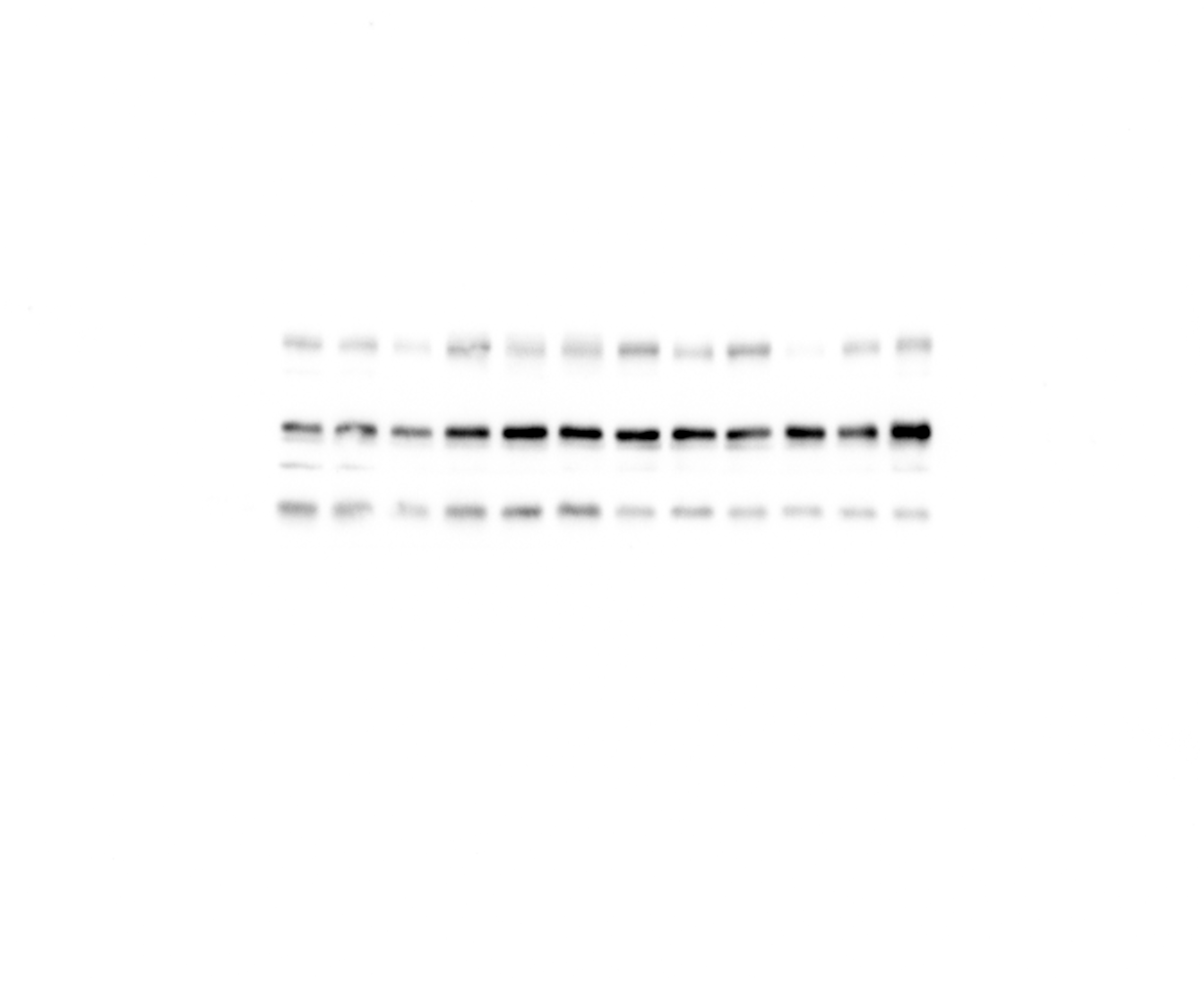

Supplement: Figure S5 [file peerj-11-16407-s008.zip › Figure 5/FXR&FGF15 protein/2/12%-ILEUM-ALL-1.Tif]

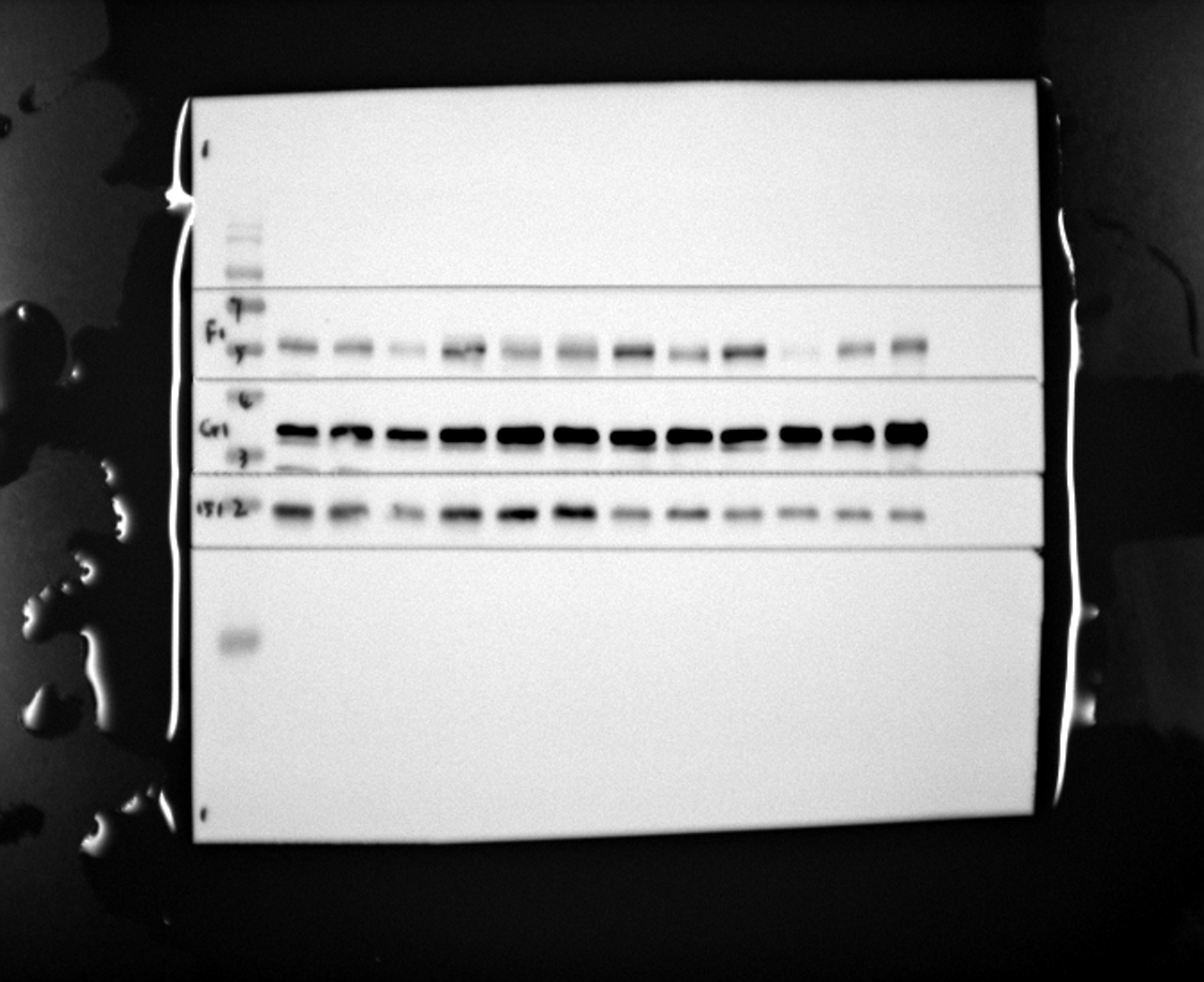

Supplement: Figure S5 [file peerj-11-16407-s008.zip › Figure 5/FXR&FGF15 protein/2/12%-ILEUM-ALL-11-1-120S.Tif]

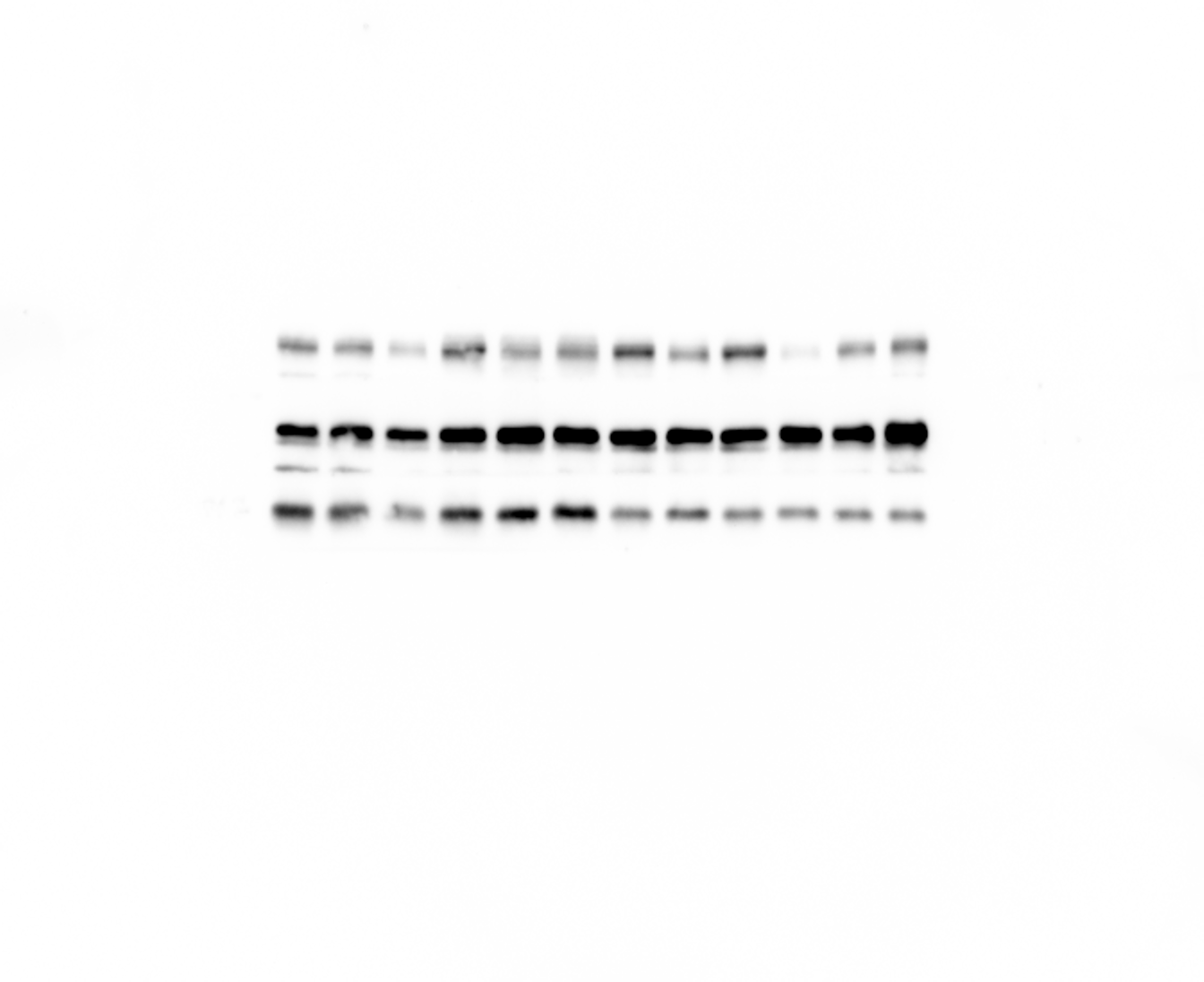

Supplement: Figure S5 [file peerj-11-16407-s008.zip › Figure 5/FXR&FGF15 protein/2/12%-ILEUM-ALL-11.Tif]

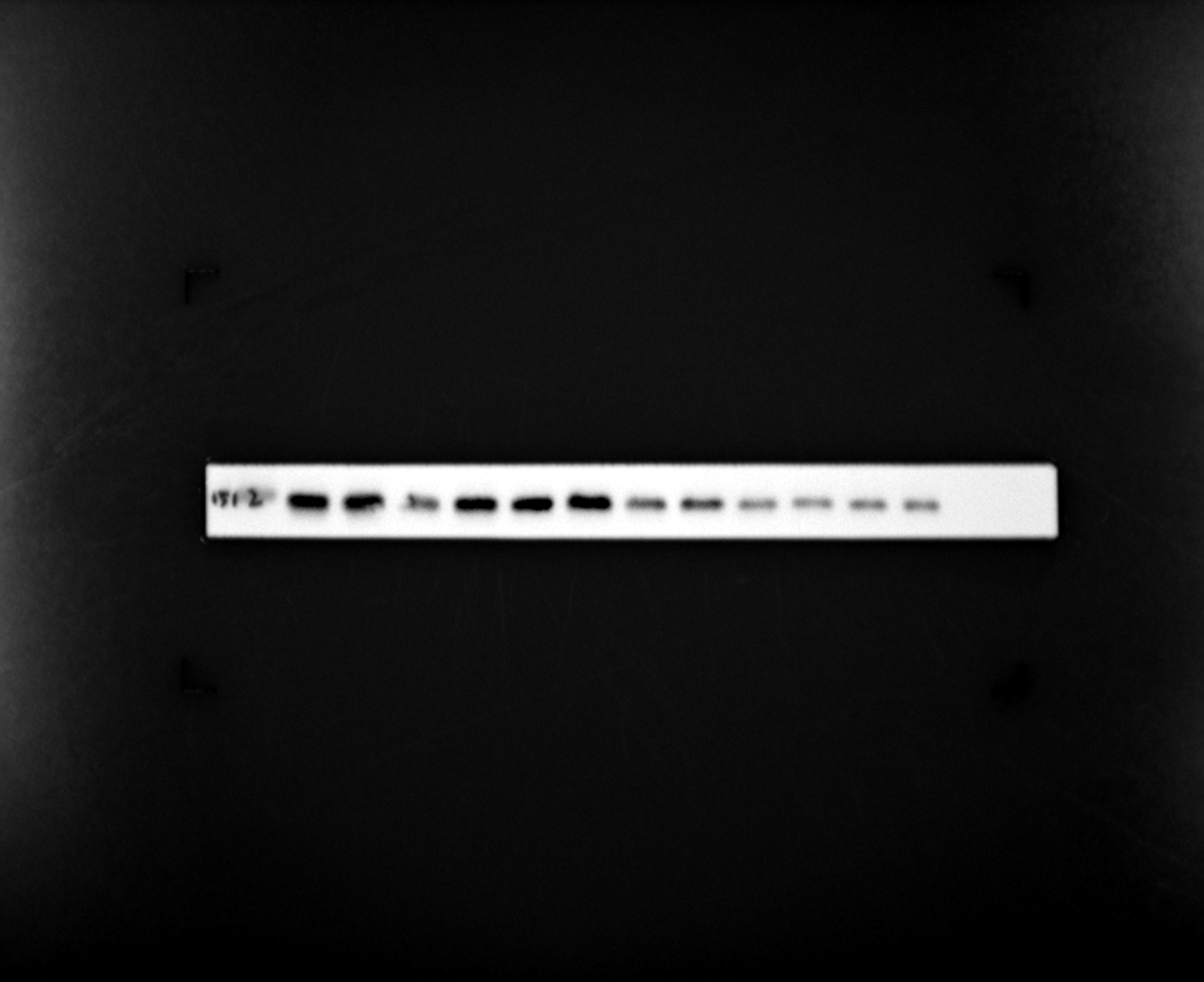

Supplement: Figure S5 [file peerj-11-16407-s008.zip › Figure 5/FXR&FGF15 protein/2/12%-ILEUM-FGF15-1-1.Tif]

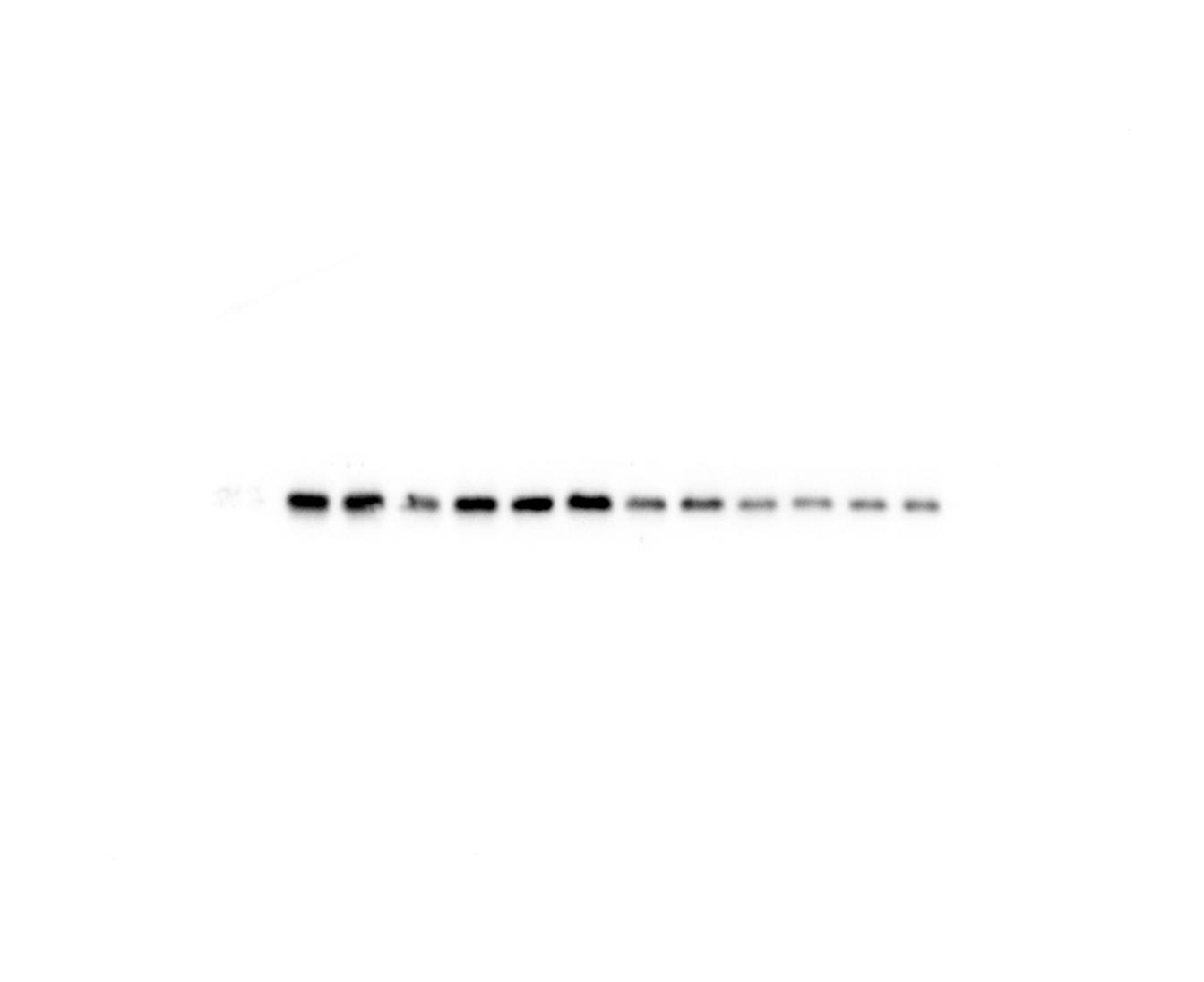

Supplement: Figure S5 [file peerj-11-16407-s008.zip › Figure 5/FXR&FGF15 protein/2/12%-ILEUM-FGF15-1.Tif]

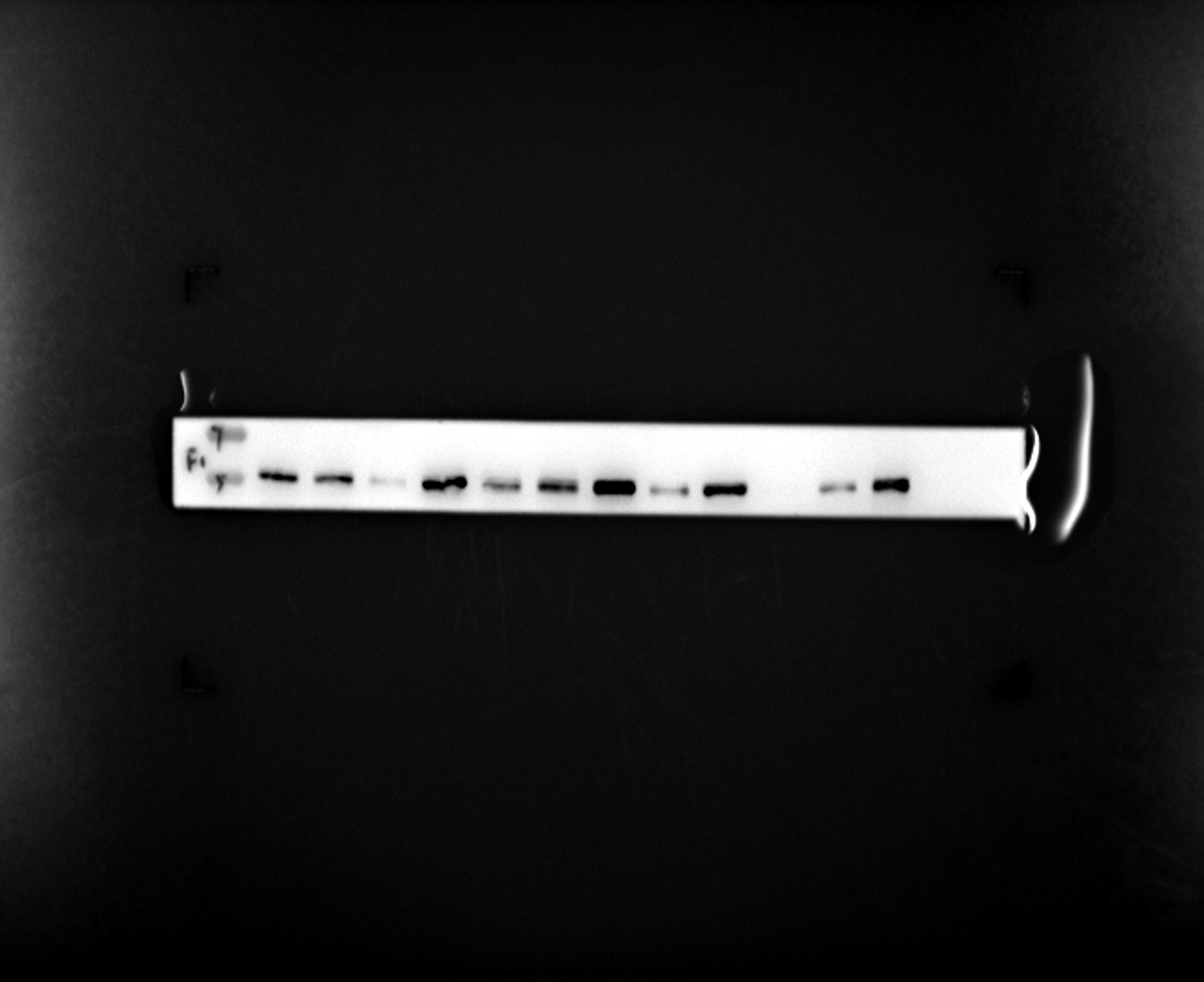

Supplement: Figure S5 [file peerj-11-16407-s008.zip › Figure 5/FXR&FGF15 protein/2/12%-ILEUM-FXR-1-1.Tif]

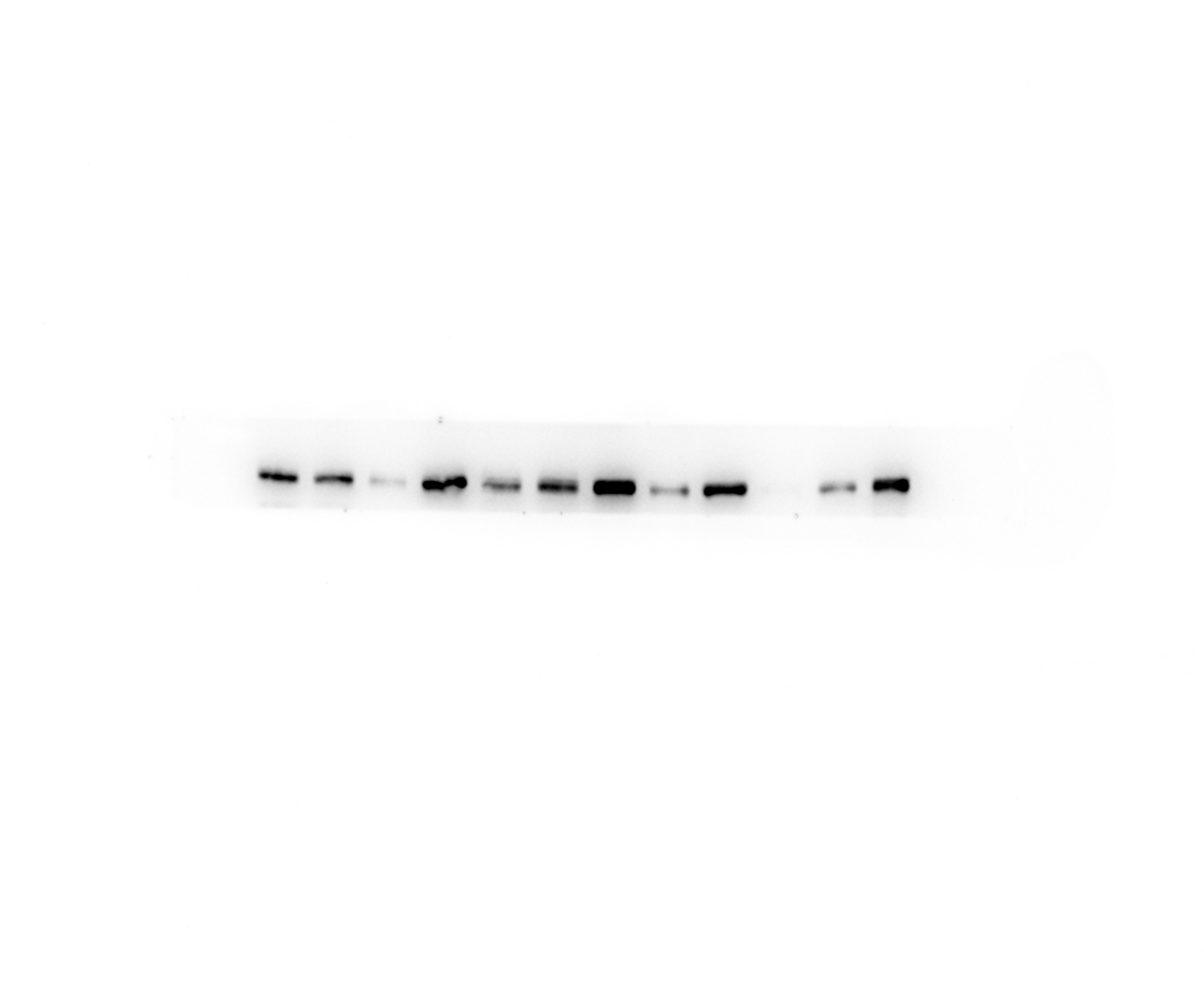

Supplement: Figure S5 [file peerj-11-16407-s008.zip › Figure 5/FXR&FGF15 protein/2/12%-ILEUM-FXR-1.Tif]

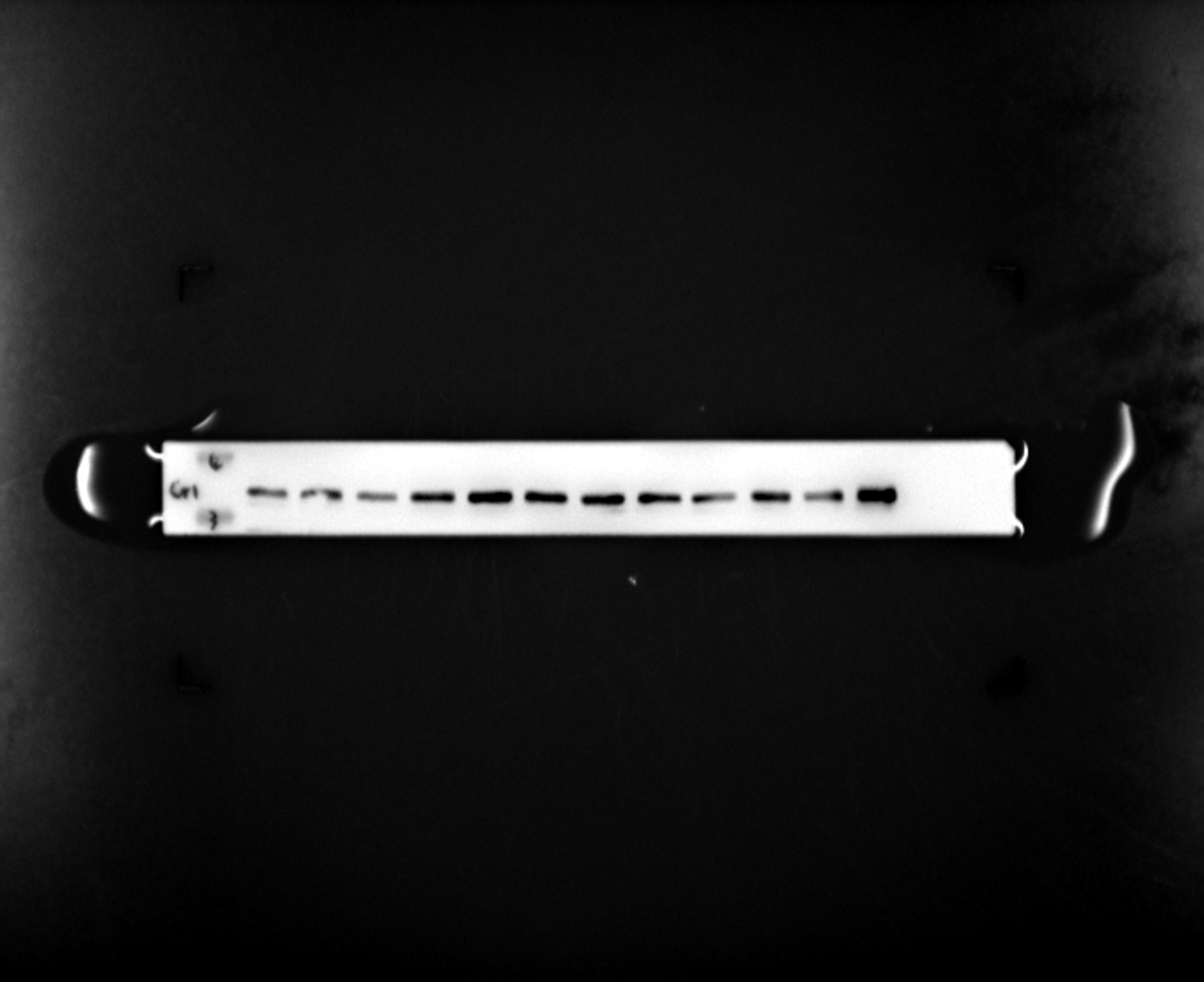

Supplement: Figure S5 [file peerj-11-16407-s008.zip › Figure 5/FXR&FGF15 protein/2/12%-ILEUM-GAPDH-1-1.Tif]

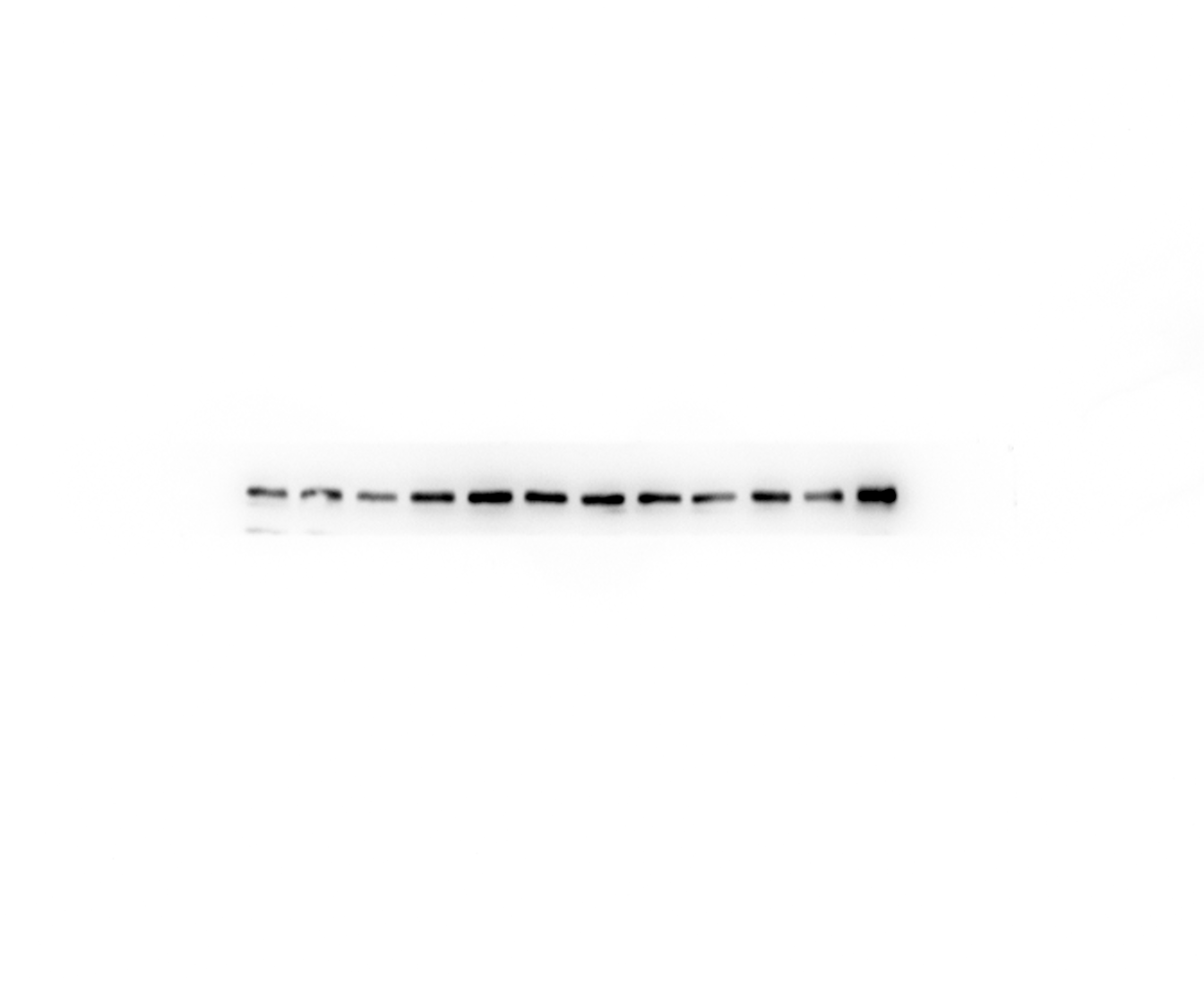

Supplement: Figure S5 [file peerj-11-16407-s008.zip › Figure 5/FXR&FGF15 protein/2/12%-ILEUM-GAPDH-1.Tif]

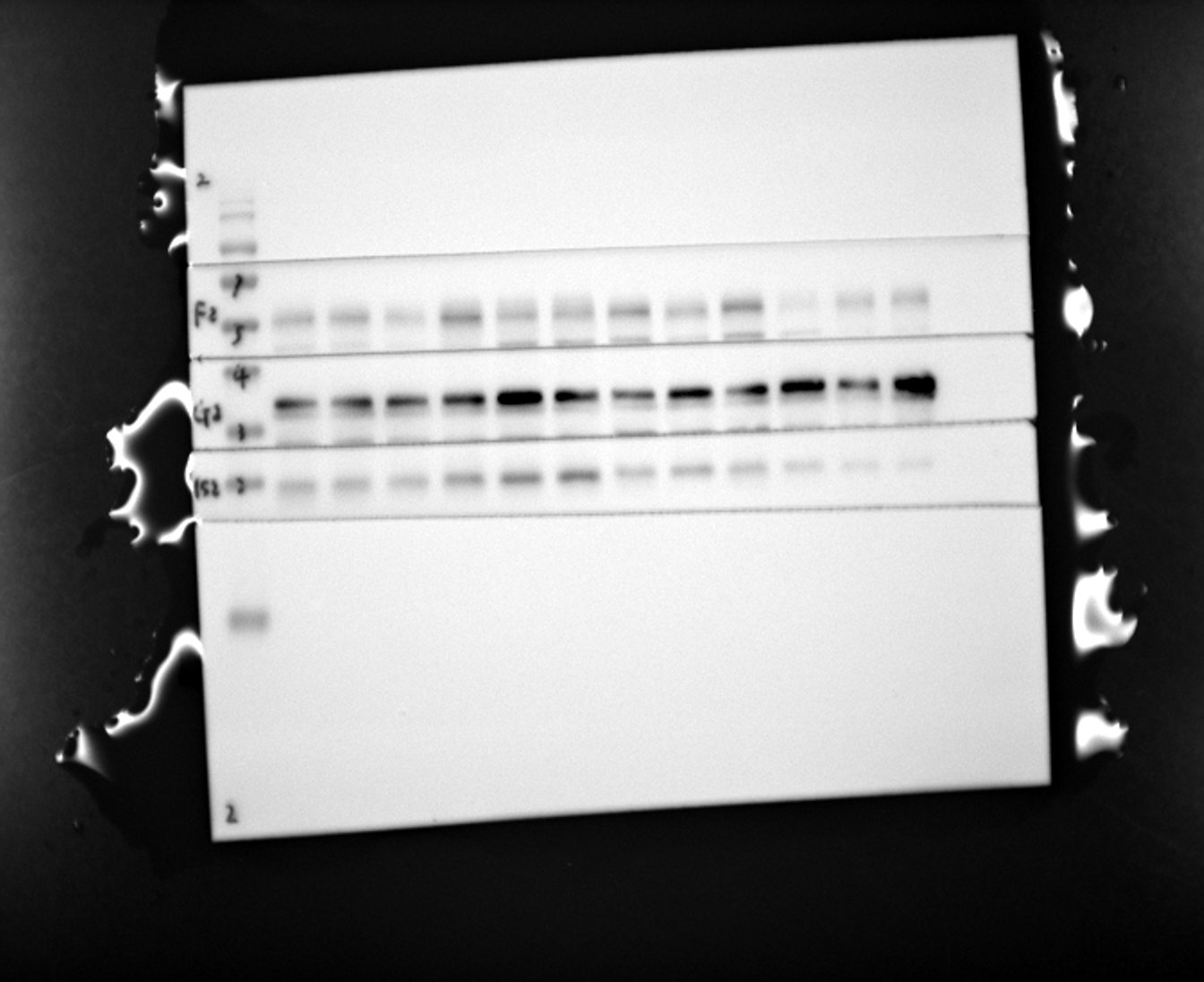

Supplement: Figure S5 [file peerj-11-16407-s008.zip › Figure 5/FXR&FGF15 protein/3/12%-ILEUM-ALL-2-1.Tif]

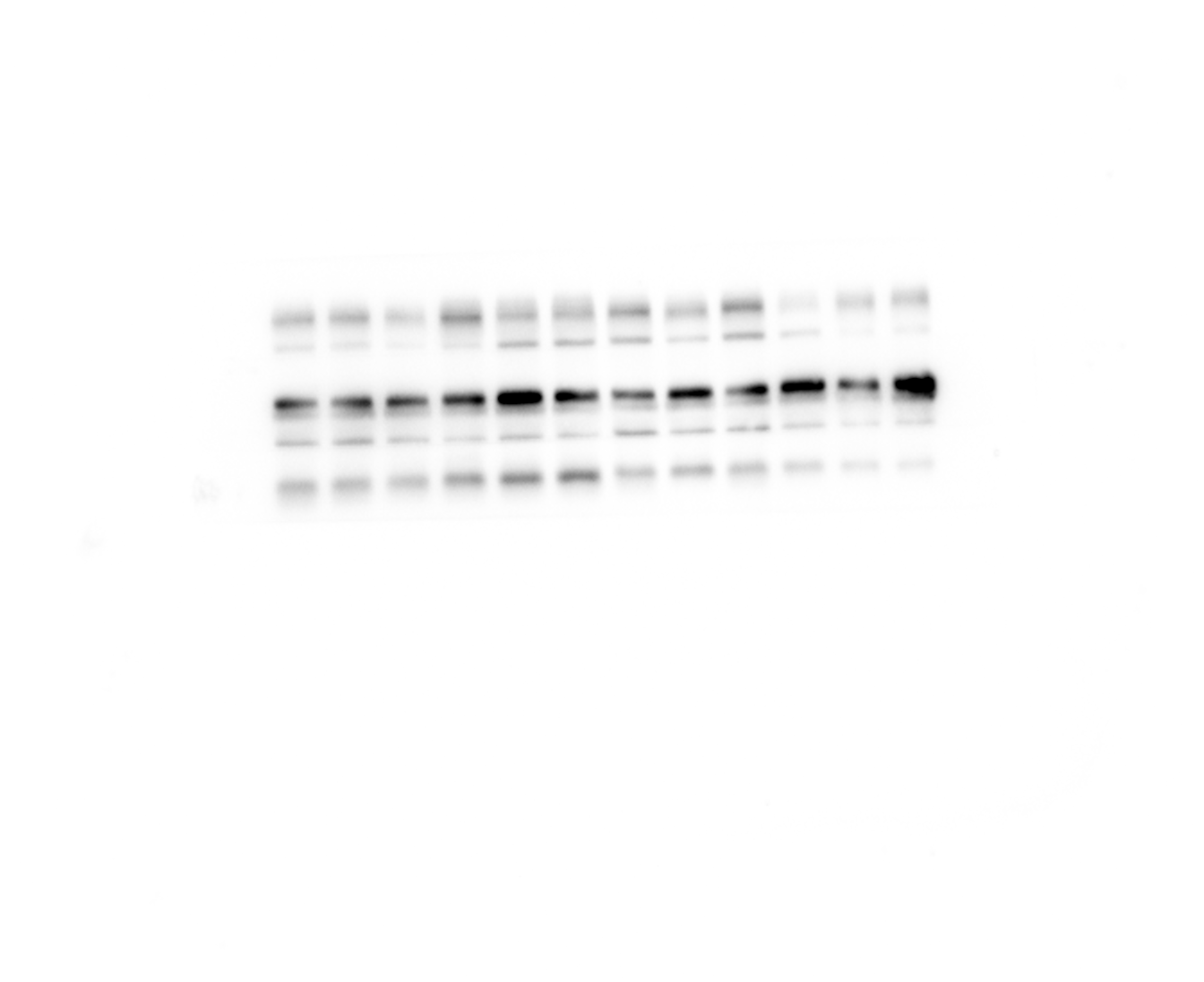

Supplement: Figure S5 [file peerj-11-16407-s008.zip › Figure 5/FXR&FGF15 protein/3/12%-ILEUM-ALL-2.Tif]

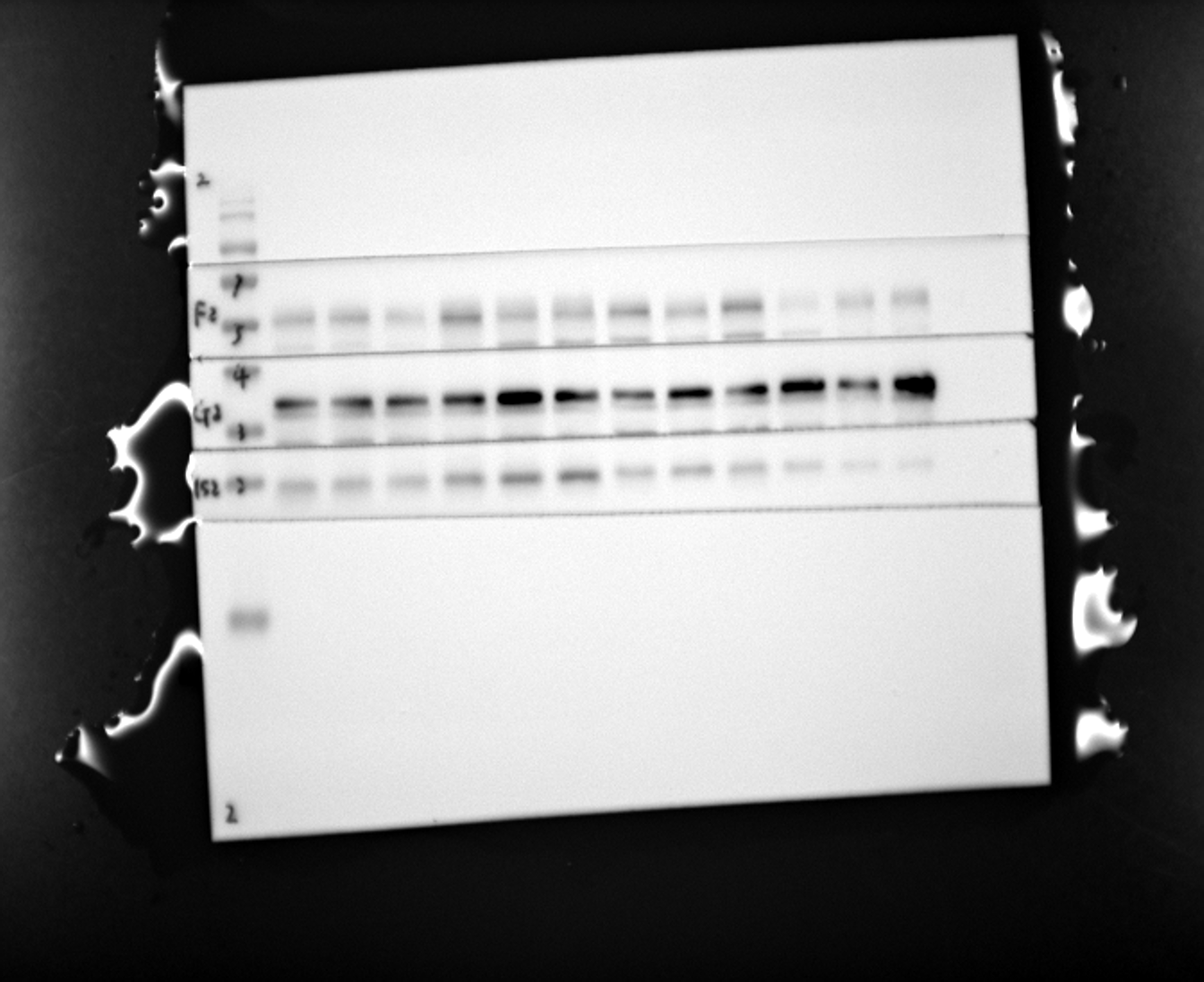

Supplement: Figure S5 [file peerj-11-16407-s008.zip › Figure 5/FXR&FGF15 protein/3/12%-ILEUM-ALL-22-1-10S.Tif]

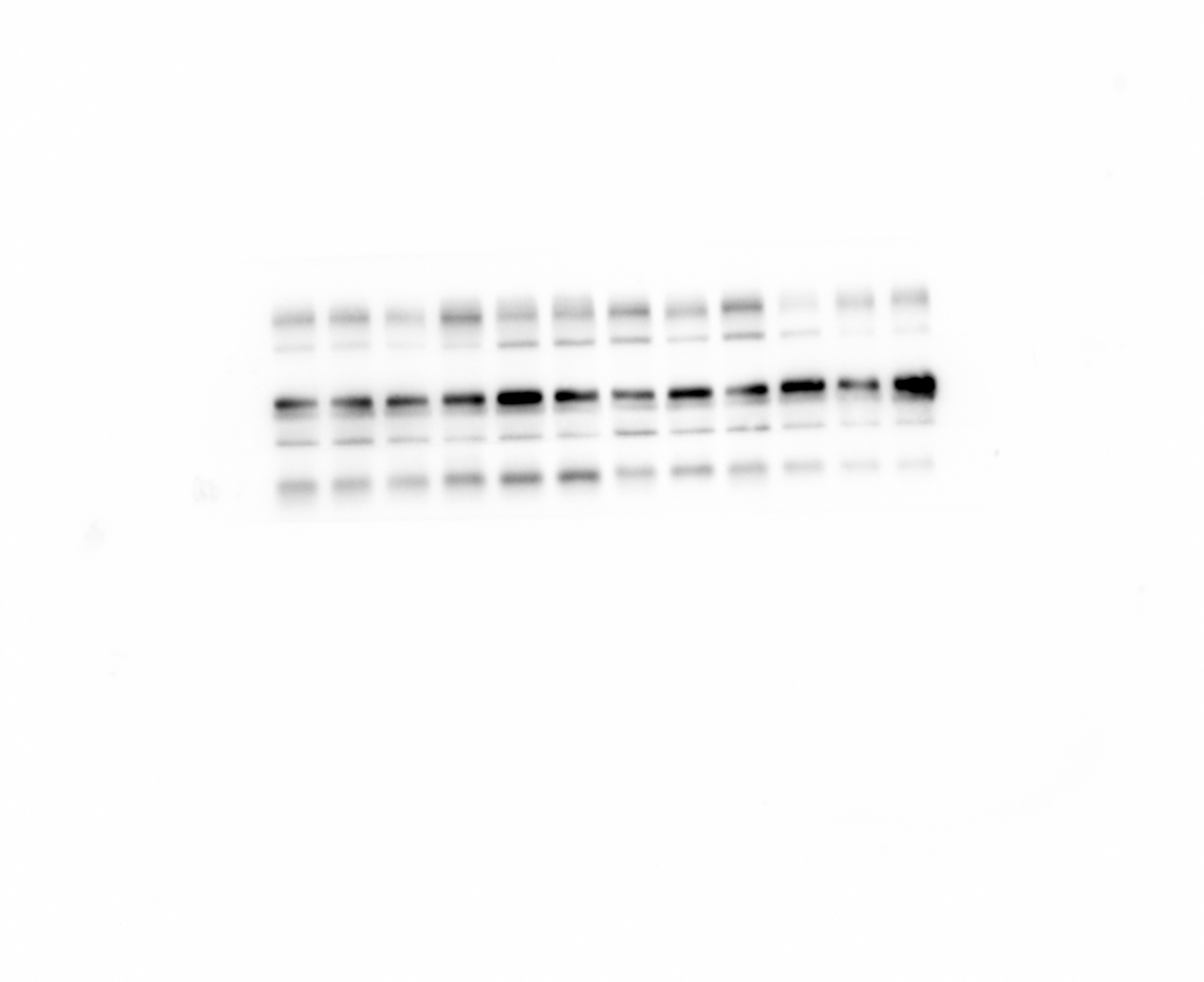

Supplement: Figure S5 [file peerj-11-16407-s008.zip › Figure 5/FXR&FGF15 protein/3/12%-ILEUM-ALL-22.Tif]

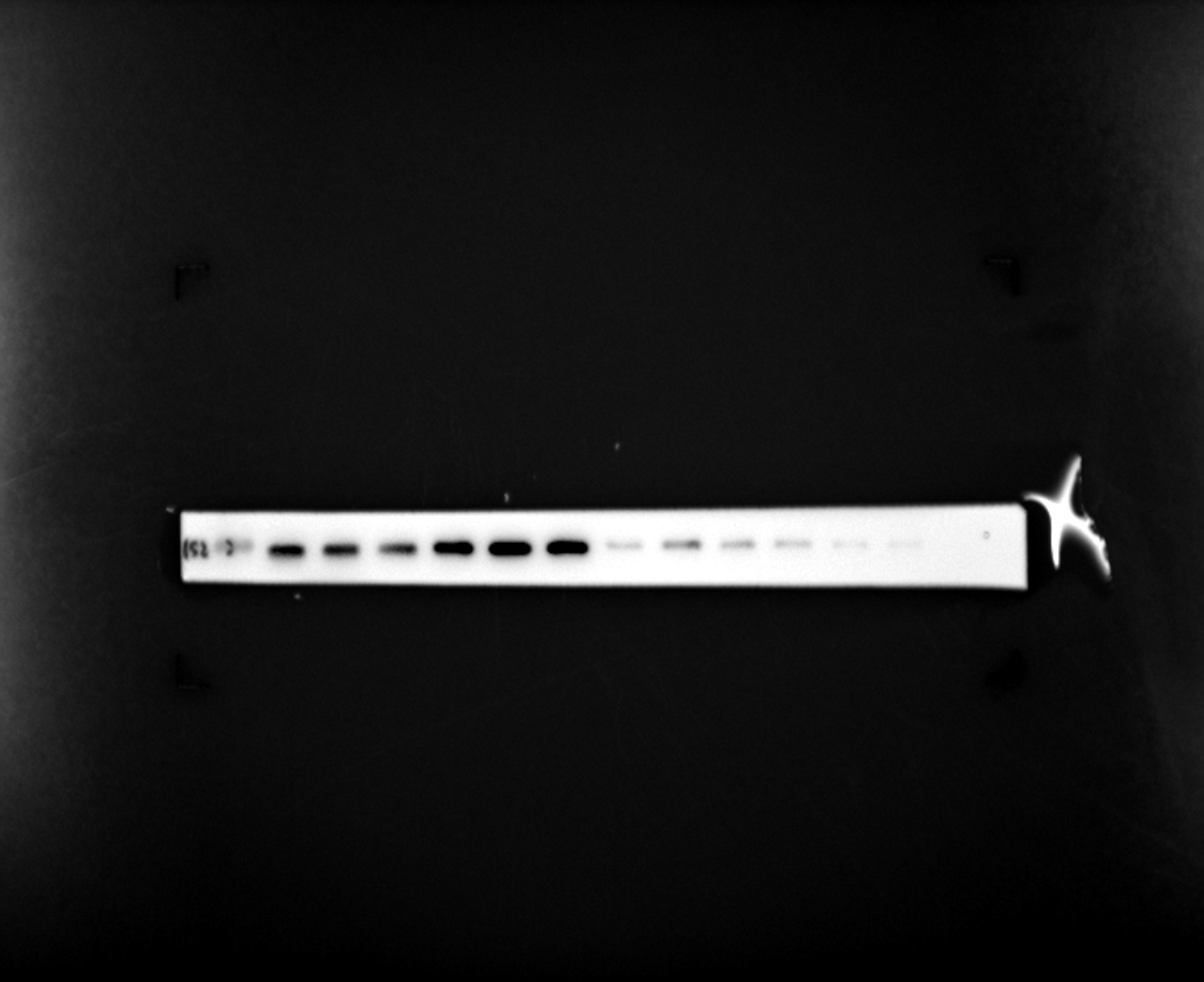

Supplement: Figure S5 [file peerj-11-16407-s008.zip › Figure 5/FXR&FGF15 protein/3/12%-ILEUM-FGF15-2-1.Tif]

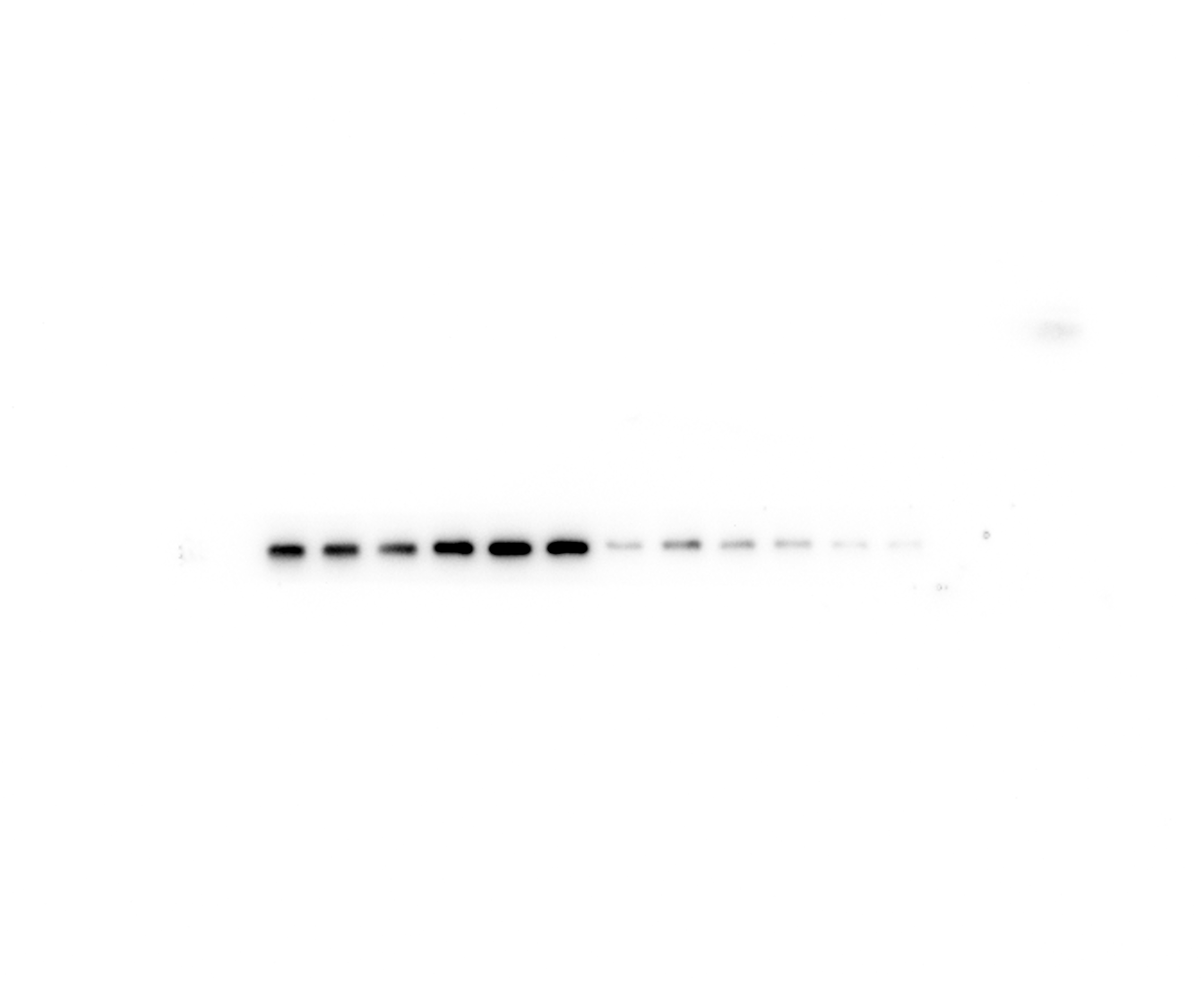

Supplement: Figure S5 [file peerj-11-16407-s008.zip › Figure 5/FXR&FGF15 protein/3/12%-ILEUM-FGF15-2.Tif]

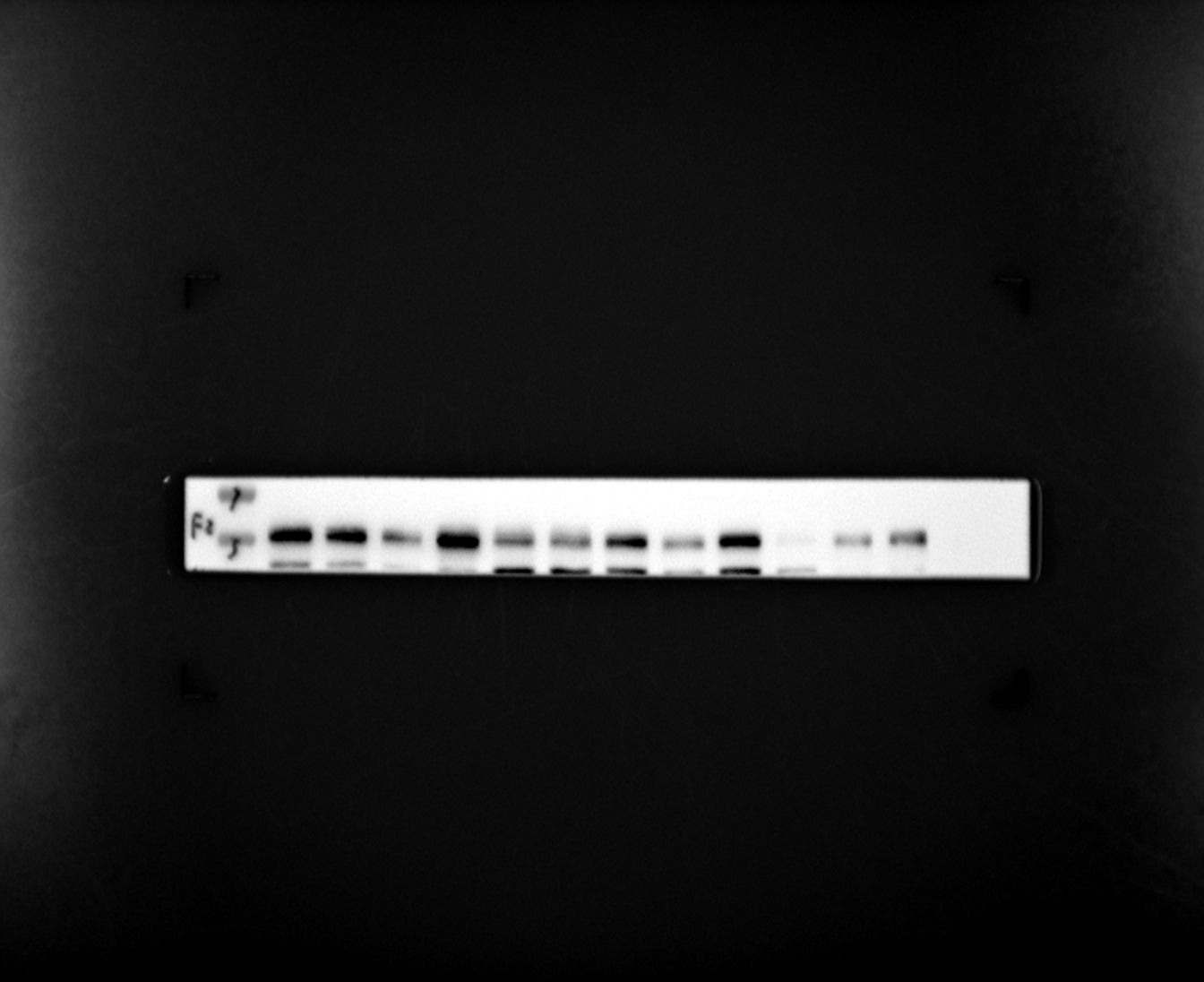

Supplement: Figure S5 [file peerj-11-16407-s008.zip › Figure 5/FXR&FGF15 protein/3/12%-ILEUM-FXR-2-1.Tif]

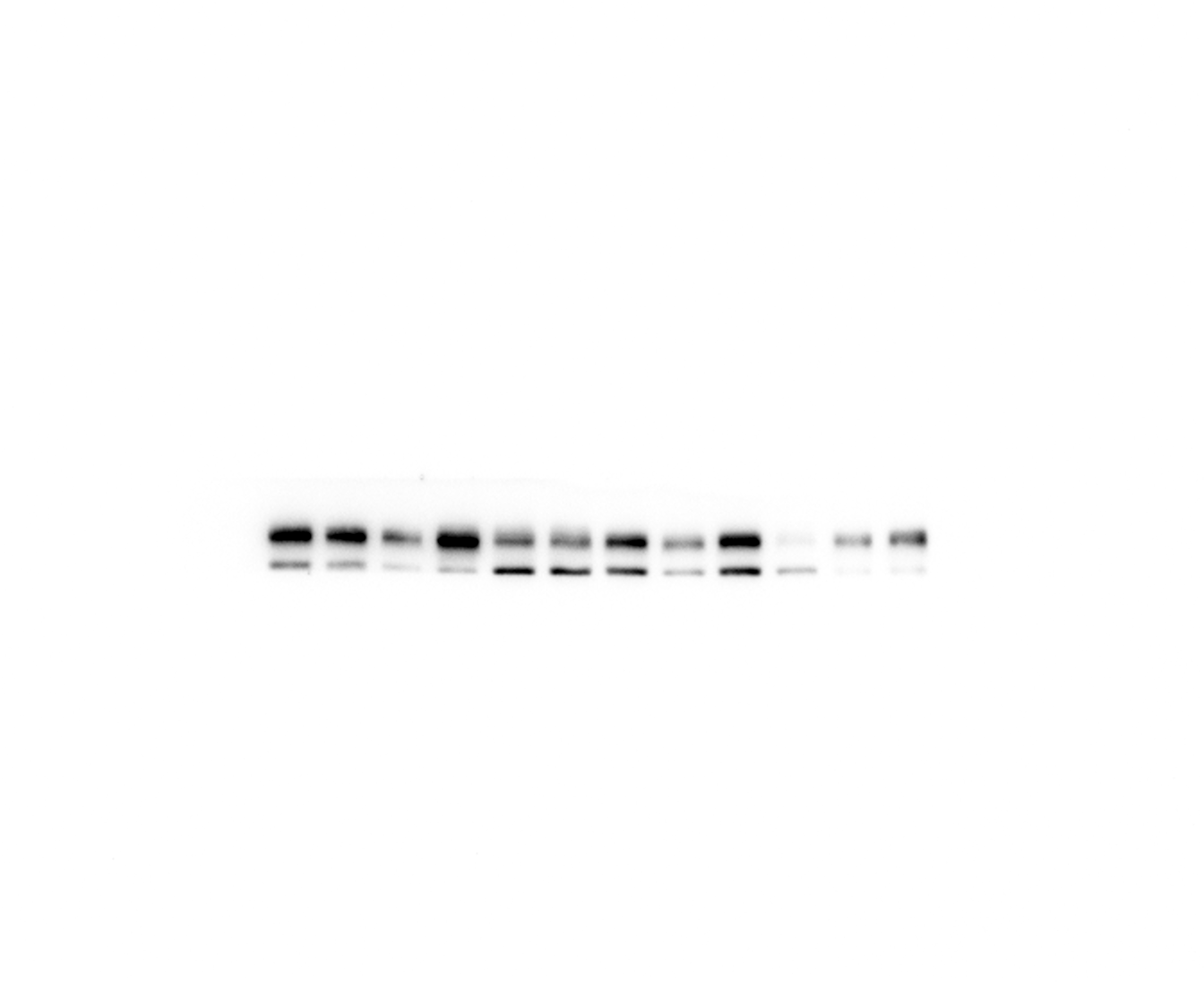

Supplement: Figure S5 [file peerj-11-16407-s008.zip › Figure 5/FXR&FGF15 protein/3/12%-ILEUM-FXR-2.Tif]

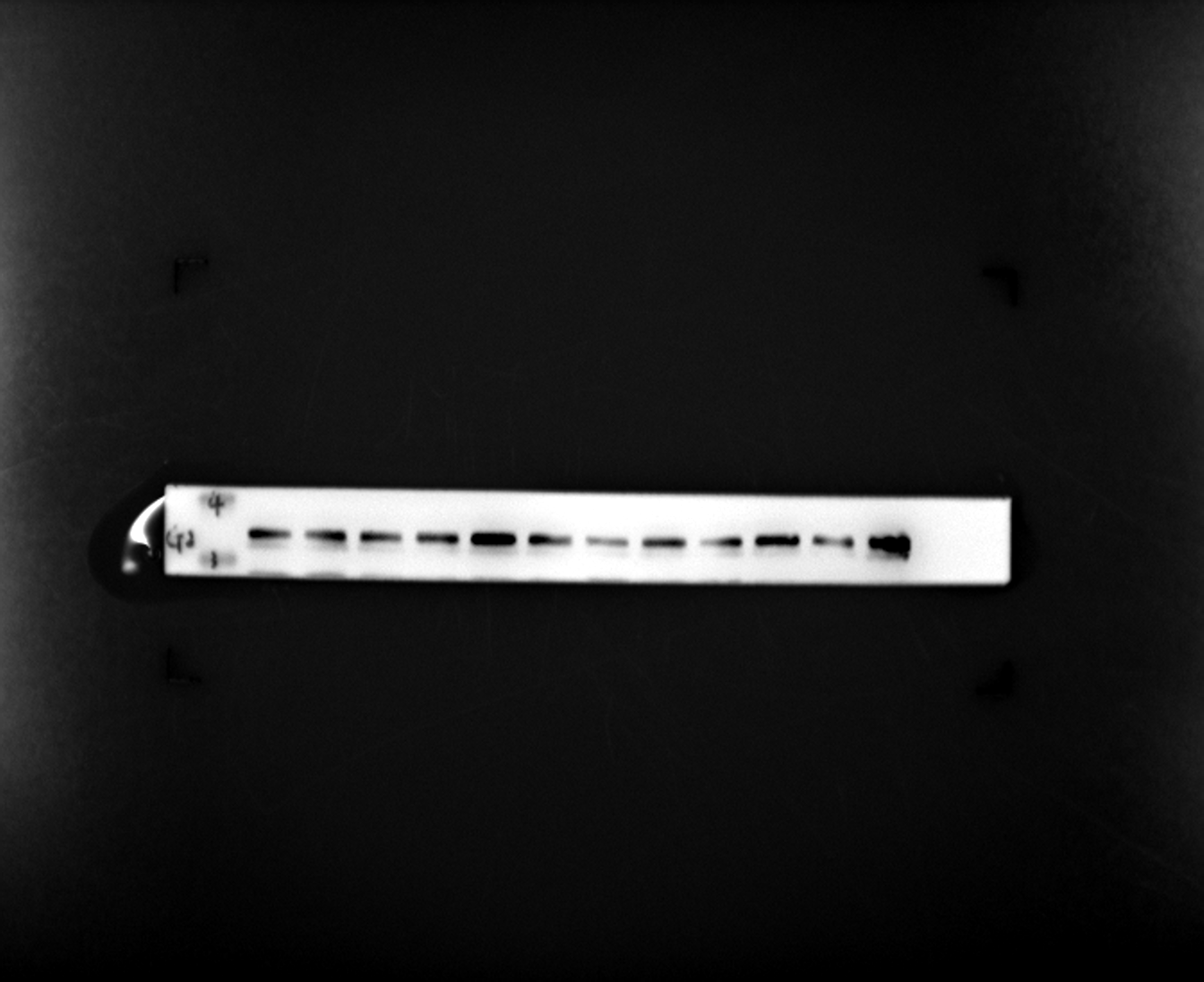

Supplement: Figure S5 [file peerj-11-16407-s008.zip › Figure 5/FXR&FGF15 protein/3/12%-ILEUM-GAPDH-2-1.Tif]

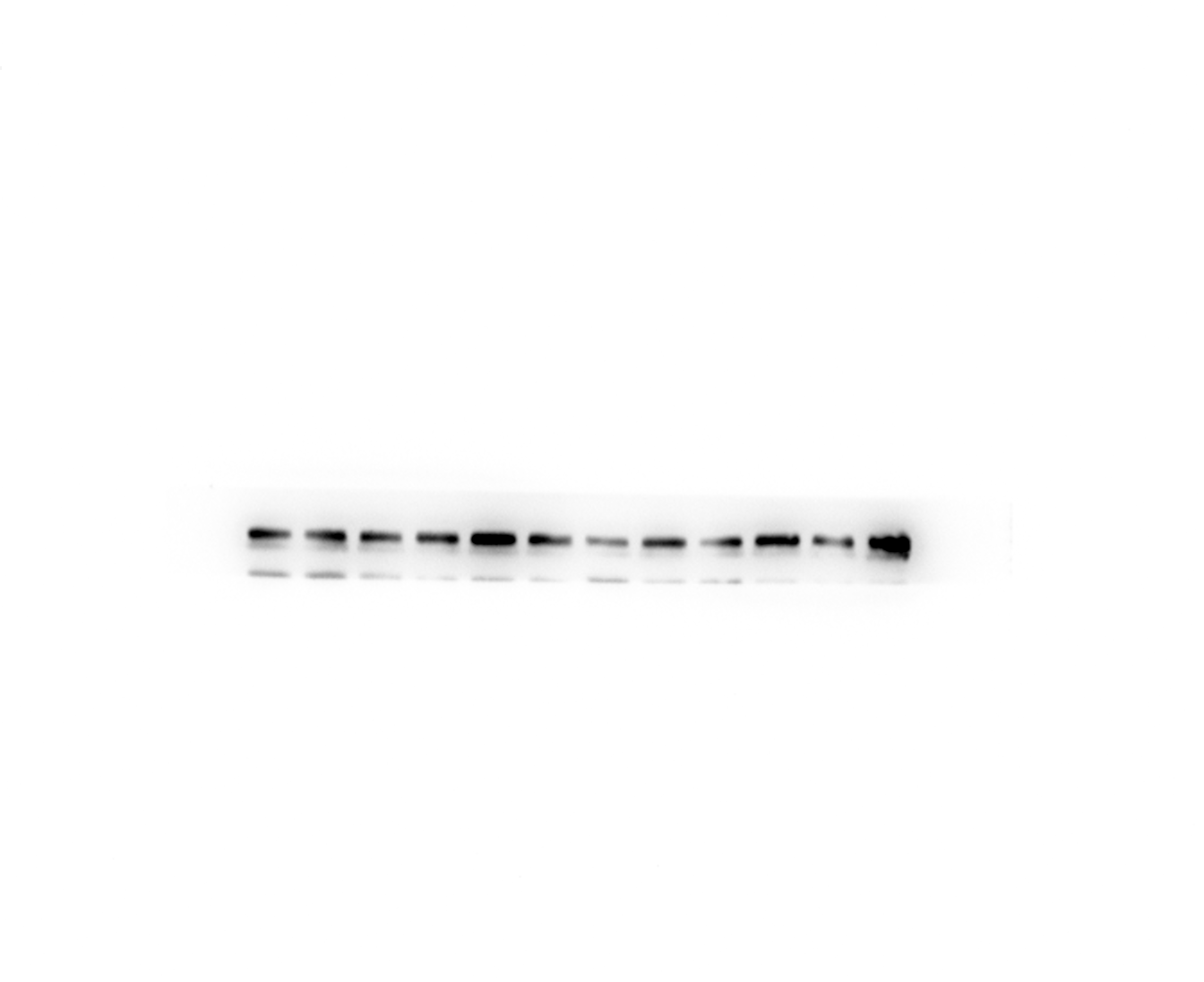

Supplement: Figure S5 [file peerj-11-16407-s008.zip › Figure 5/FXR&FGF15 protein/3/12%-ILEUM-GAPDH-2.Tif]

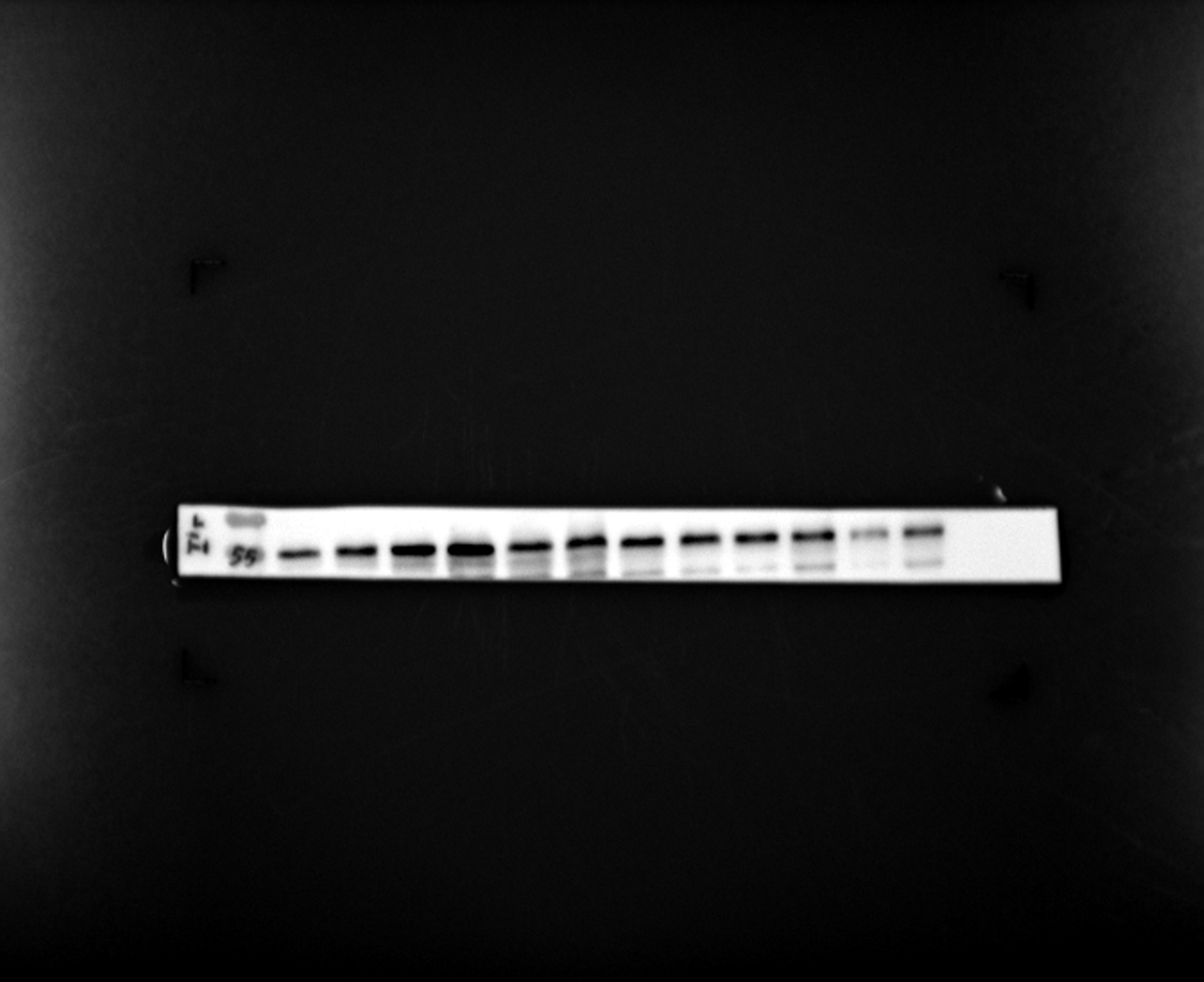

Supplement: Figure S5 [file peerj-11-16407-s008.zip › Figure 5/FXR&SHP protein/Liver-FXR-GAPDH/1/LIVER-12.5%-FXR-1-1.Tif]

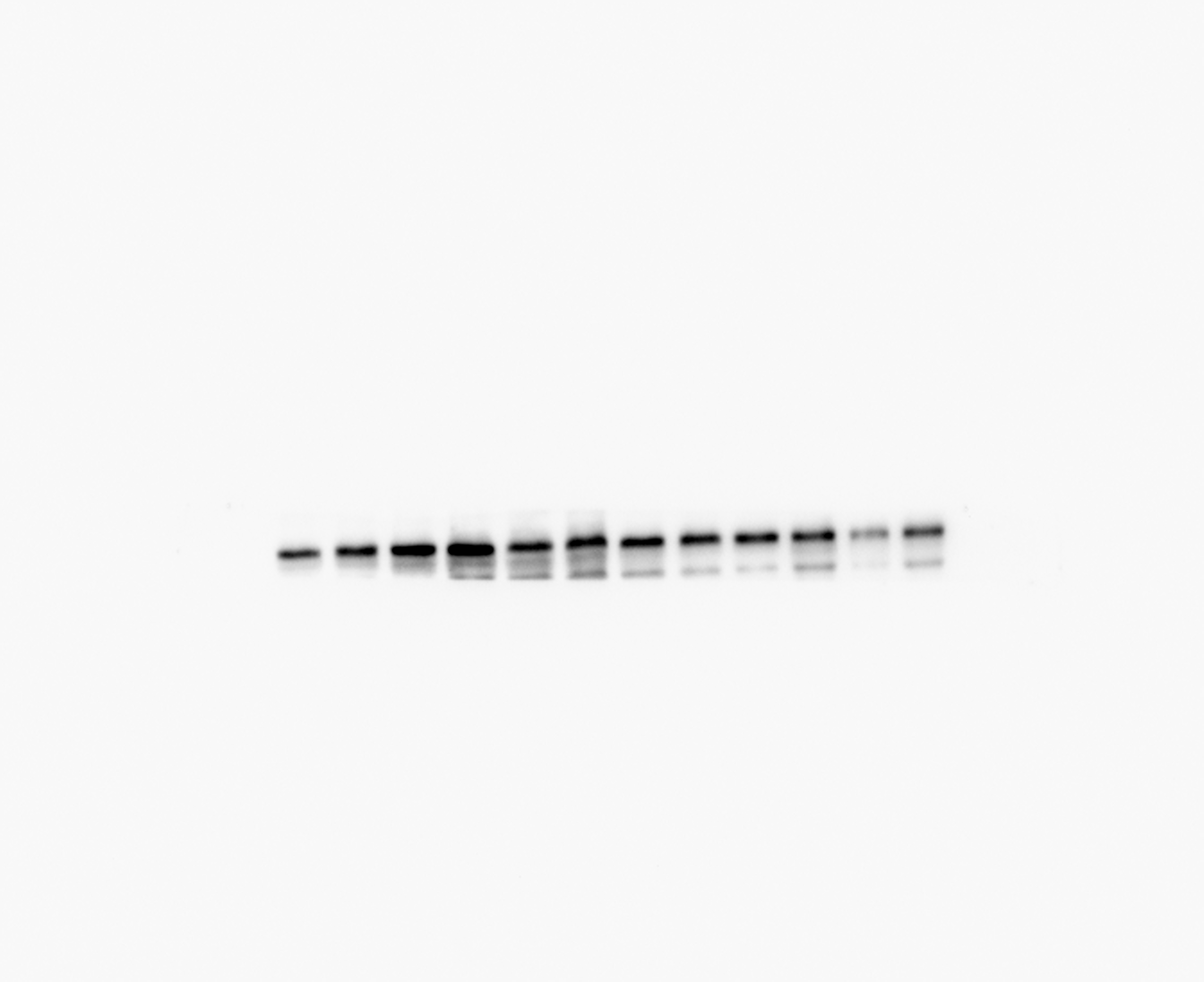

Supplement: Figure S5 [file peerj-11-16407-s008.zip › Figure 5/FXR&SHP protein/Liver-FXR-GAPDH/1/LIVER-12.5%-FXR-1.Tif]

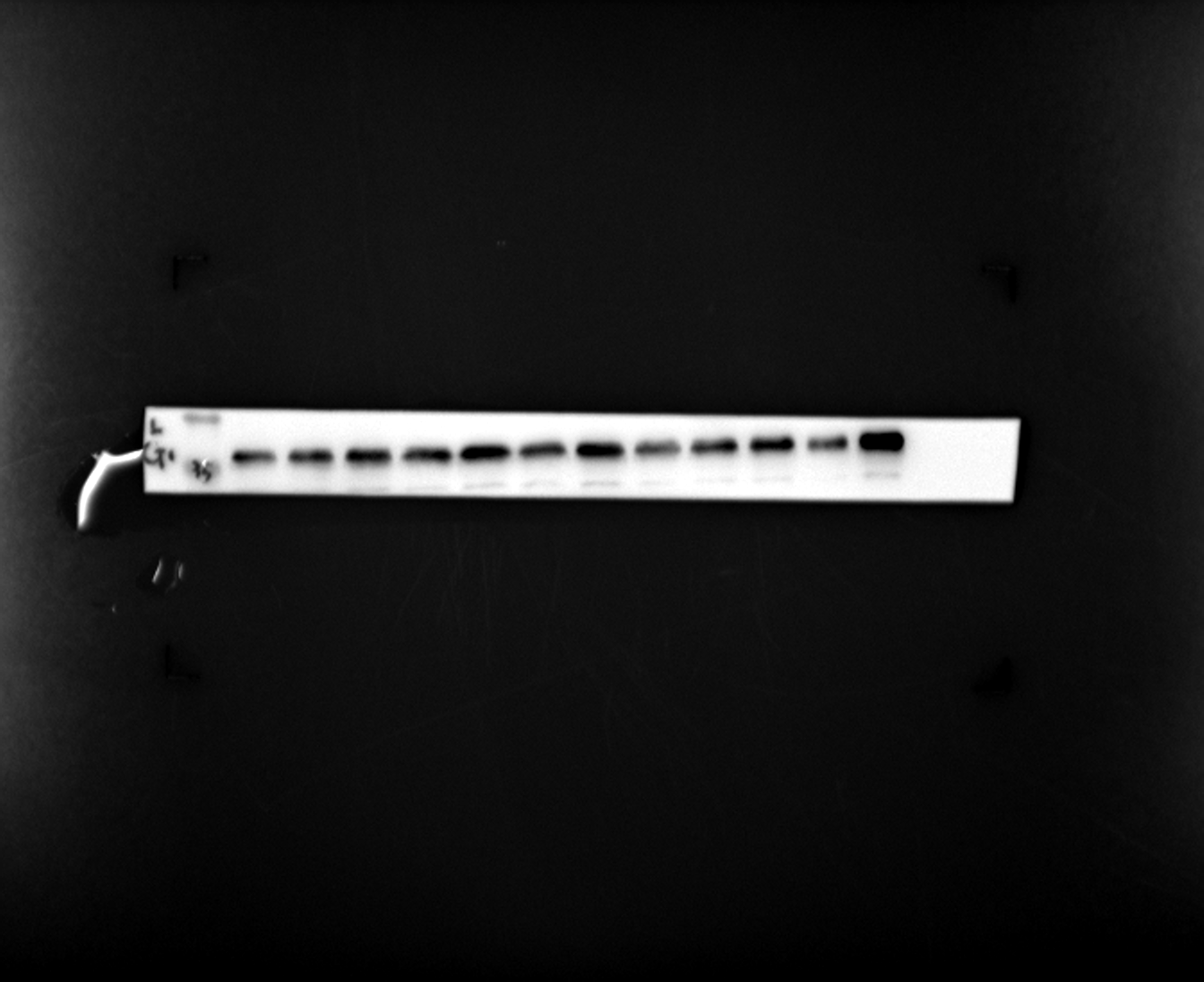

Supplement: Figure S5 [file peerj-11-16407-s008.zip › Figure 5/FXR&SHP protein/Liver-FXR-GAPDH/1/LIVER-12.5%-GAPDH-1-1.Tif]

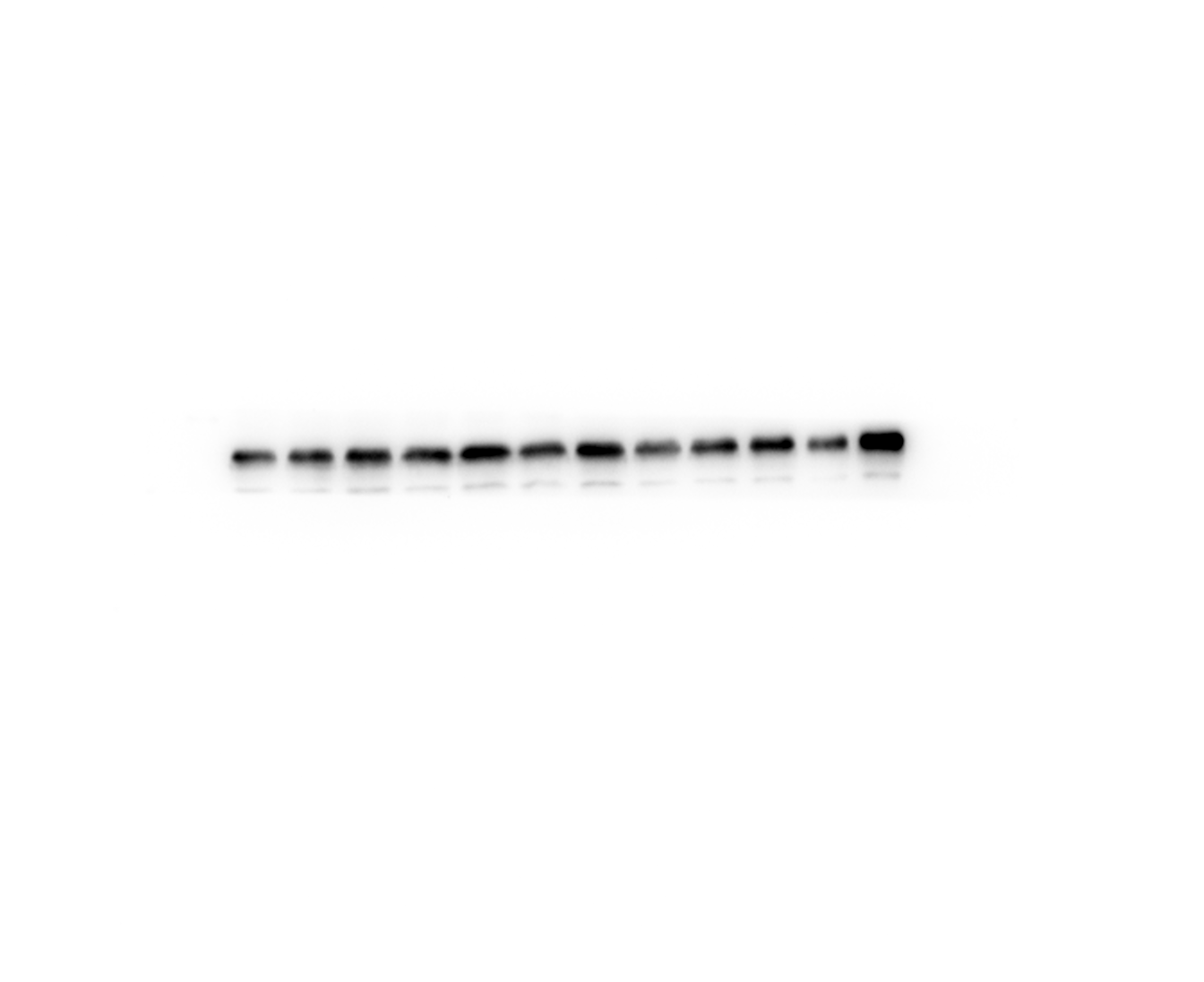

Supplement: Figure S5 [file peerj-11-16407-s008.zip › Figure 5/FXR&SHP protein/Liver-FXR-GAPDH/1/LIVER-12.5%-GAPDH-1.Tif]

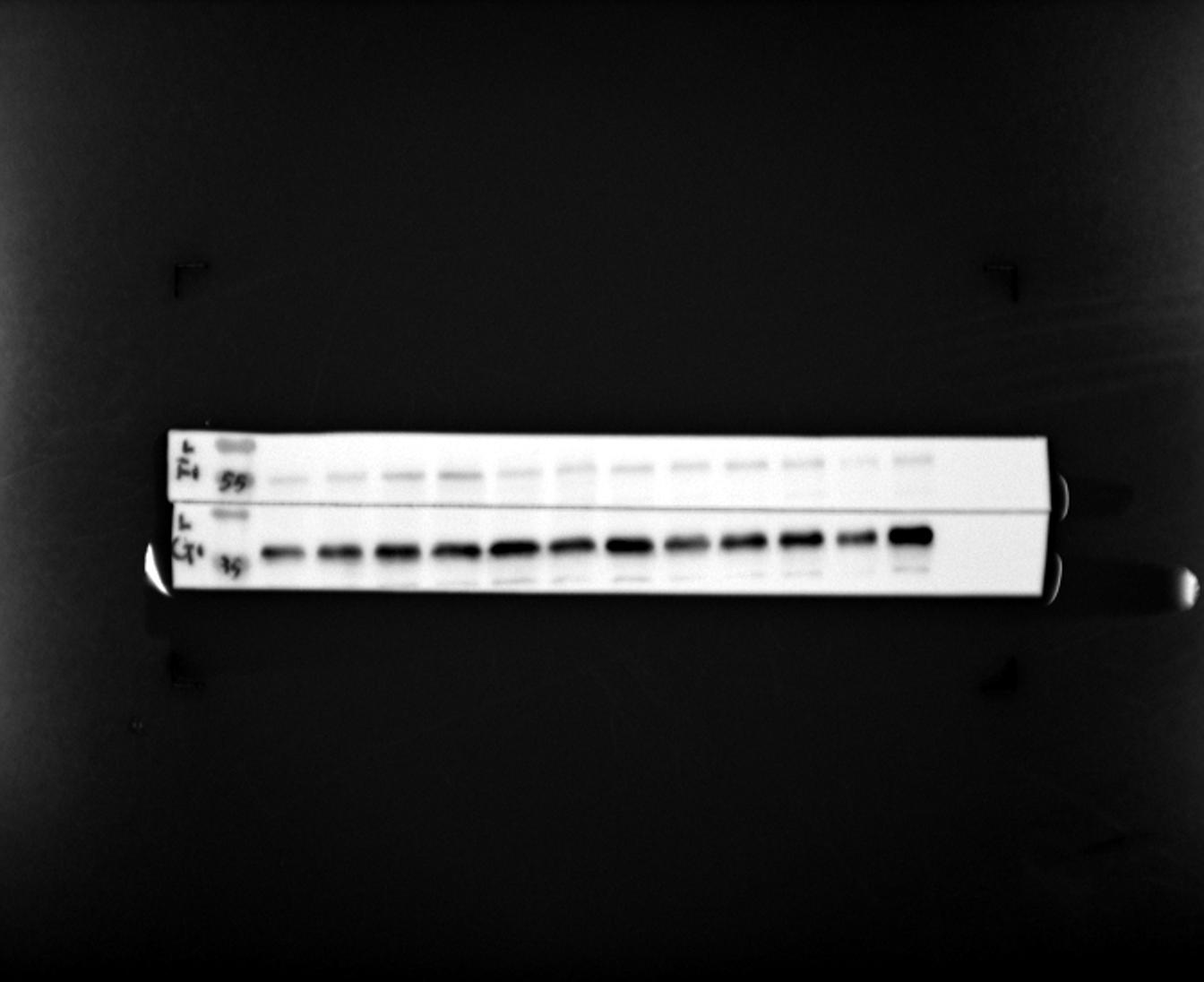

Supplement: Figure S5 [file peerj-11-16407-s008.zip › Figure 5/FXR&SHP protein/Liver-FXR-GAPDH/1/LIVER-12.5%-GAPDH-FXR-1-1.Tif]

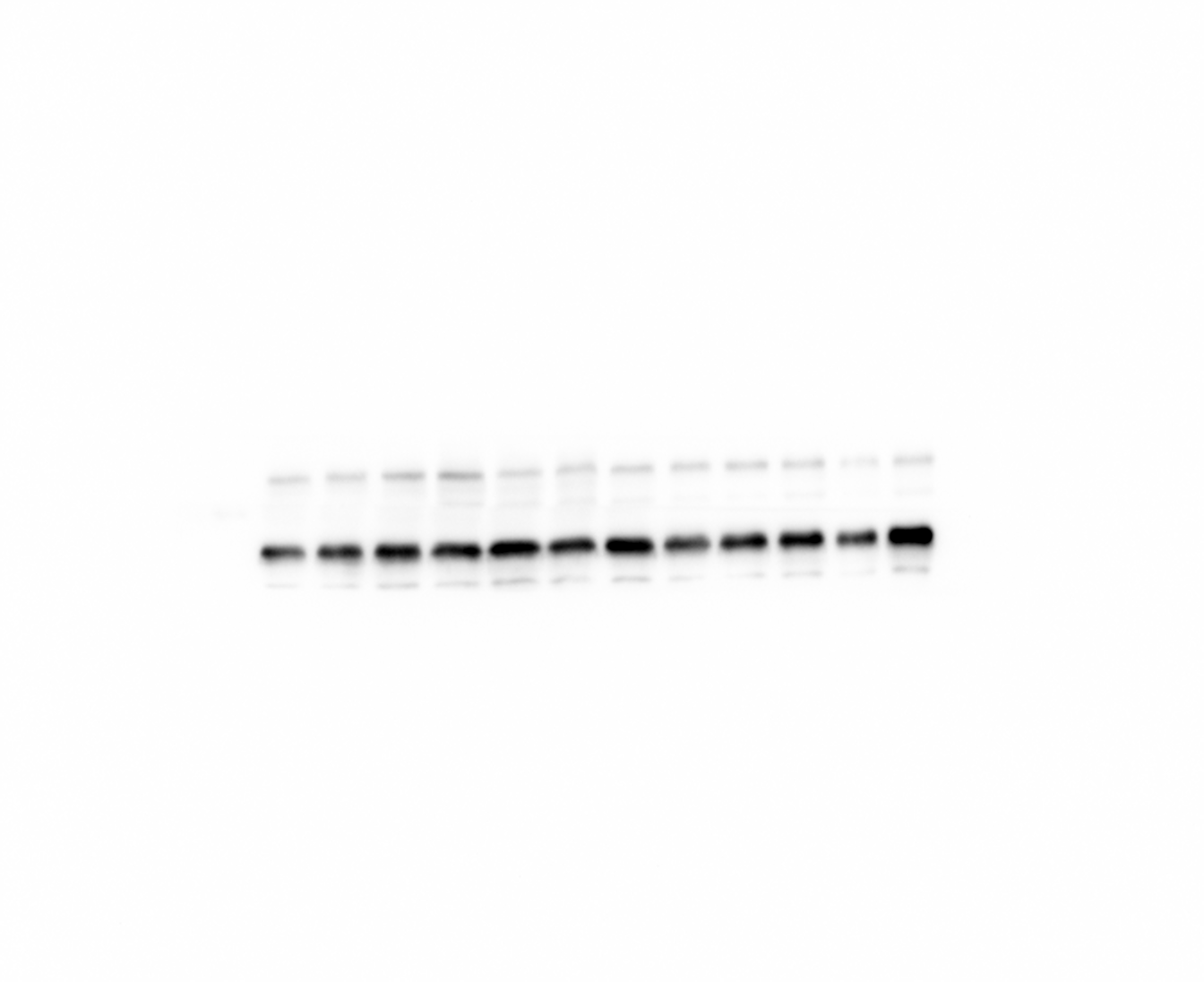

Supplement: Figure S5 [file peerj-11-16407-s008.zip › Figure 5/FXR&SHP protein/Liver-FXR-GAPDH/1/LIVER-12.5%-GAPDH-FXR-1.Tif]

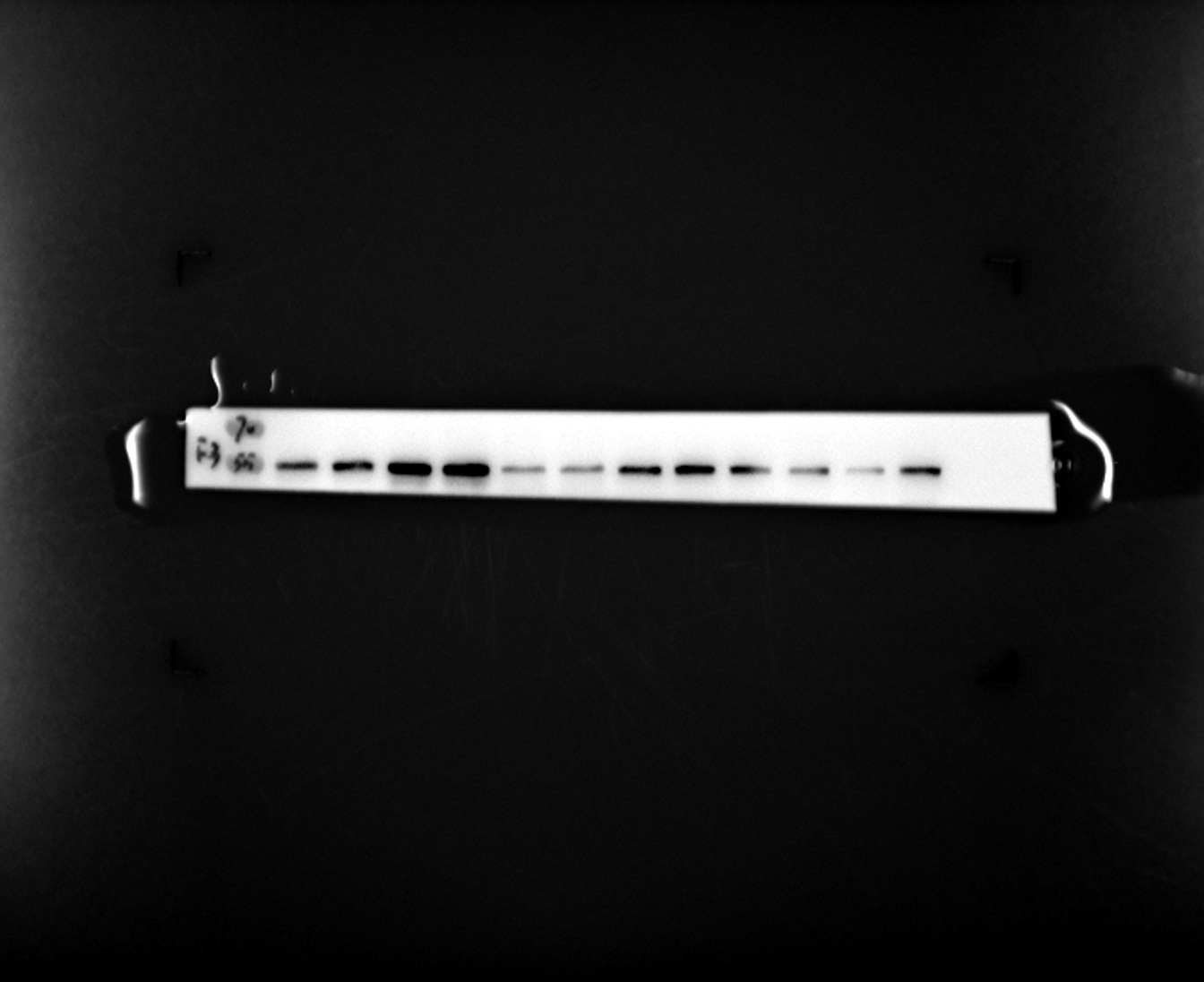

Supplement: Figure S5 [file peerj-11-16407-s008.zip › Figure 5/FXR&SHP protein/Liver-FXR-GAPDH/2/LIVER-12.5%-FXR-2-1.Tif]

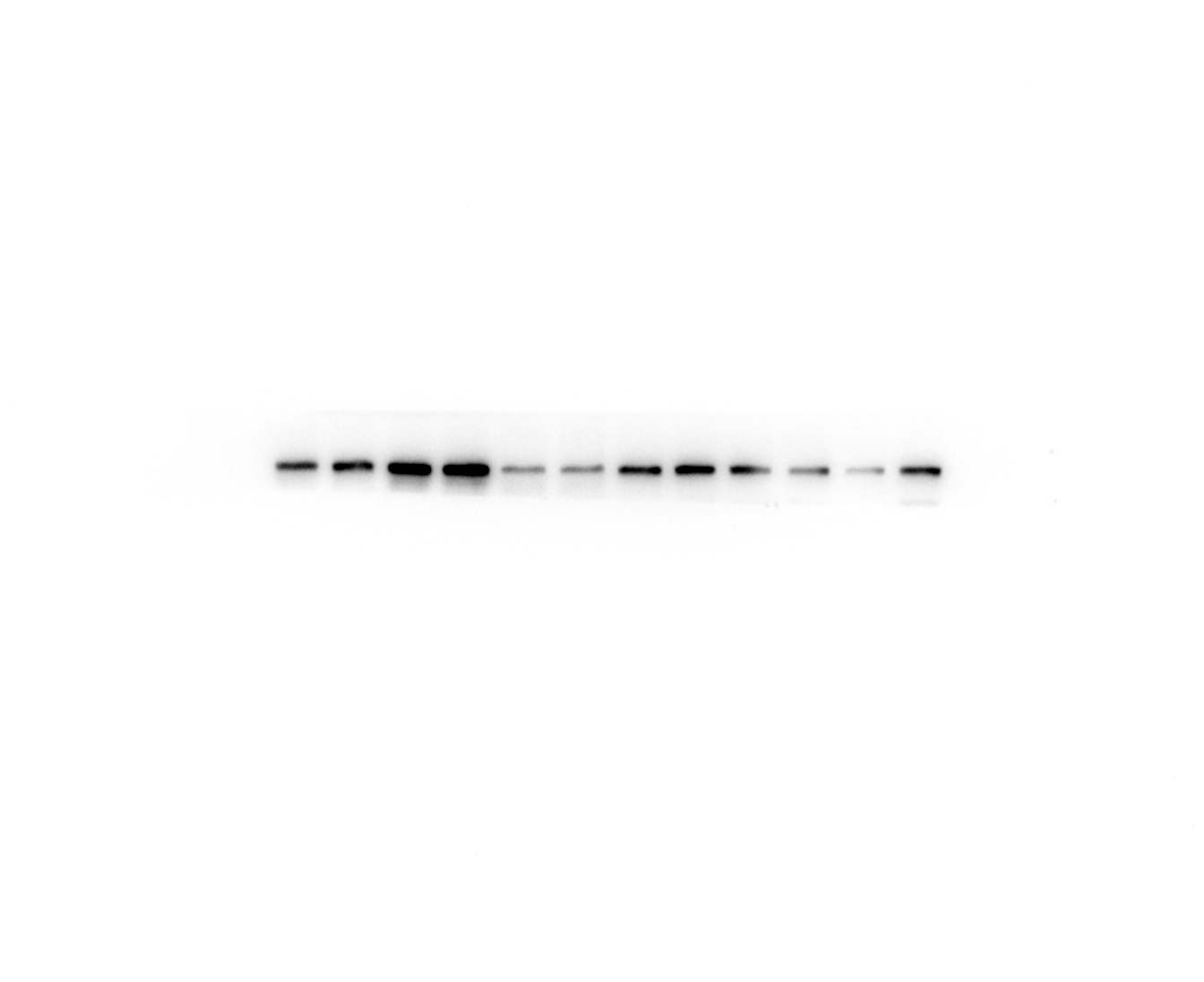

Supplement: Figure S5 [file peerj-11-16407-s008.zip › Figure 5/FXR&SHP protein/Liver-FXR-GAPDH/2/LIVER-12.5%-FXR-2.Tif]

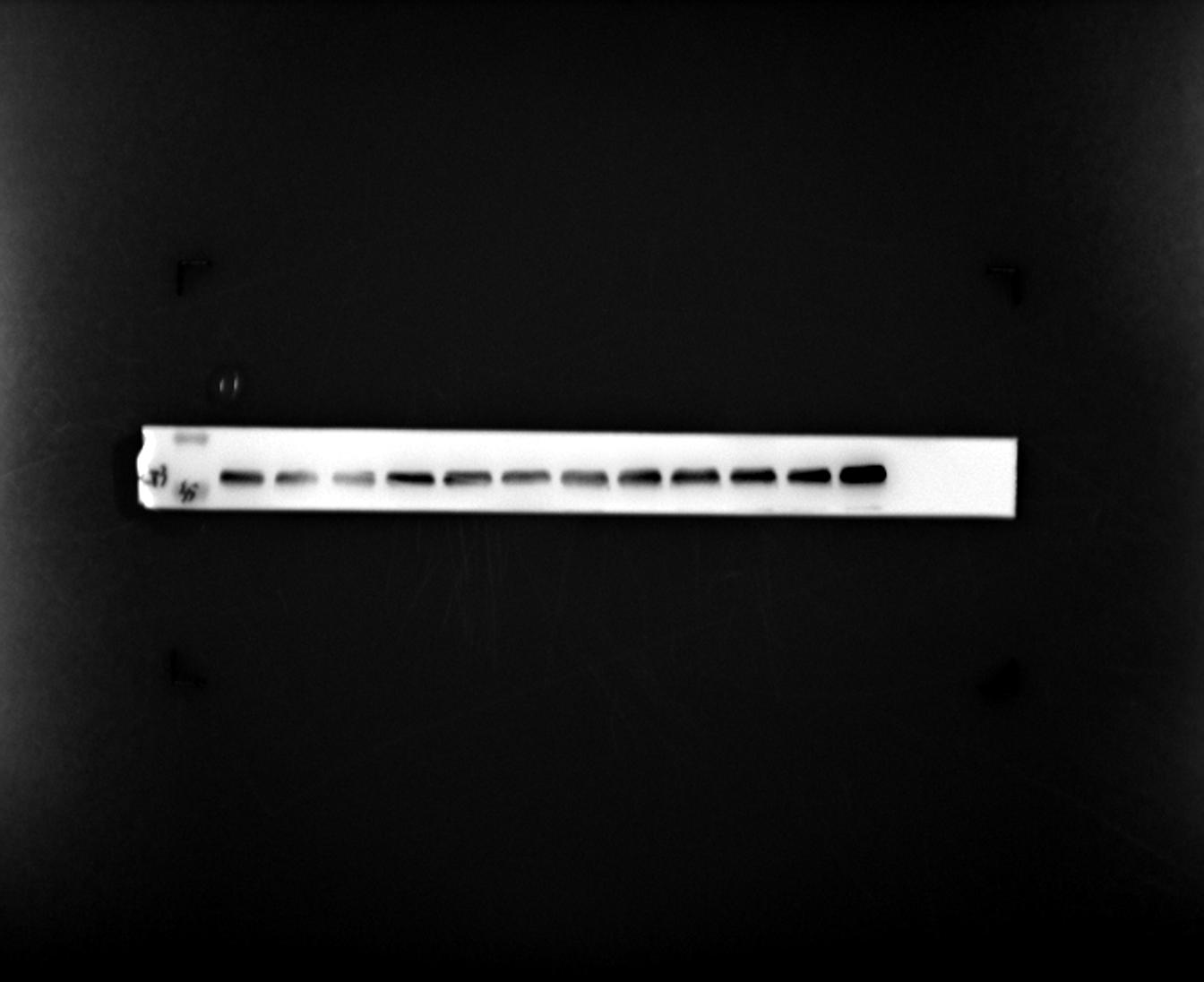

Supplement: Figure S5 [file peerj-11-16407-s008.zip › Figure 5/FXR&SHP protein/Liver-FXR-GAPDH/2/LIVER-12.5%-GAPDH-2-1.Tif]

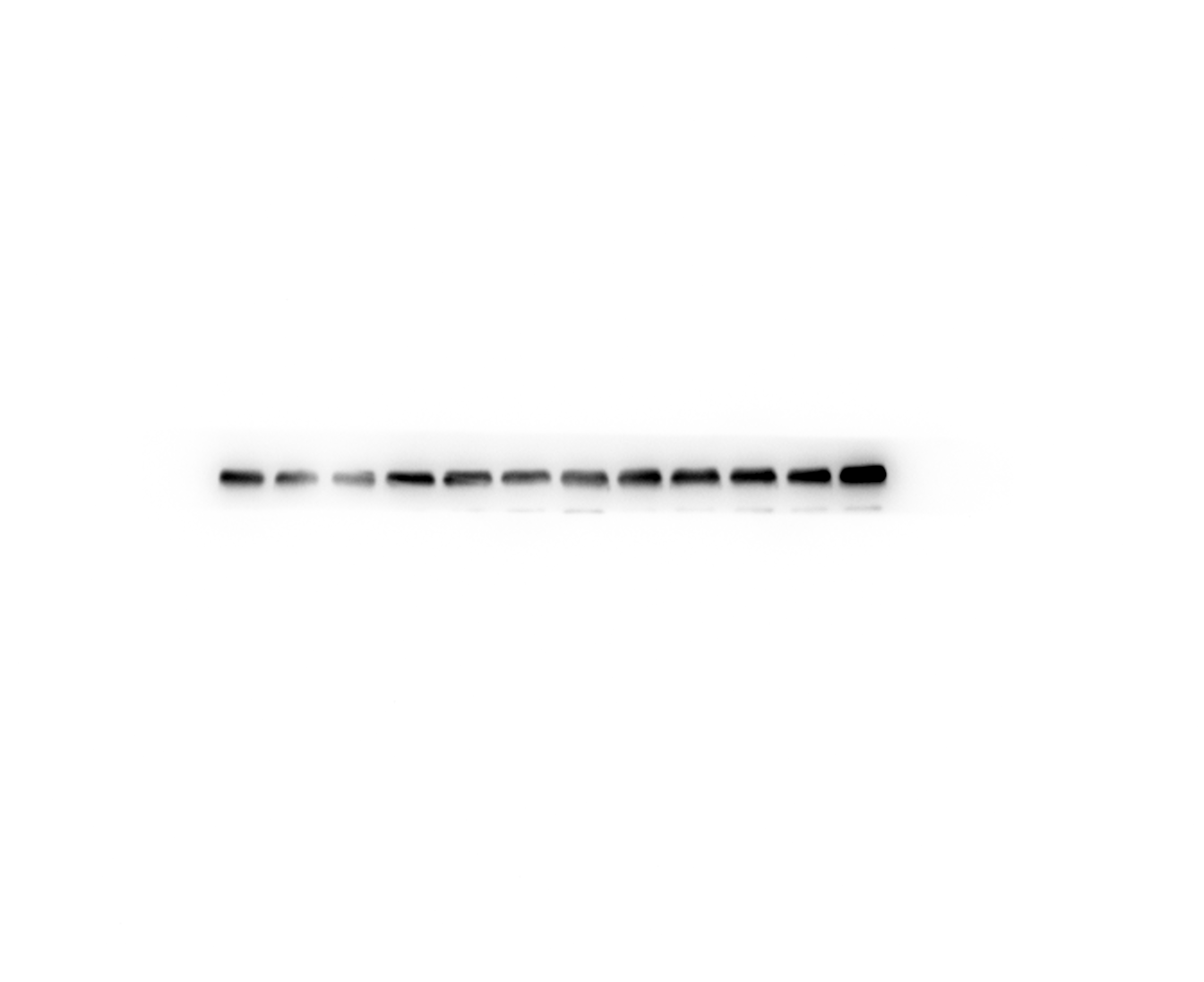

Supplement: Figure S5 [file peerj-11-16407-s008.zip › Figure 5/FXR&SHP protein/Liver-FXR-GAPDH/2/LIVER-12.5%-GAPDH-2.Tif]

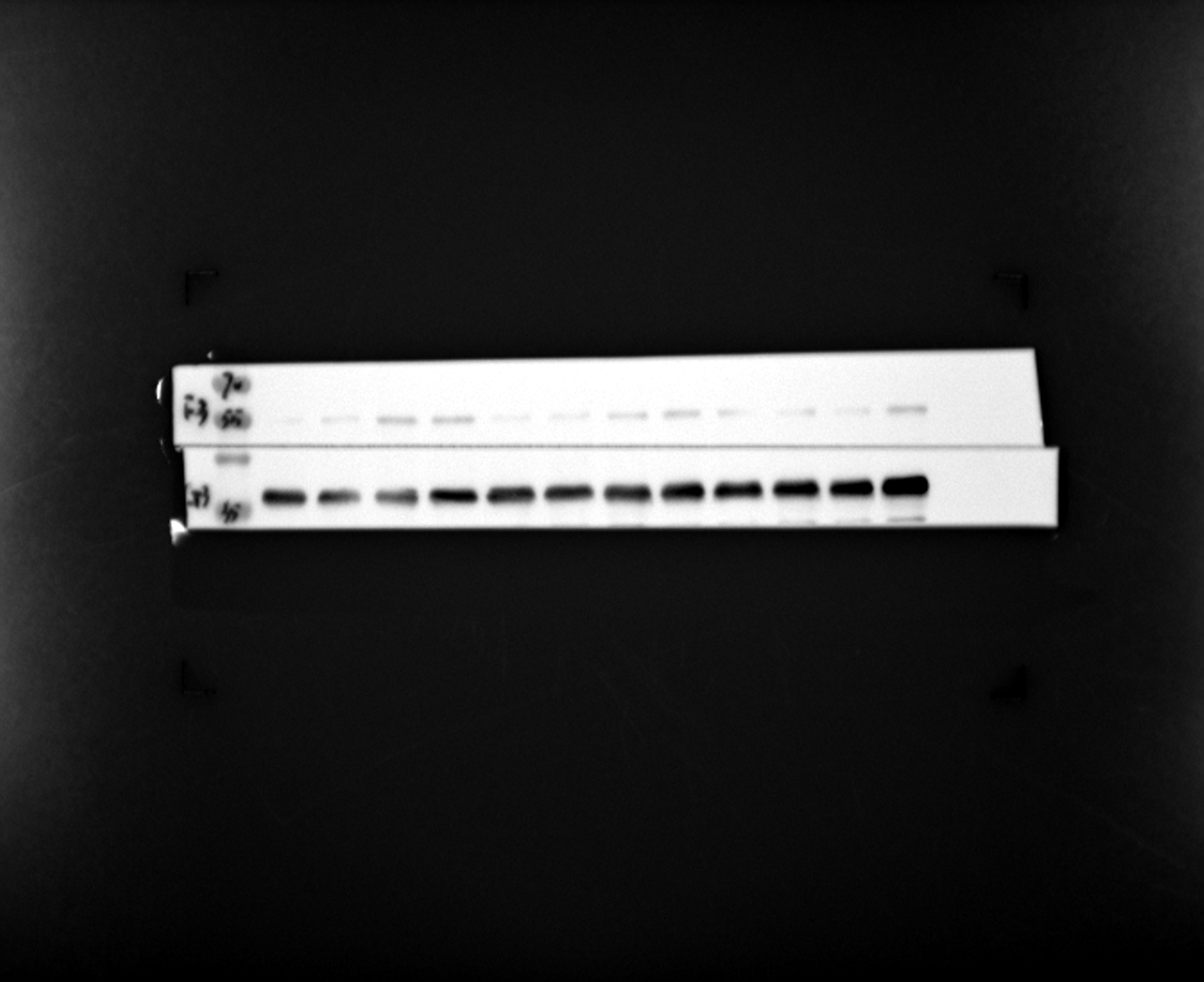

Supplement: Figure S5 [file peerj-11-16407-s008.zip › Figure 5/FXR&SHP protein/Liver-FXR-GAPDH/2/LIVER-12.5%-GAPDH-FXR-2-1-0918-full.Tif]

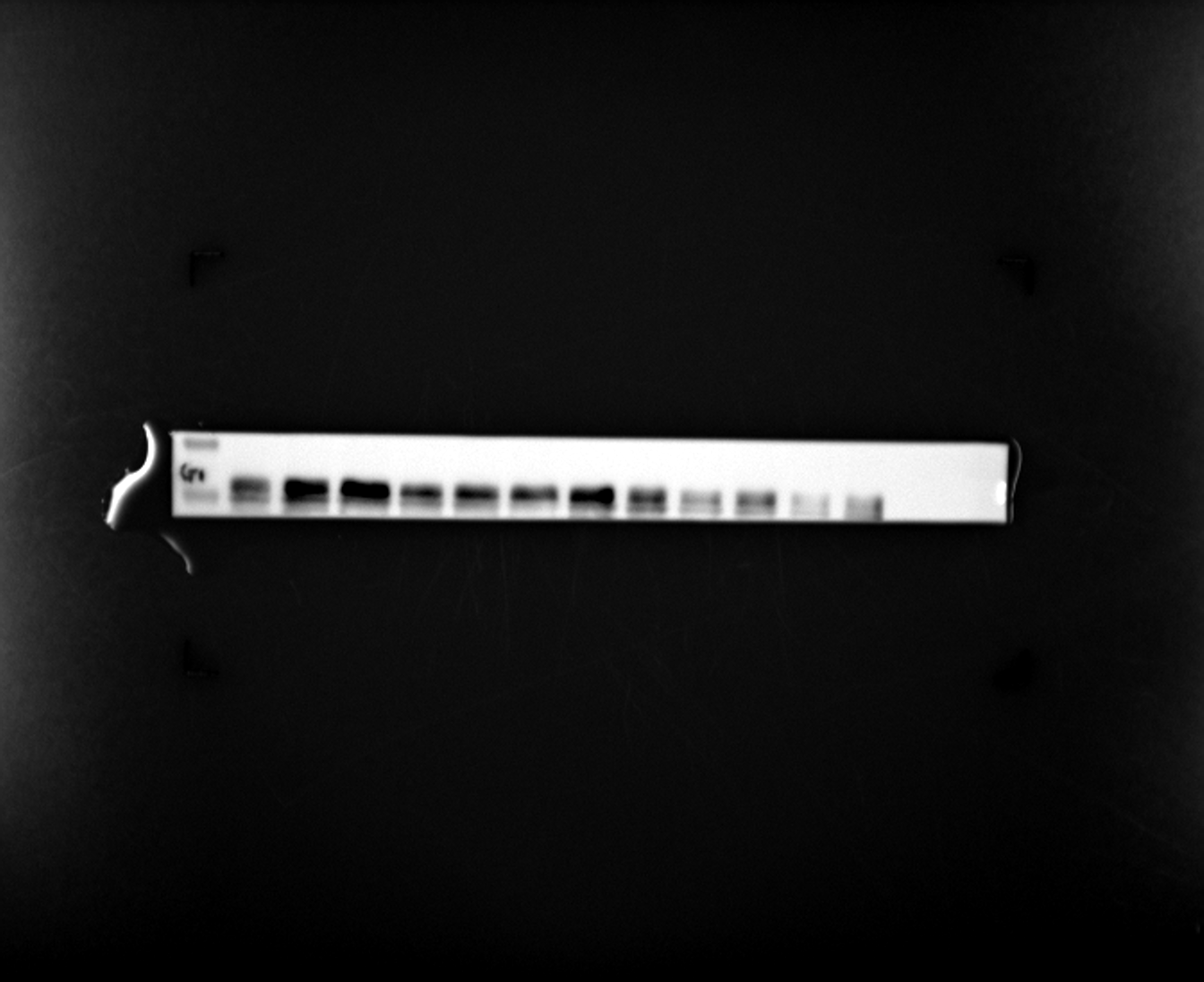

Supplement: Figure S5 [file peerj-11-16407-s008.zip › Figure 5/FXR&SHP protein/Liver-FXR-GAPDH/3/LIVER-12.5%-GAPDH-1-1.Tif]

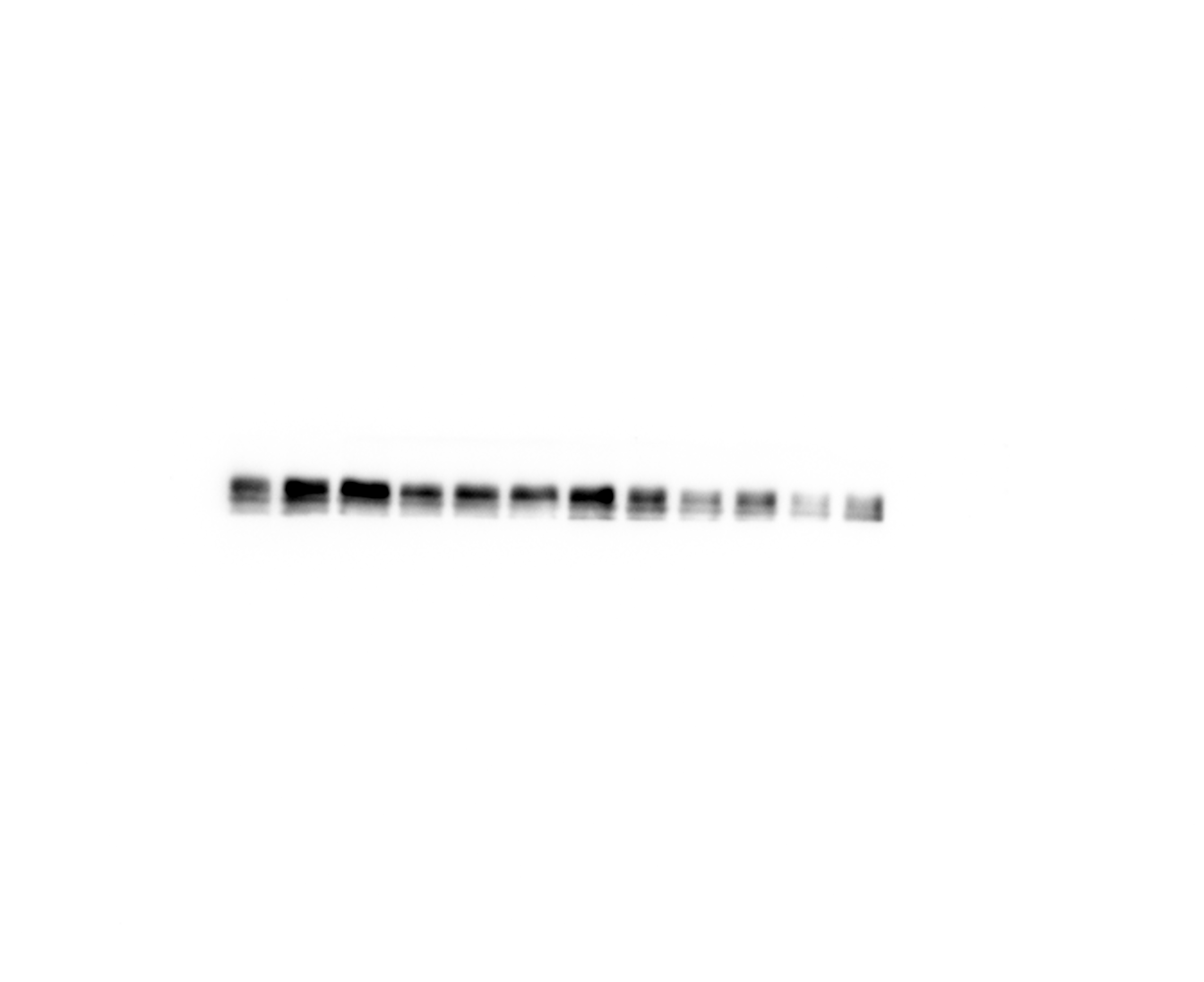

Supplement: Figure S5 [file peerj-11-16407-s008.zip › Figure 5/FXR&SHP protein/Liver-FXR-GAPDH/3/LIVER-12.5%-GAPDH-1.Tif]

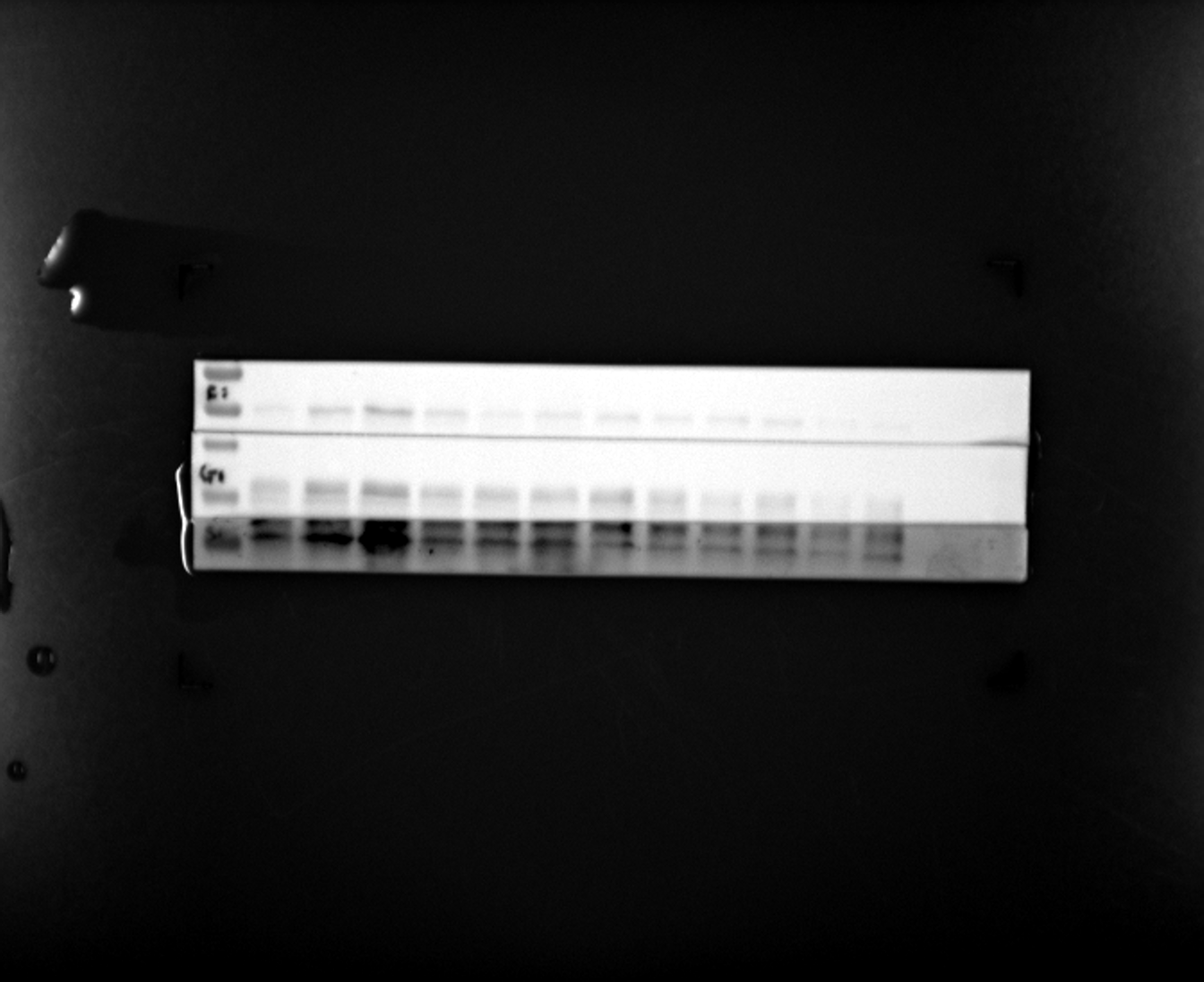

Supplement: Figure S5 [file peerj-11-16407-s008.zip › Figure 5/FXR&SHP protein/Liver-FXR-GAPDH/3/LIVER-12.5%-GAPDH-FXR-1-1-full.Tif]

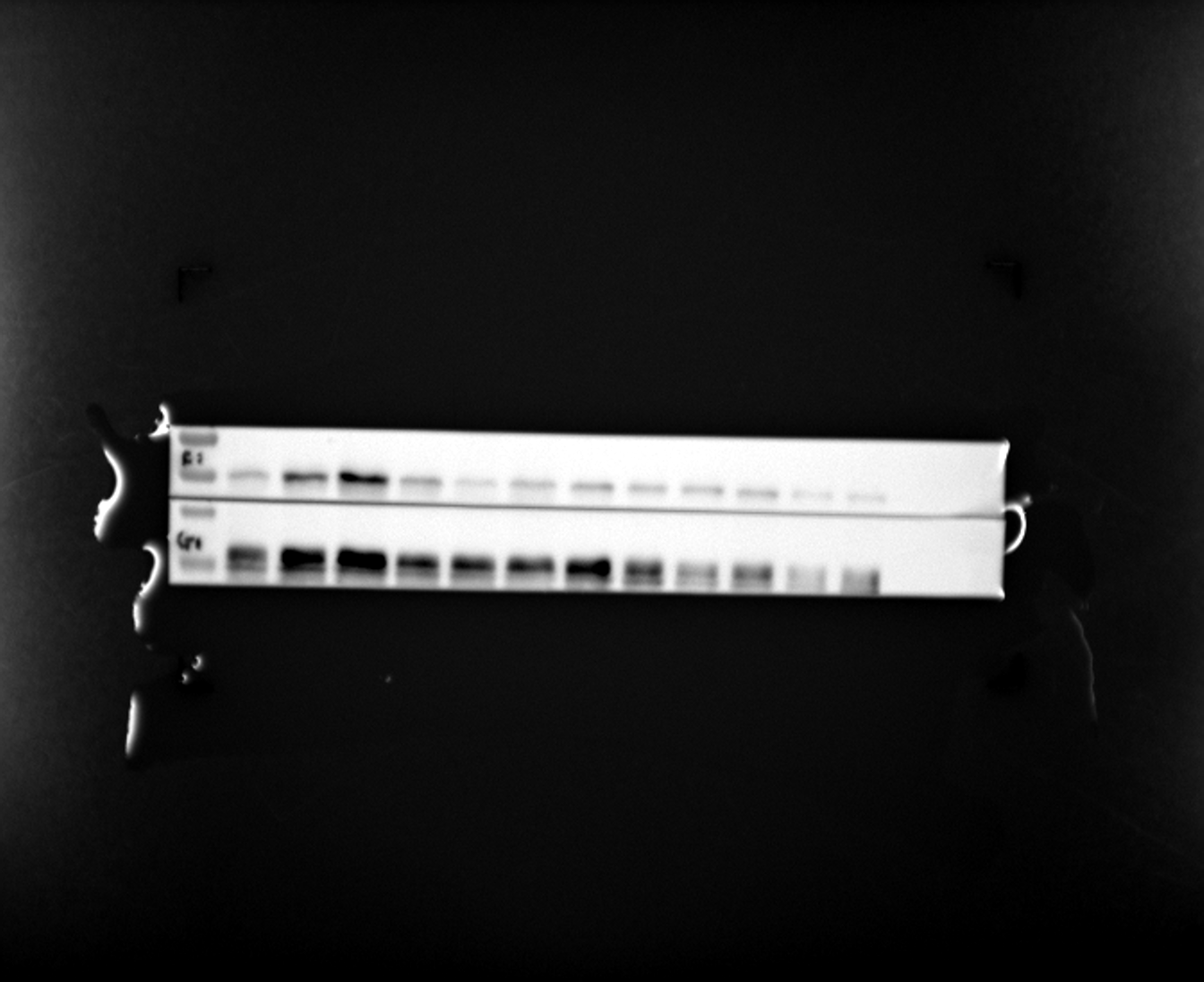

Supplement: Figure S5 [file peerj-11-16407-s008.zip › Figure 5/FXR&SHP protein/Liver-FXR-GAPDH/3/LIVER-12.5%-GAPDH-FXR-1-1.Tif]

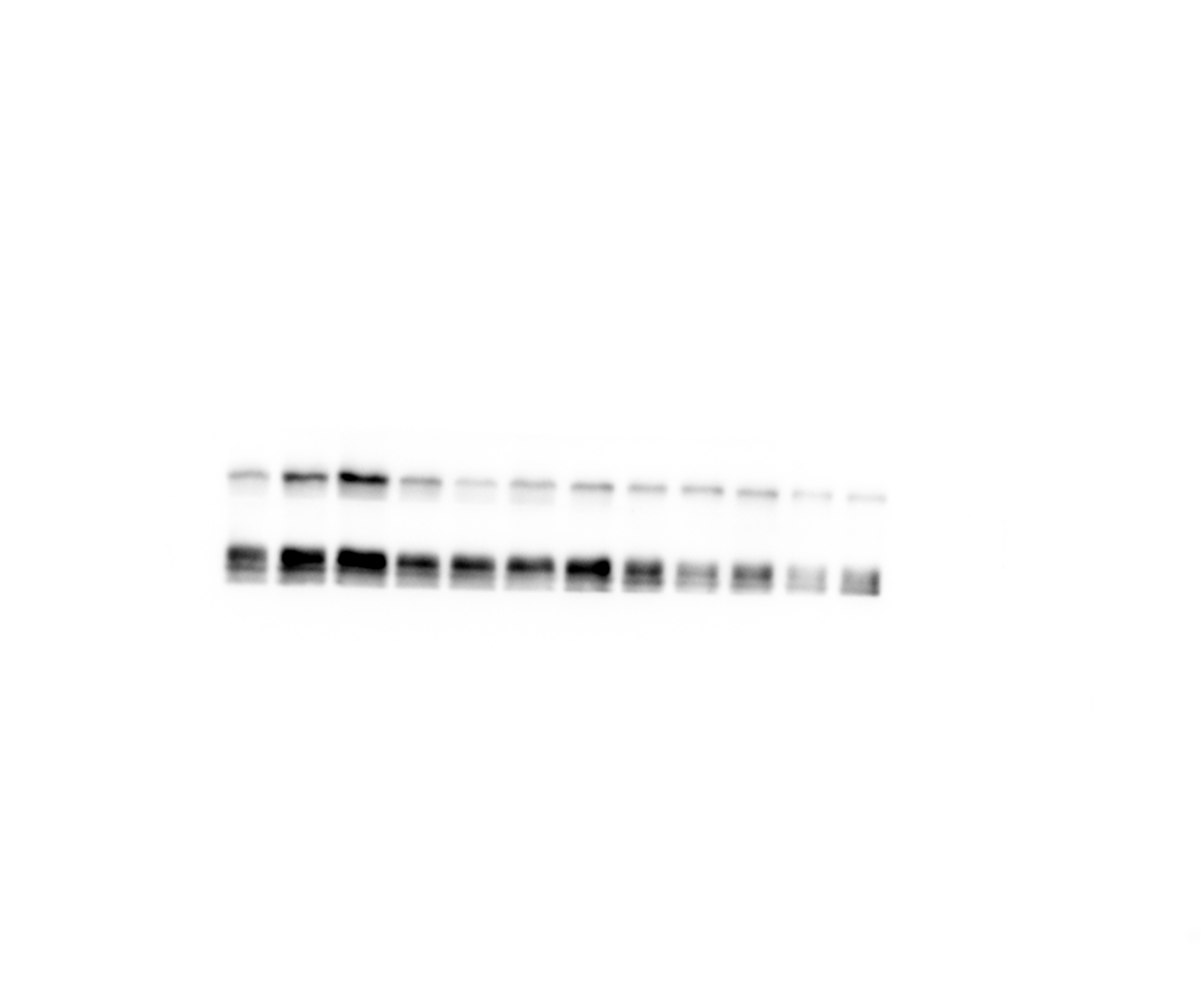

Supplement: Figure S5 [file peerj-11-16407-s008.zip › Figure 5/FXR&SHP protein/Liver-FXR-GAPDH/3/LIVER-12.5%-GAPDH-FXR-1.Tif]

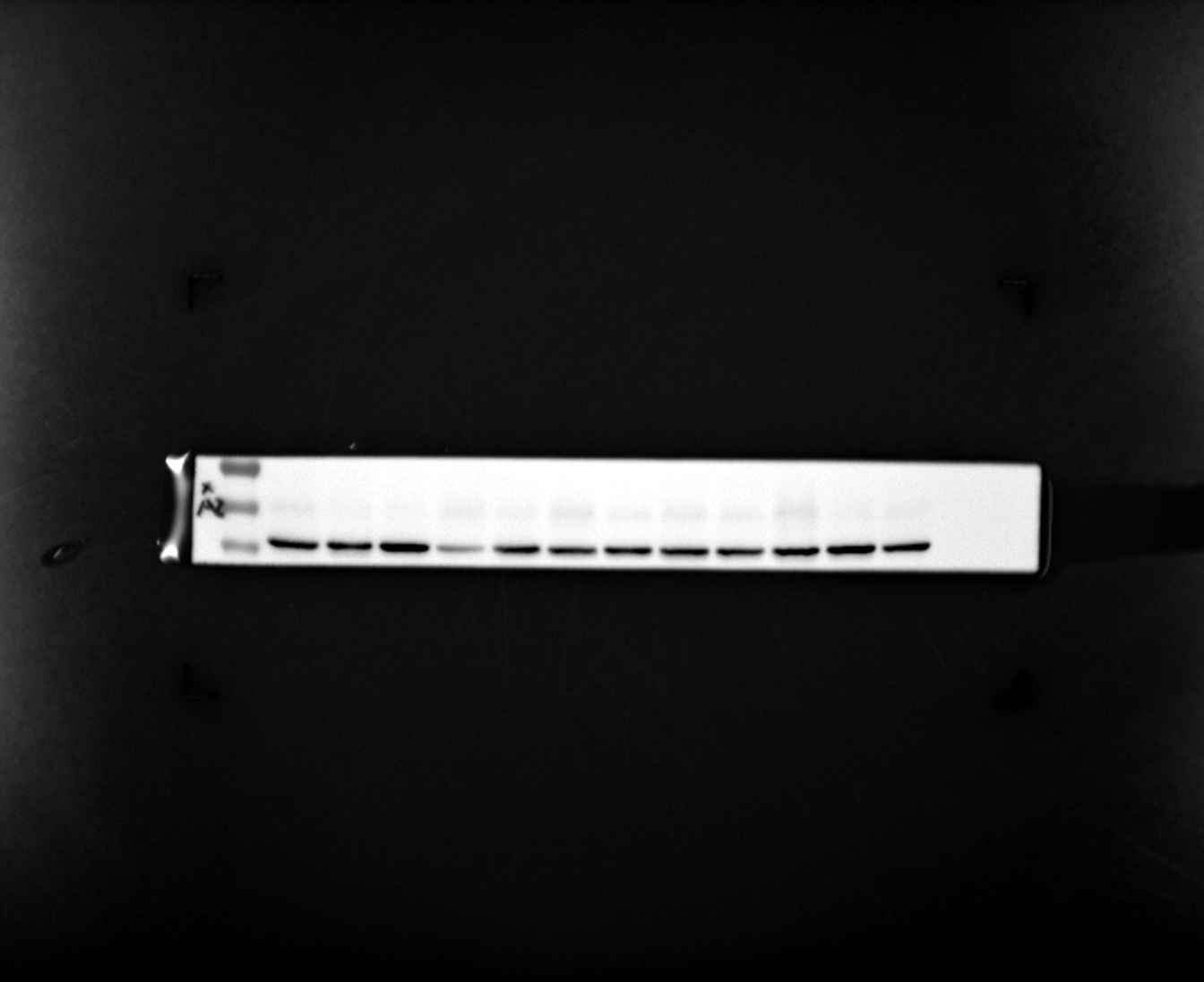

Supplement: Figure S5 [file peerj-11-16407-s008.zip › Figure 5/FXR&SHP protein/Liver-SHP-actin/1/12%-LIVER-ACTB-2-1.Tif]

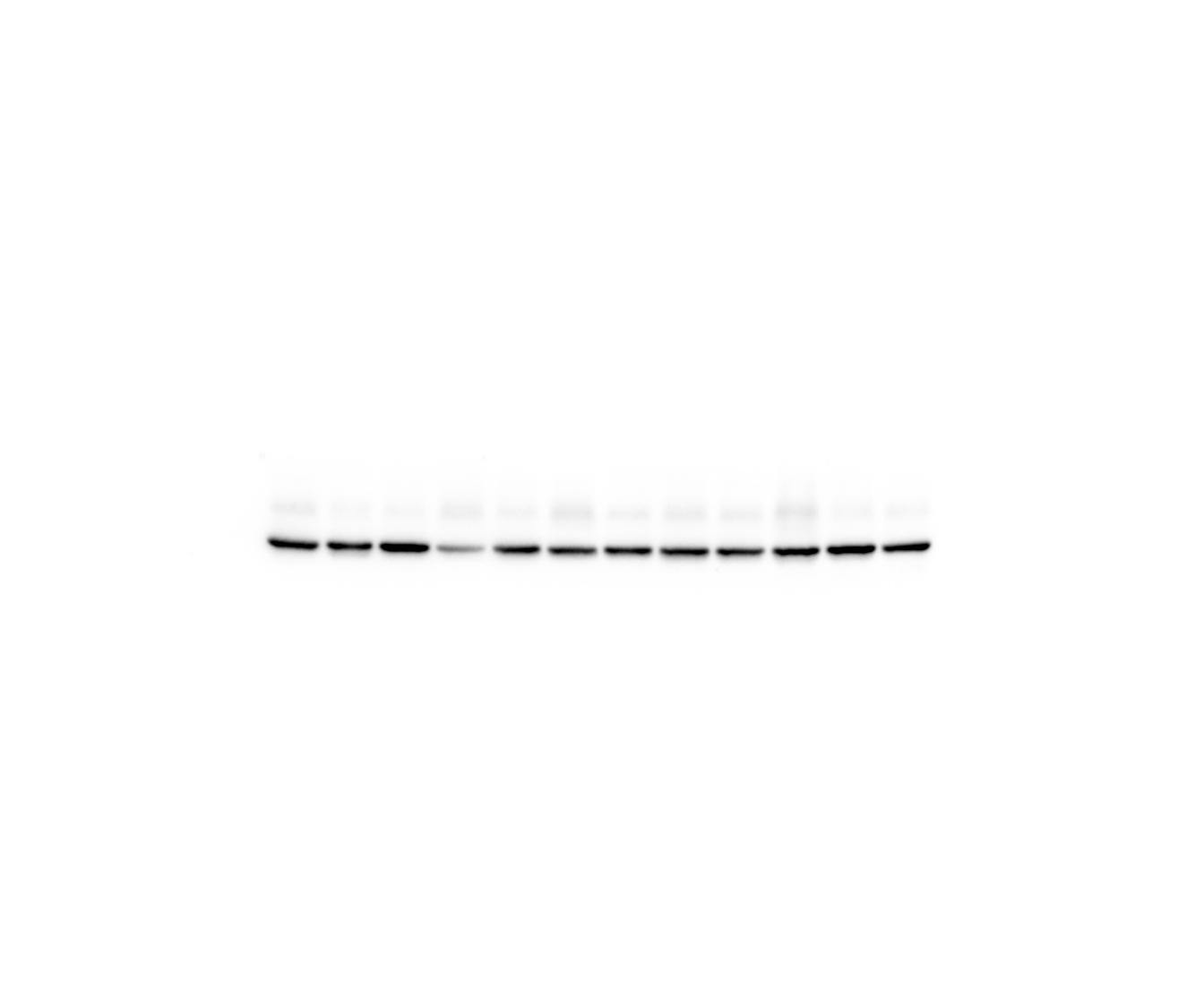

Supplement: Figure S5 [file peerj-11-16407-s008.zip › Figure 5/FXR&SHP protein/Liver-SHP-actin/1/12%-LIVER-ACTB-2.Tif]

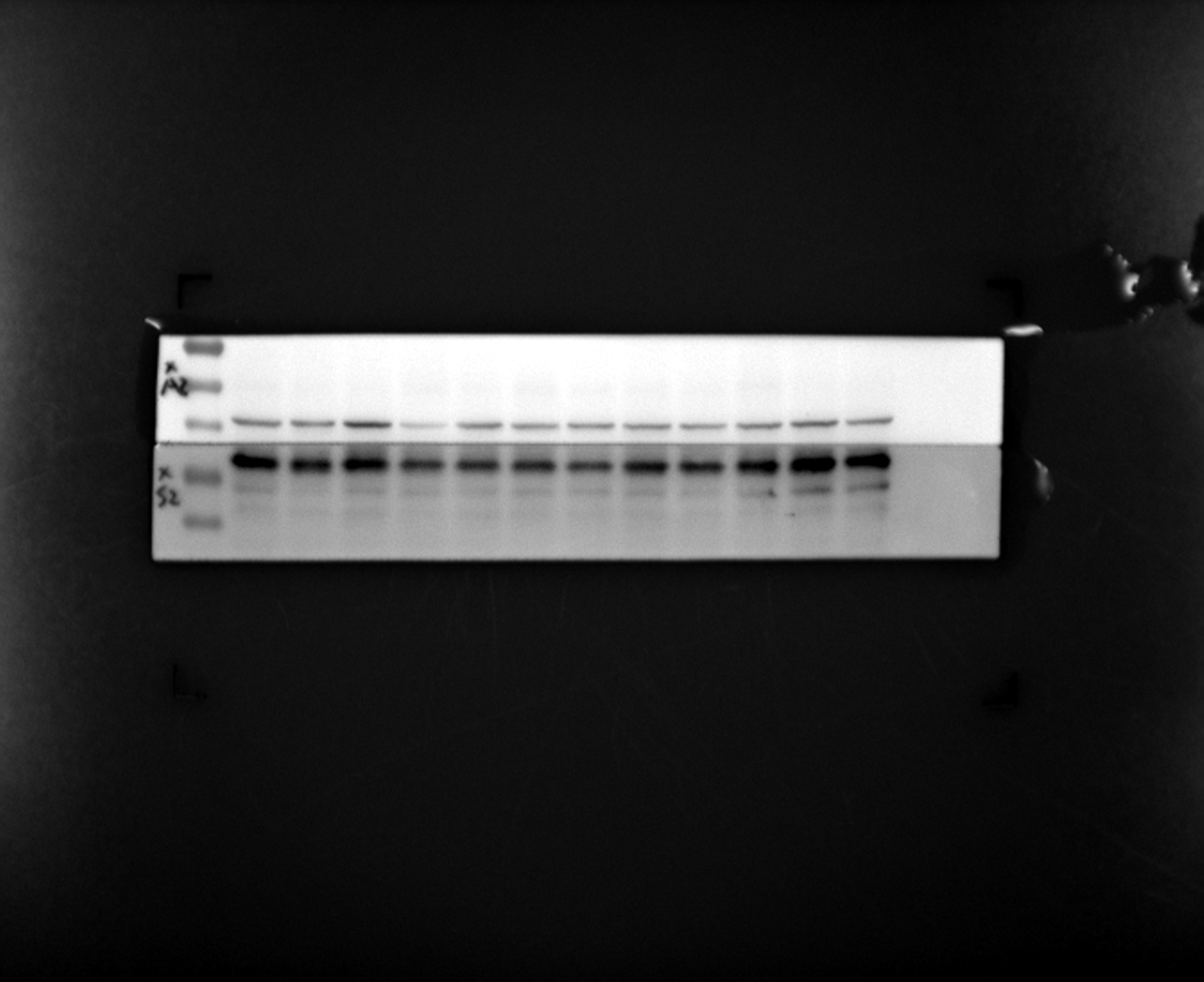

Supplement: Figure S5 [file peerj-11-16407-s008.zip › Figure 5/FXR&SHP protein/Liver-SHP-actin/1/12%-LIVER-ACTB-SHP-2-1.Tif]

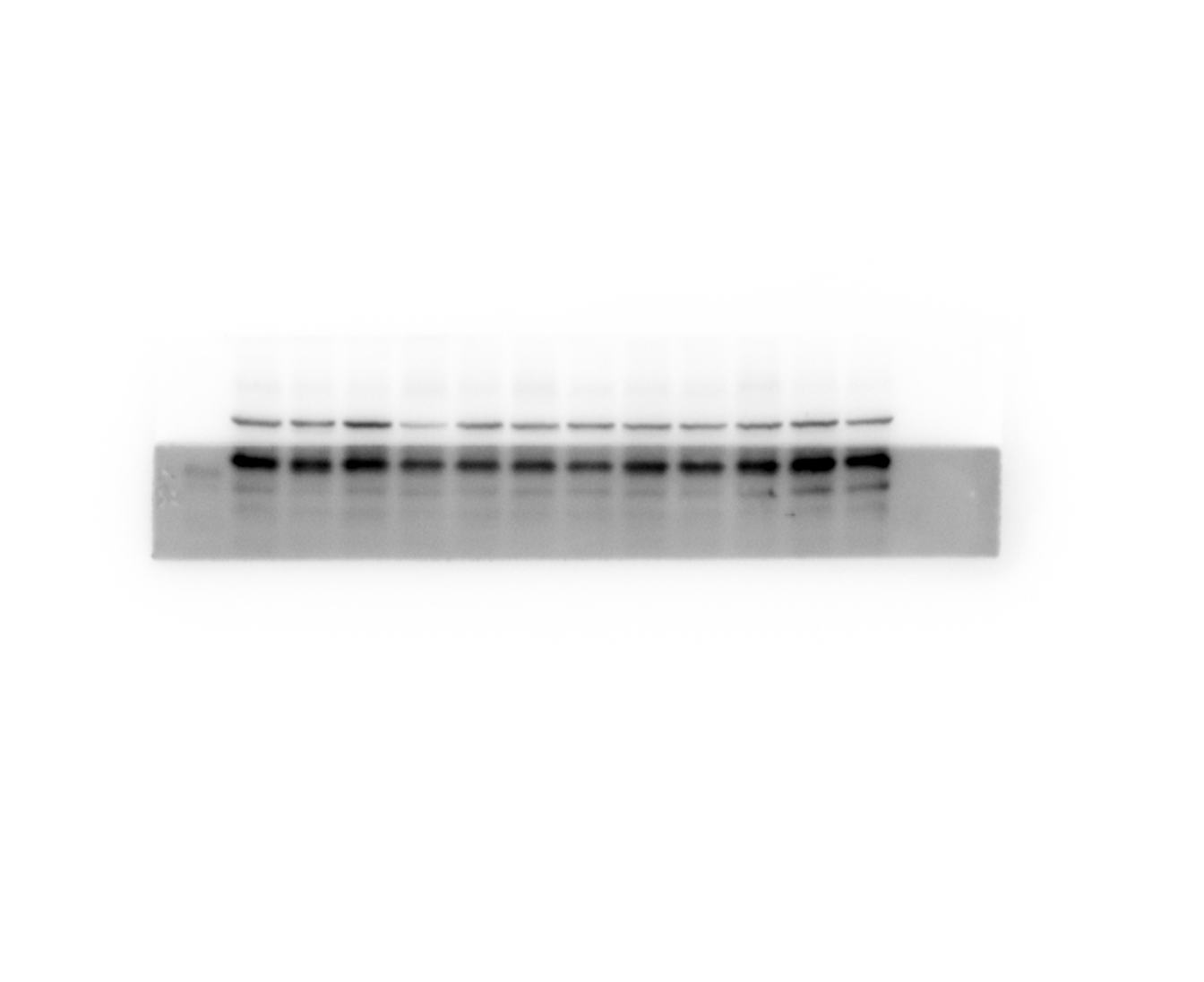

Supplement: Figure S5 [file peerj-11-16407-s008.zip › Figure 5/FXR&SHP protein/Liver-SHP-actin/1/12%-LIVER-ACTB-SHP-2.Tif]

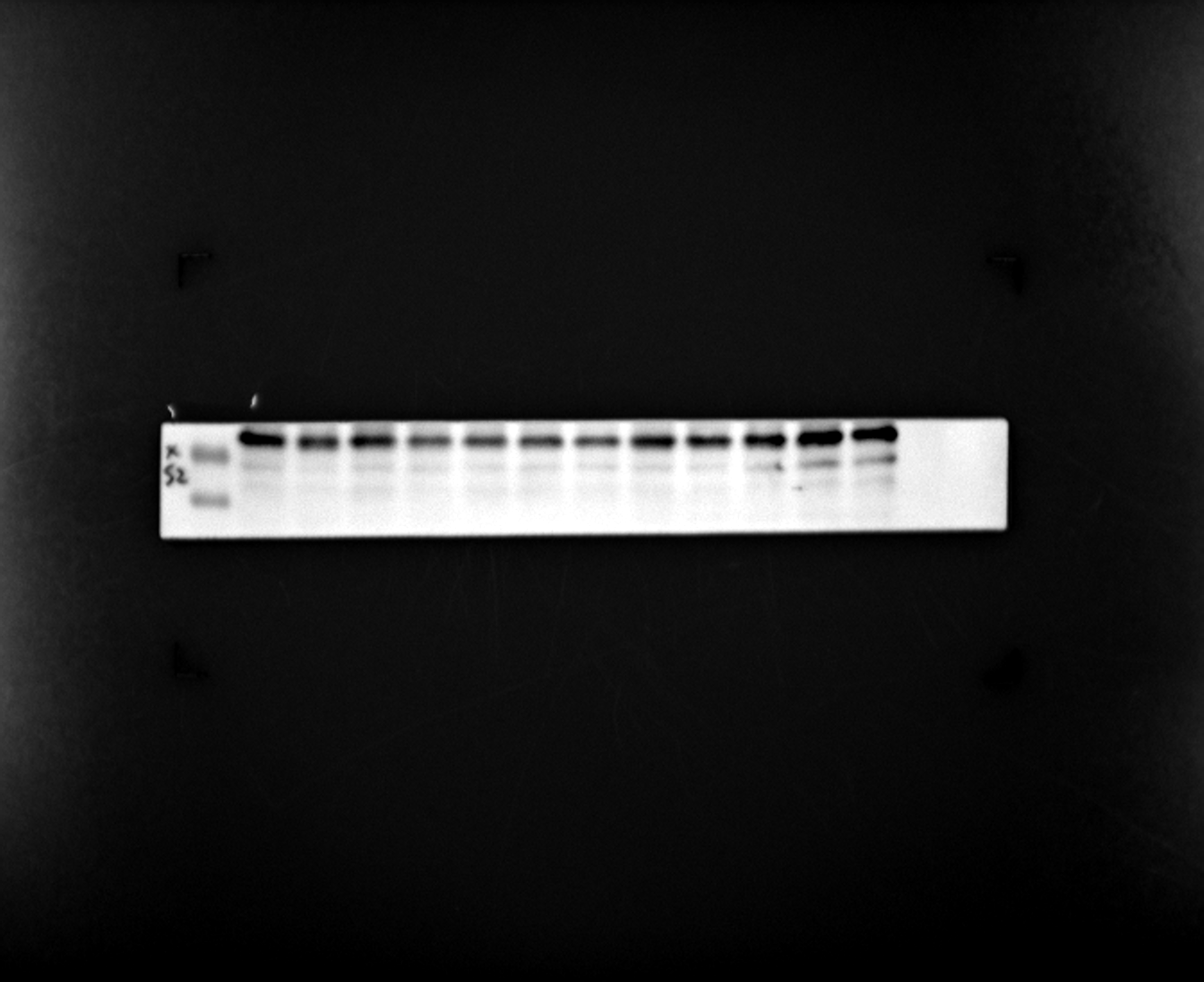

Supplement: Figure S5 [file peerj-11-16407-s008.zip › Figure 5/FXR&SHP protein/Liver-SHP-actin/1/12%-LIVER-SHP-2-1.Tif]

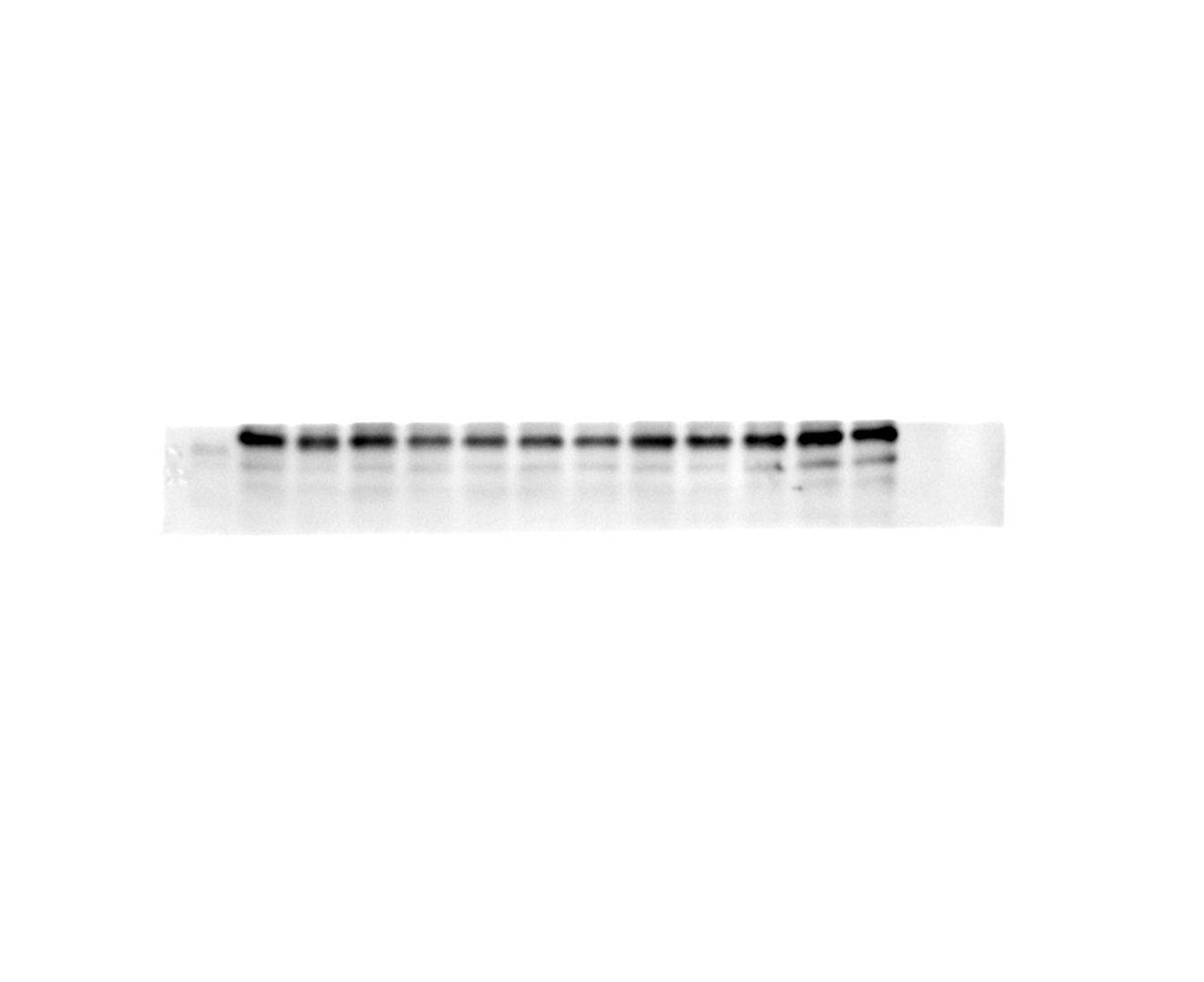

Supplement: Figure S5 [file peerj-11-16407-s008.zip › Figure 5/FXR&SHP protein/Liver-SHP-actin/1/12%-LIVER-SHP-2.Tif]

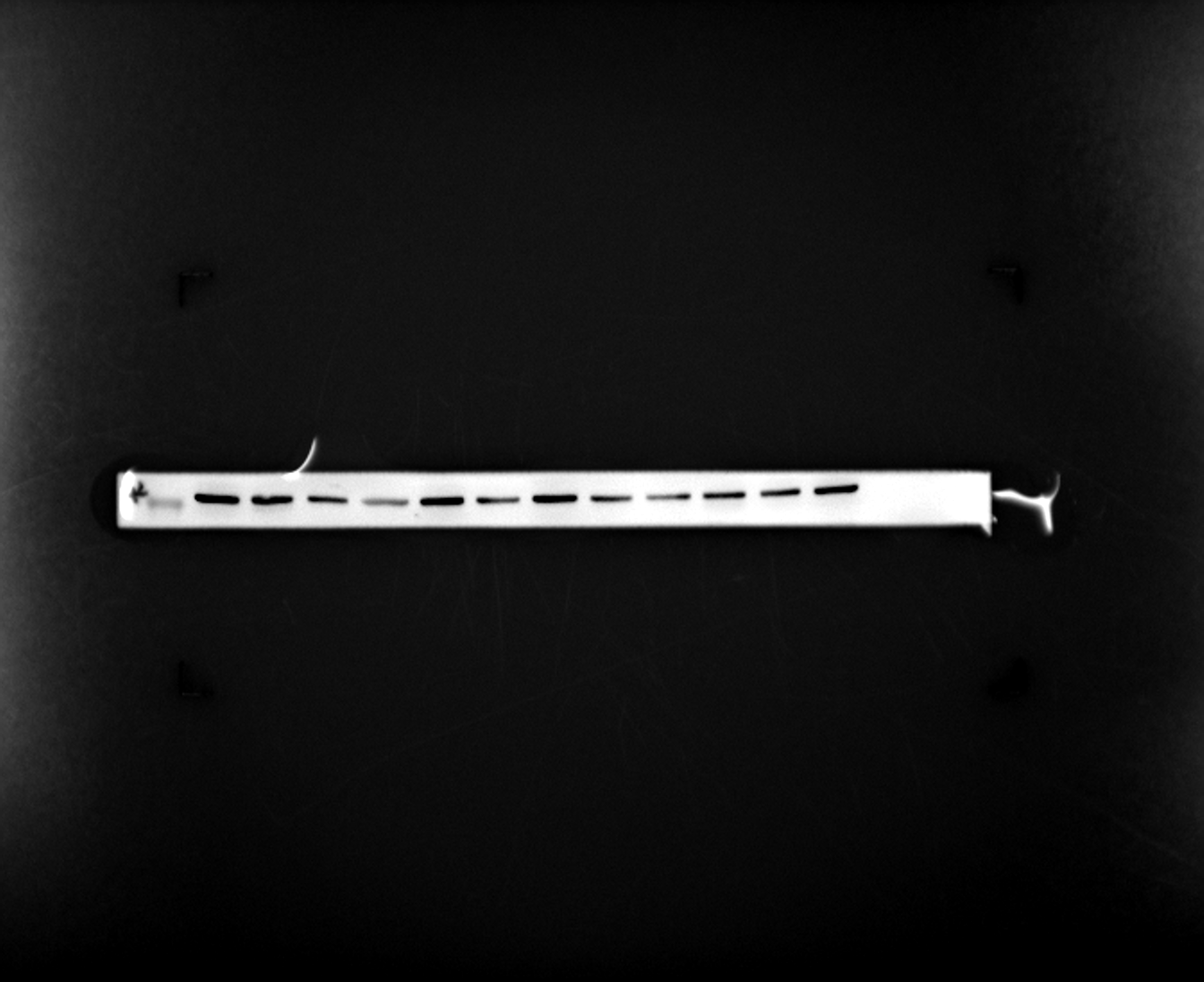

Supplement: Figure S5 [file peerj-11-16407-s008.zip › Figure 5/FXR&SHP protein/Liver-SHP-actin/2/12%-LIVER-ACTB-2-1.Tif]

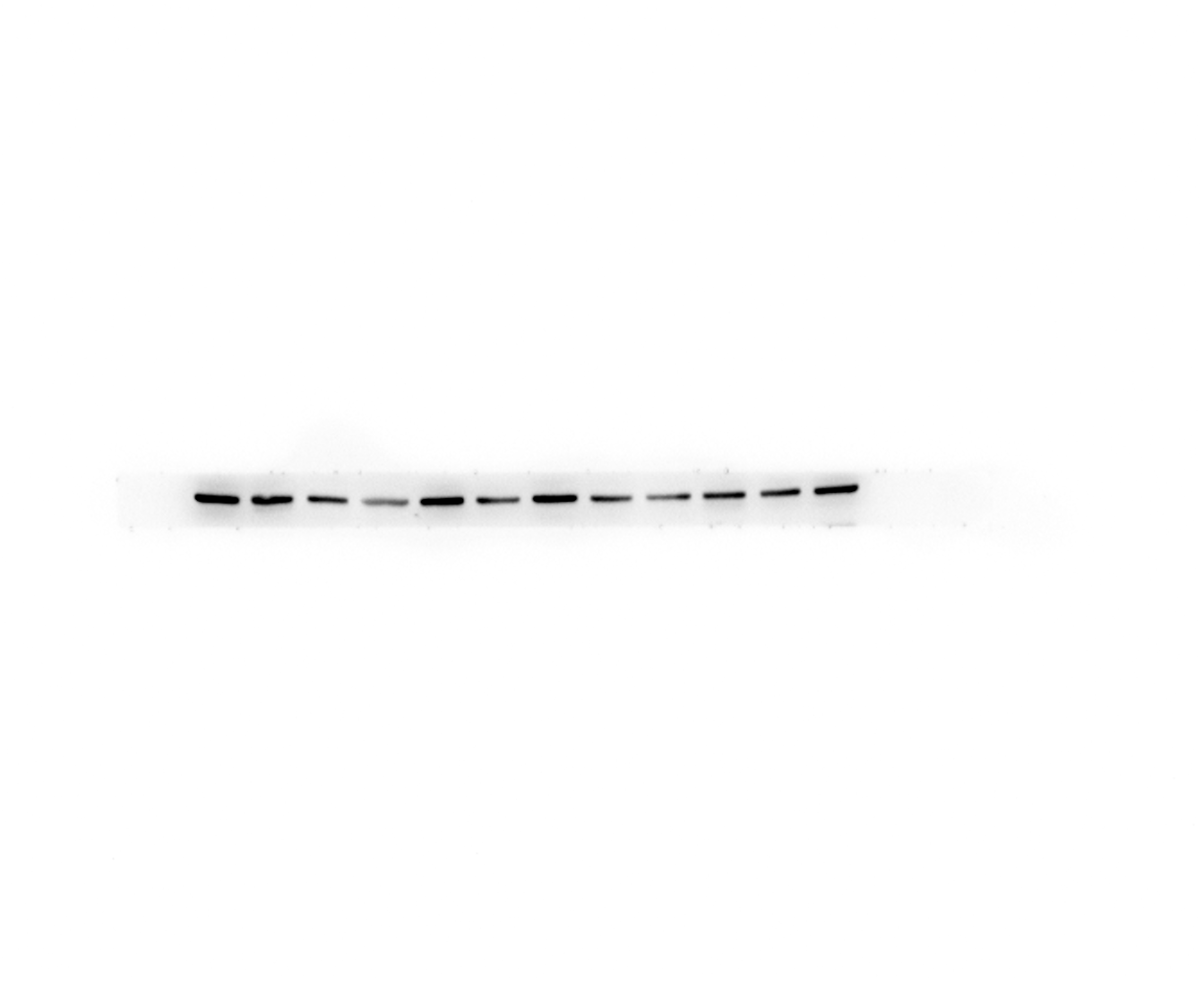

Supplement: Figure S5 [file peerj-11-16407-s008.zip › Figure 5/FXR&SHP protein/Liver-SHP-actin/2/12%-LIVER-ACTB-2.Tif]

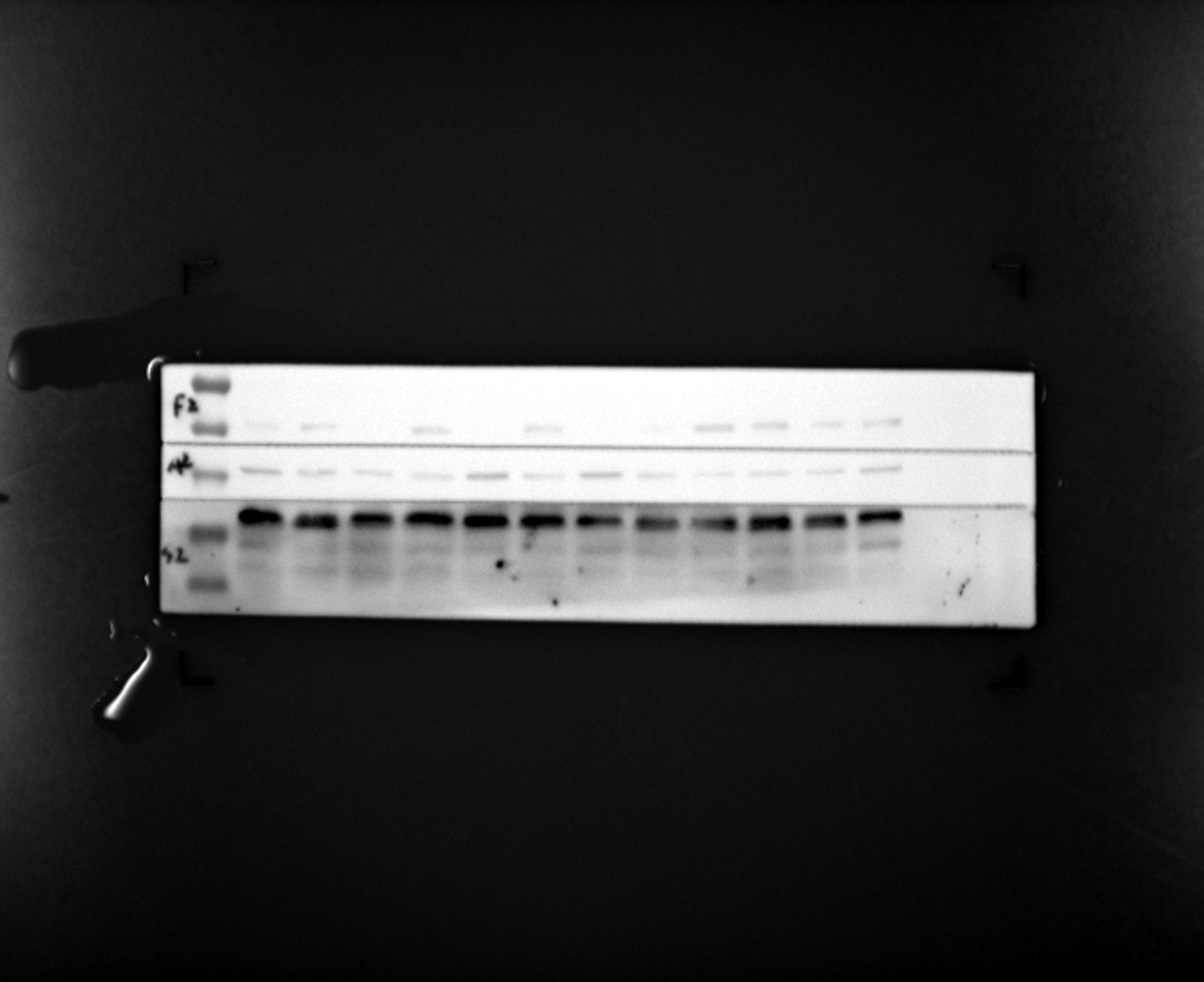

Supplement: Figure S5 [file peerj-11-16407-s008.zip › Figure 5/FXR&SHP protein/Liver-SHP-actin/2/12%-LIVER-ACTB-SHP-2-1.Tif]

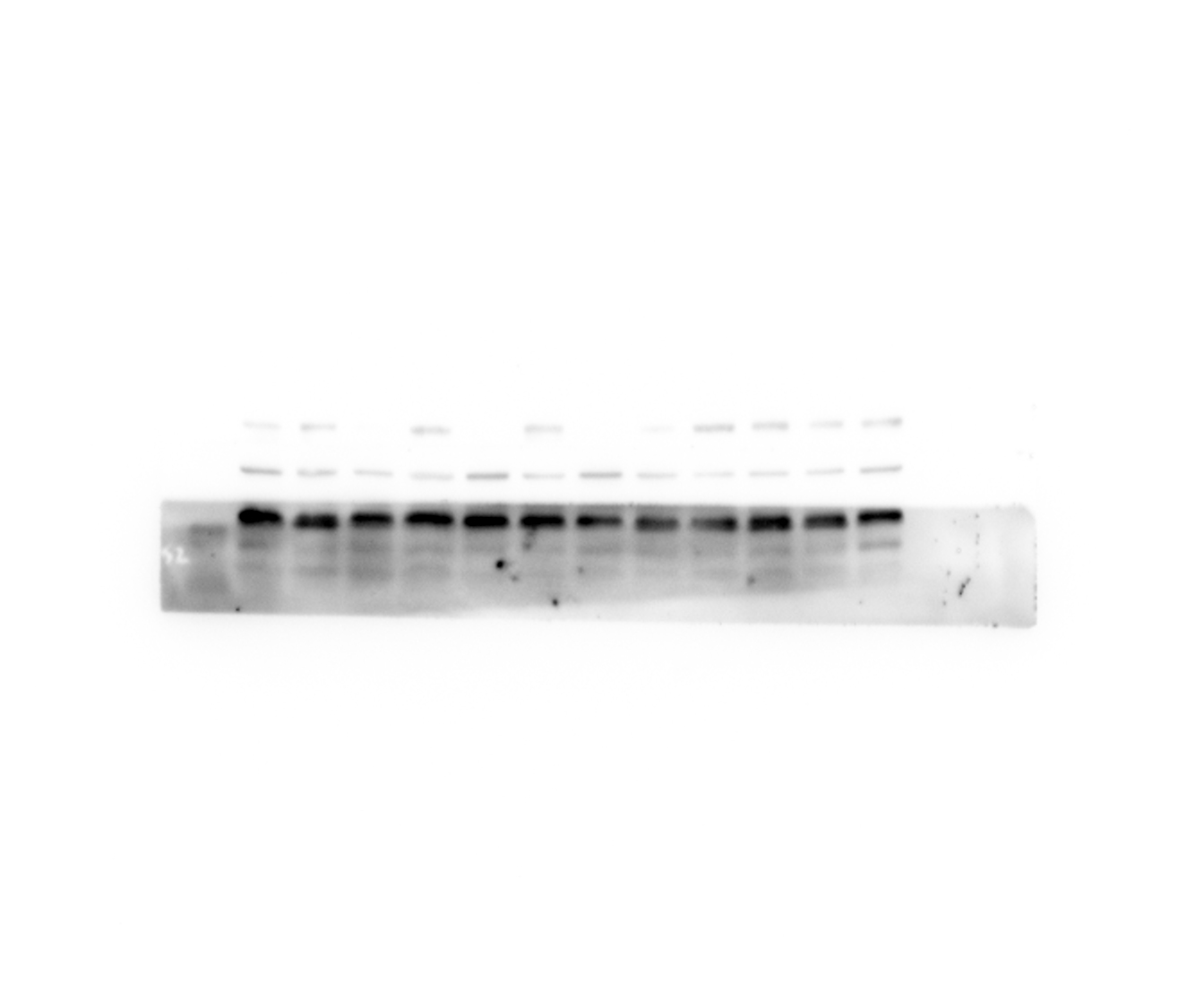

Supplement: Figure S5 [file peerj-11-16407-s008.zip › Figure 5/FXR&SHP protein/Liver-SHP-actin/2/12%-LIVER-ACTB-SHP-2.Tif]

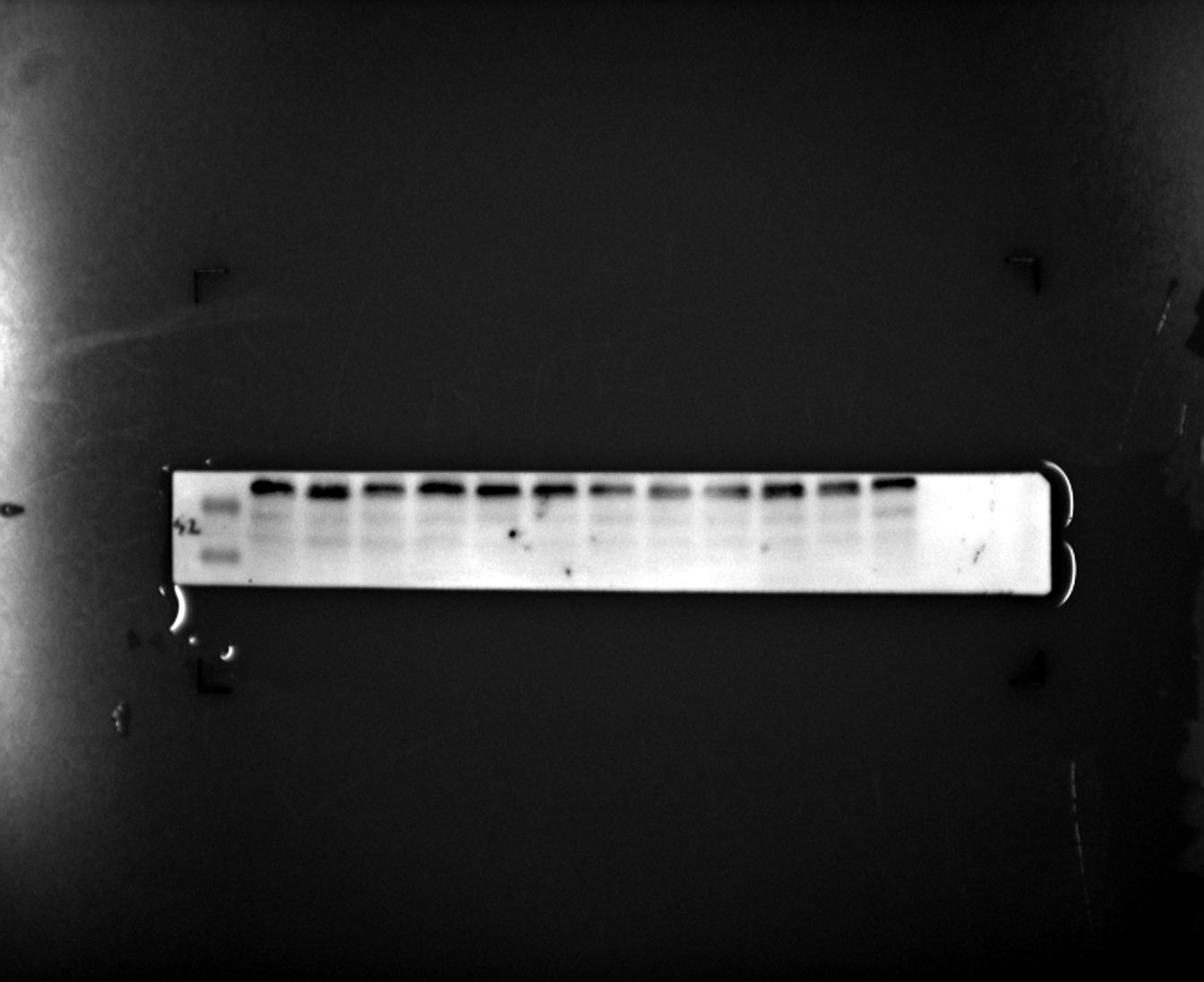

Supplement: Figure S5 [file peerj-11-16407-s008.zip › Figure 5/FXR&SHP protein/Liver-SHP-actin/2/12%-LIVER-SHP-2-1.Tif]

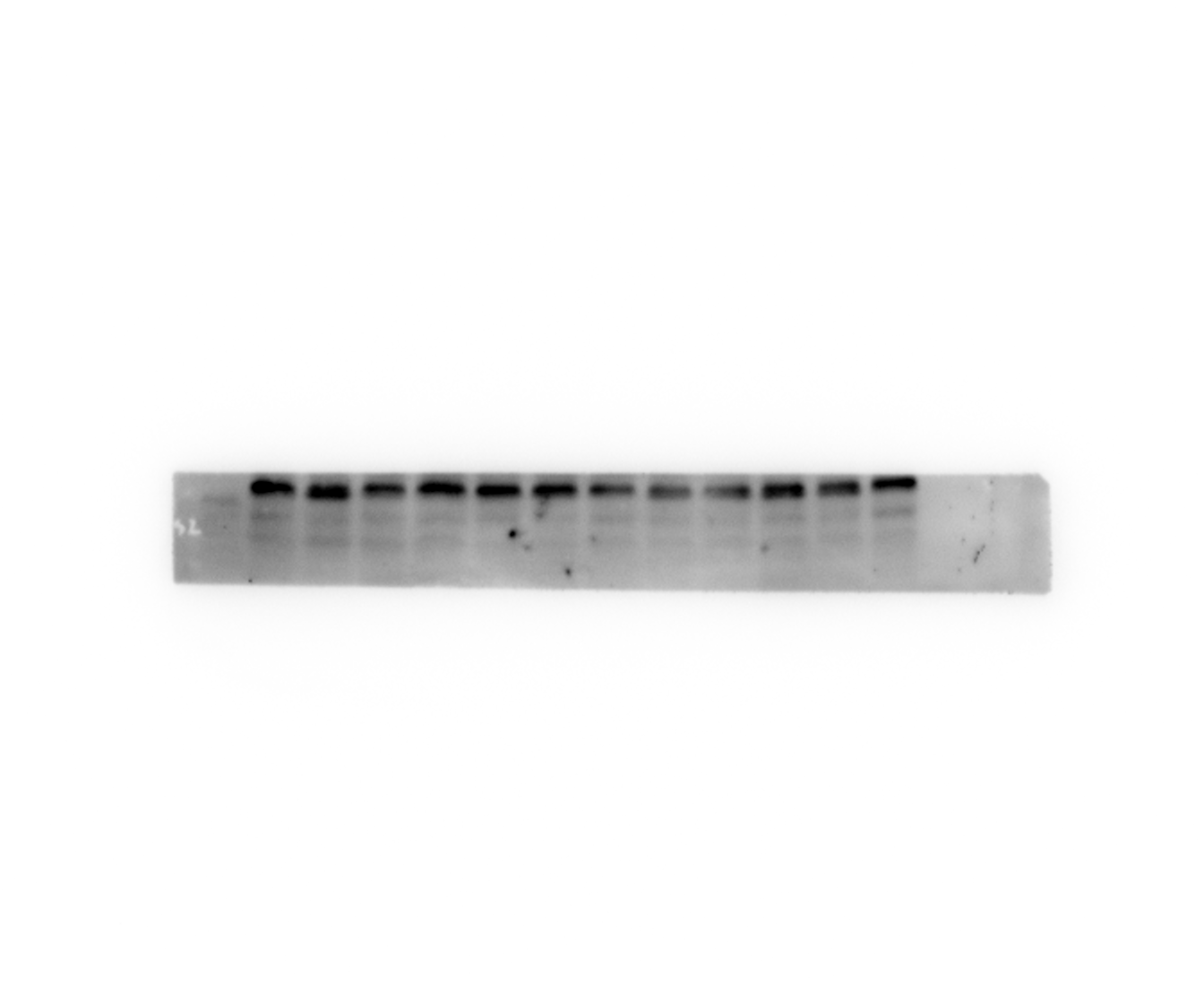

Supplement: Figure S5 [file peerj-11-16407-s008.zip › Figure 5/FXR&SHP protein/Liver-SHP-actin/2/12%-LIVER-SHP-2.Tif]

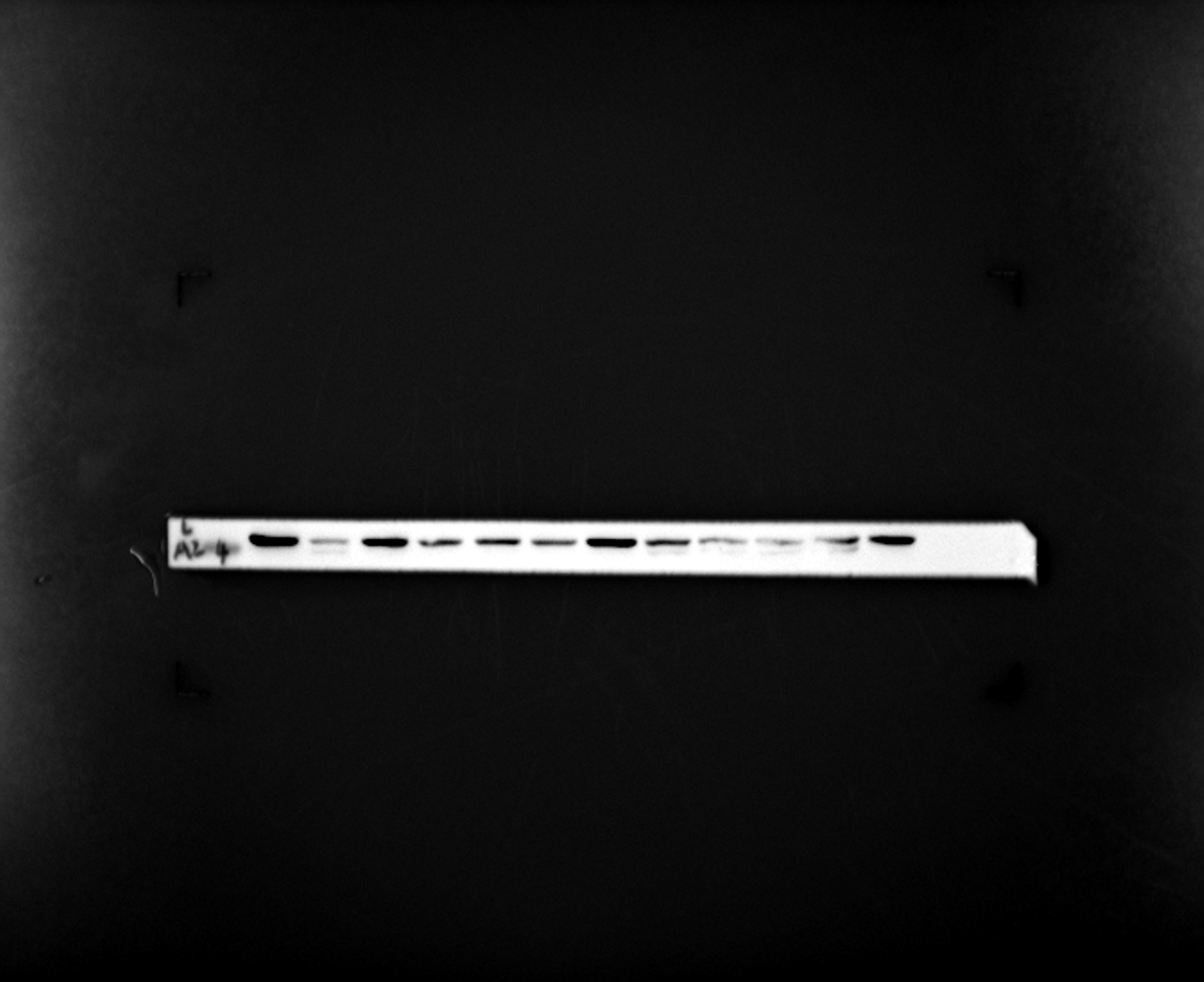

Supplement: Figure S5 [file peerj-11-16407-s008.zip › Figure 5/FXR&SHP protein/Liver-SHP-actin/3/12.5%-LIVER-ACTB-2-1.Tif]

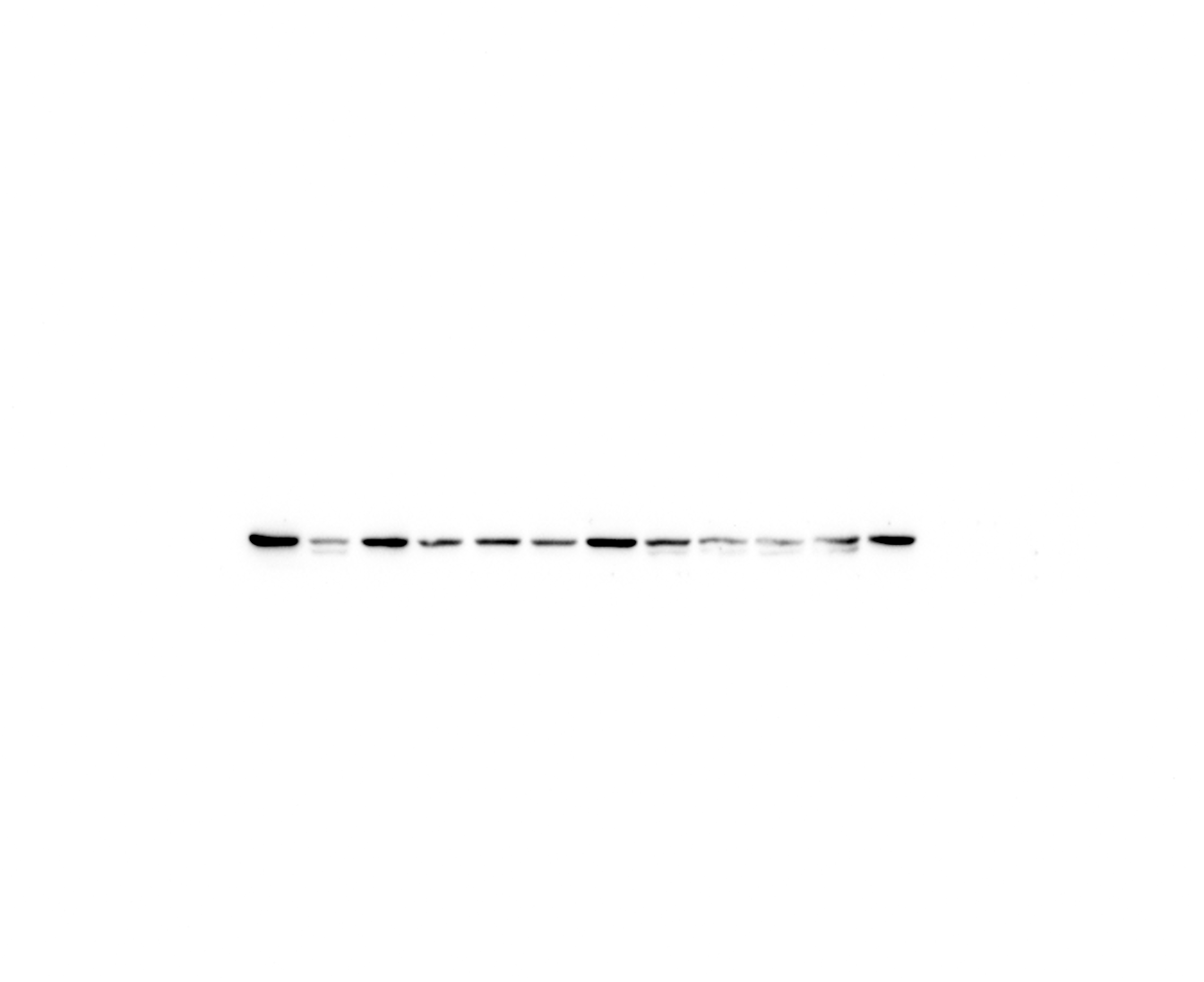

Supplement: Figure S5 [file peerj-11-16407-s008.zip › Figure 5/FXR&SHP protein/Liver-SHP-actin/3/12.5%-LIVER-ACTB-2.Tif]

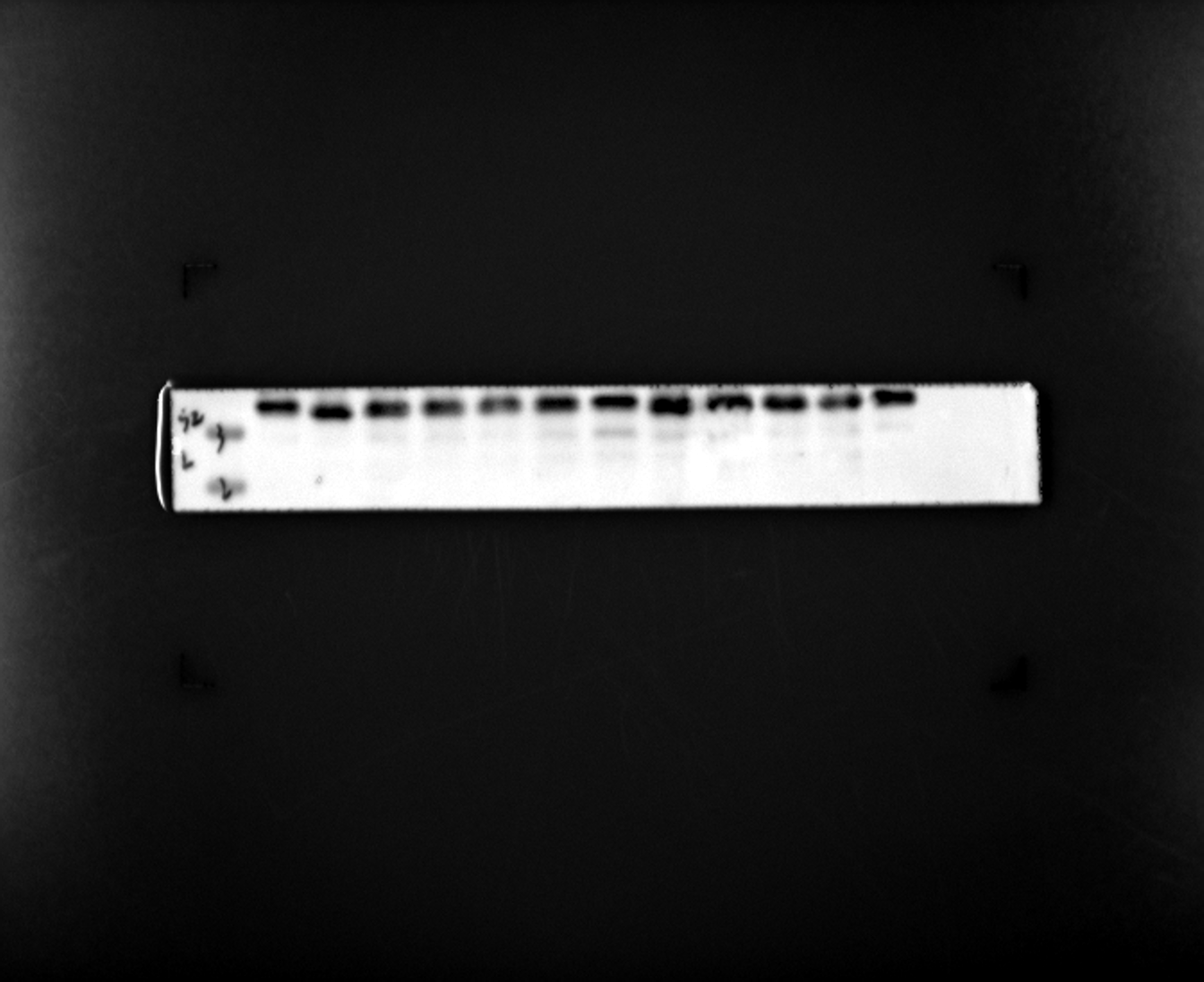

Supplement: Figure S5 [file peerj-11-16407-s008.zip › Figure 5/FXR&SHP protein/Liver-SHP-actin/3/12.5%-LIVER-SHP-2-1.Tif]

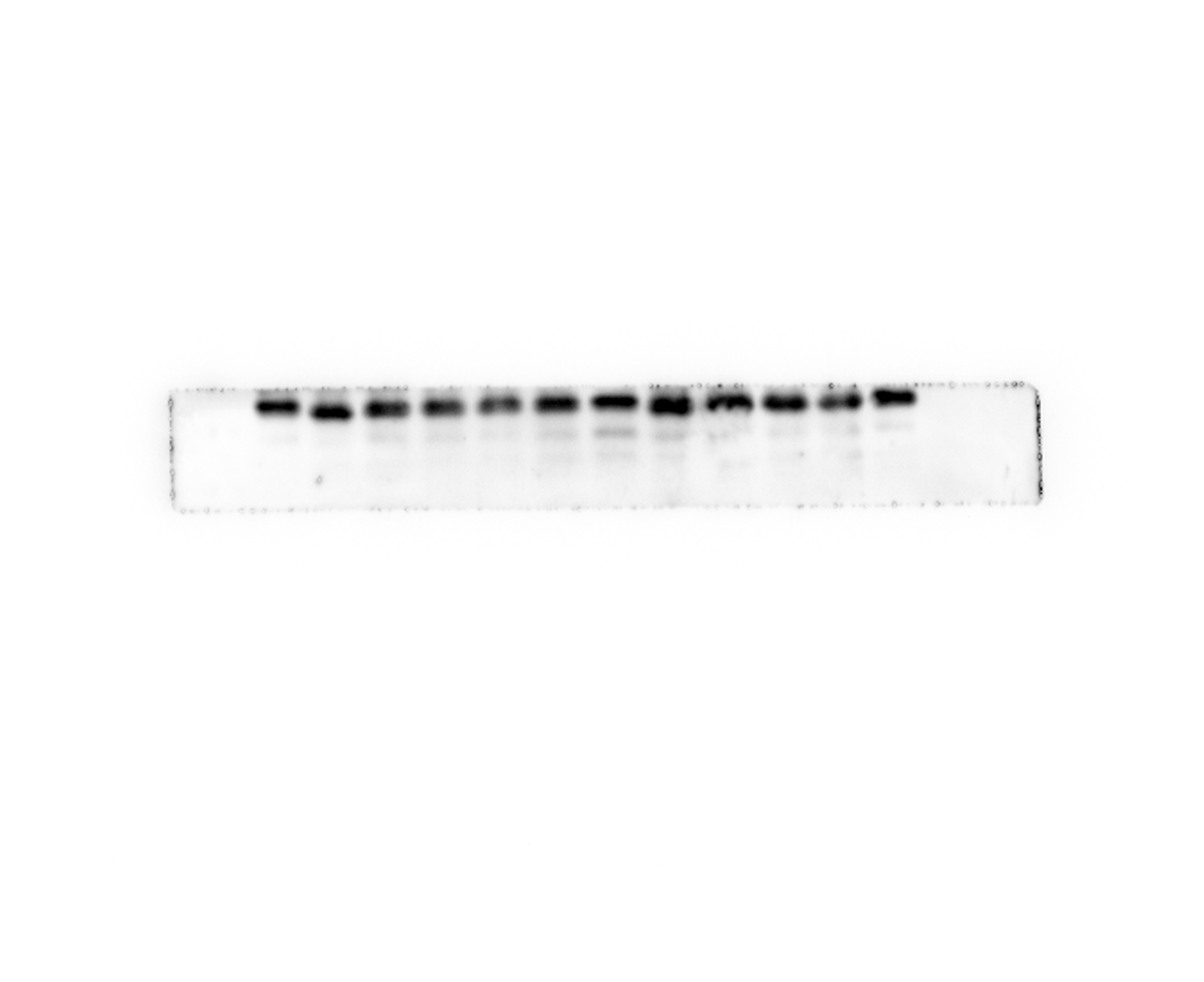

Supplement: Figure S5 [file peerj-11-16407-s008.zip › Figure 5/FXR&SHP protein/Liver-SHP-actin/3/12.5%-LIVER-SHP-2.Tif]
